# Supplementary figures and images for: Unusual bromine enrichment in the gastric mill and setae of the hadal amphipod Hirondellea gigas
Source: PLoS One. 2022 Aug 4;17(8):e0272032. doi: 10.1371/journal.pone.0272032 (PMC9352070; doi:10.1371/journal.pone.0272032)

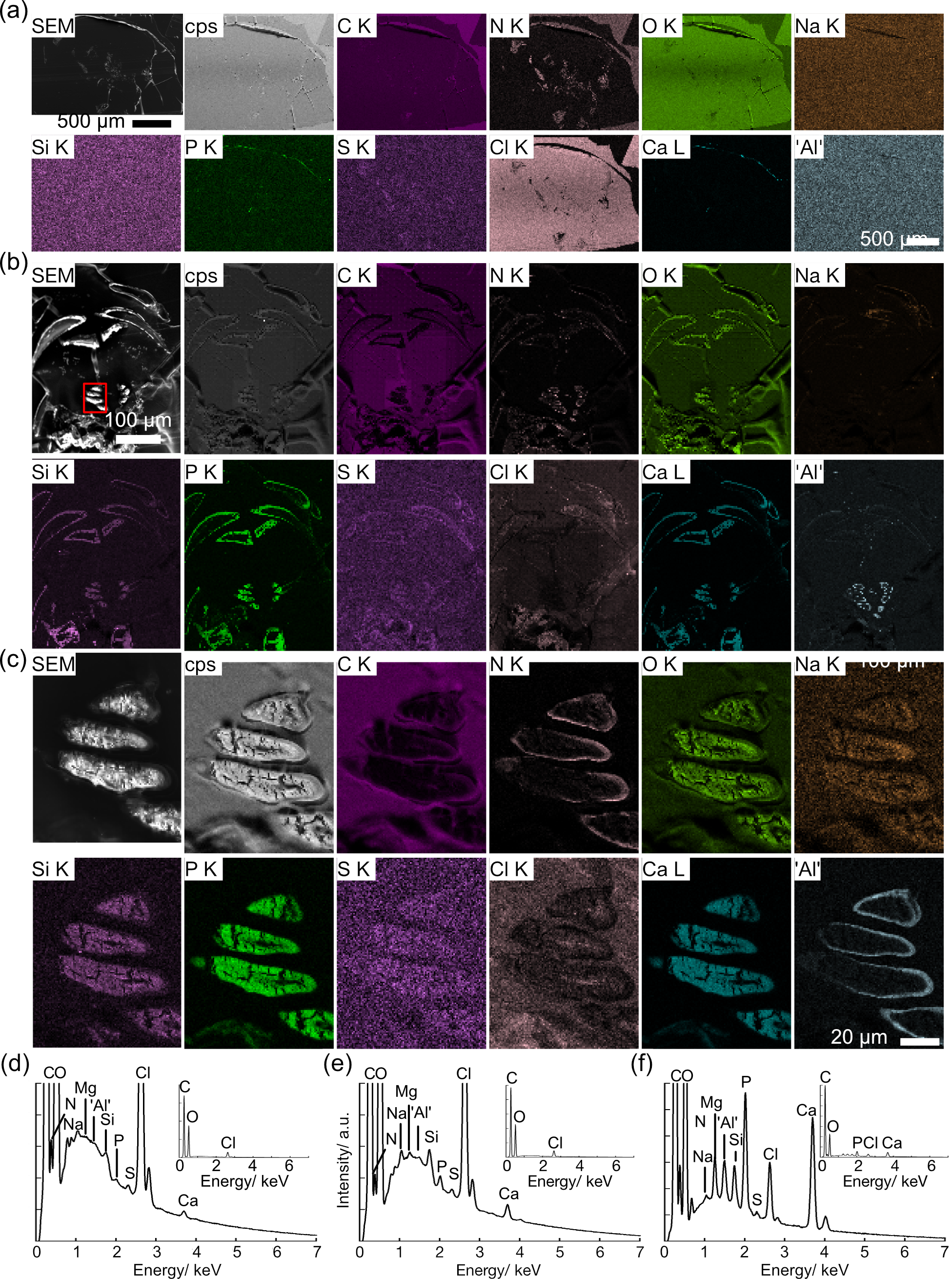

Supplement: S1 Fig — a) Transverse section through a subsample of pereonite. b) Transverse section of the cephalic region showing the gastric mill. c) Magnified images of the gastric mill, the red rectangle area in (b). SEM images, total X-ray counts (cps), and elemental maps are shown, and the labels are denoted in the left top. ‘Al’ corresponds to the aluminum-like signal around 1.48 keV, that overlaps with bromine L-line, and thulium and ytterbium M-line. d–f) EDS sum spectra of the (d) pereonite, (e) cephalic region, and (f) gastric mill created from the EDS maps (a)–(c). SEM images in (a) were acquired at 1 kV, and other images and spectra were acquired at 10 kV. (TIF) [file pone.0272032.s001.tif]

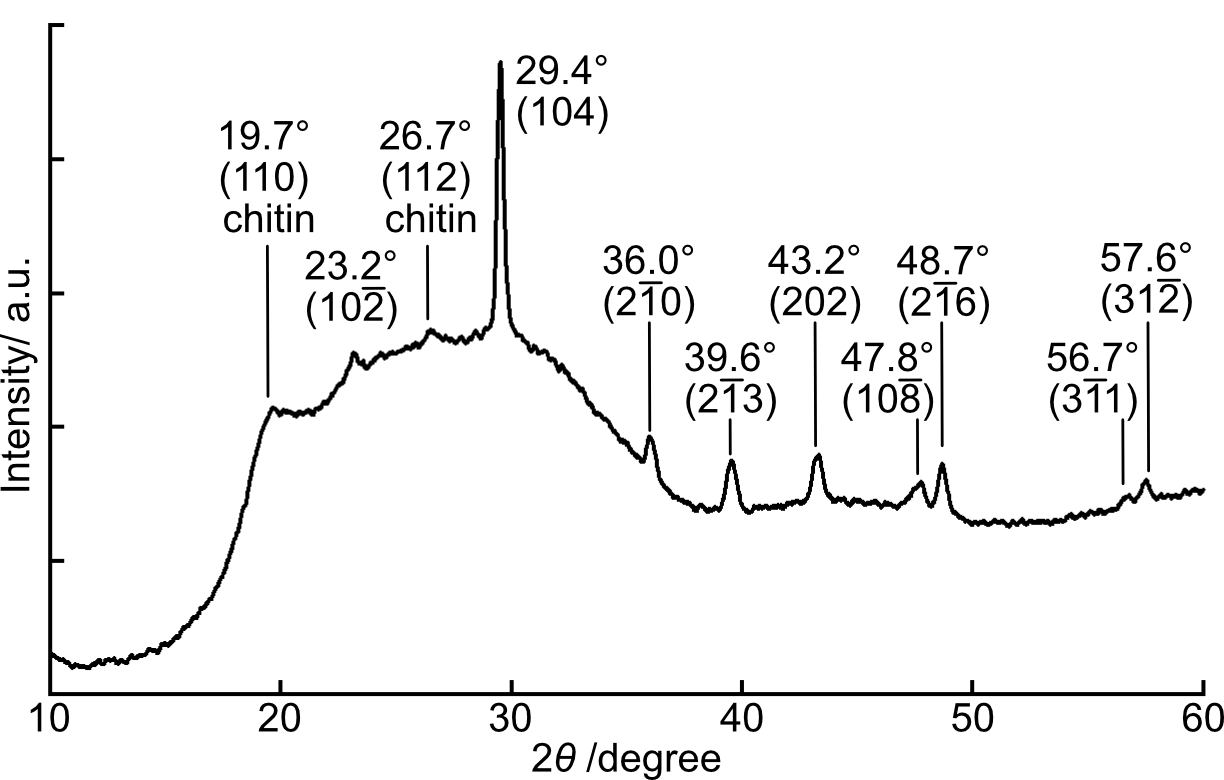

Supplement: S2 Fig — Diffraction peak position in 2θ and corresponding crystal planes of calcite and α-chitin in parenthesis are shown. (TIF) [file pone.0272032.s002.tif]

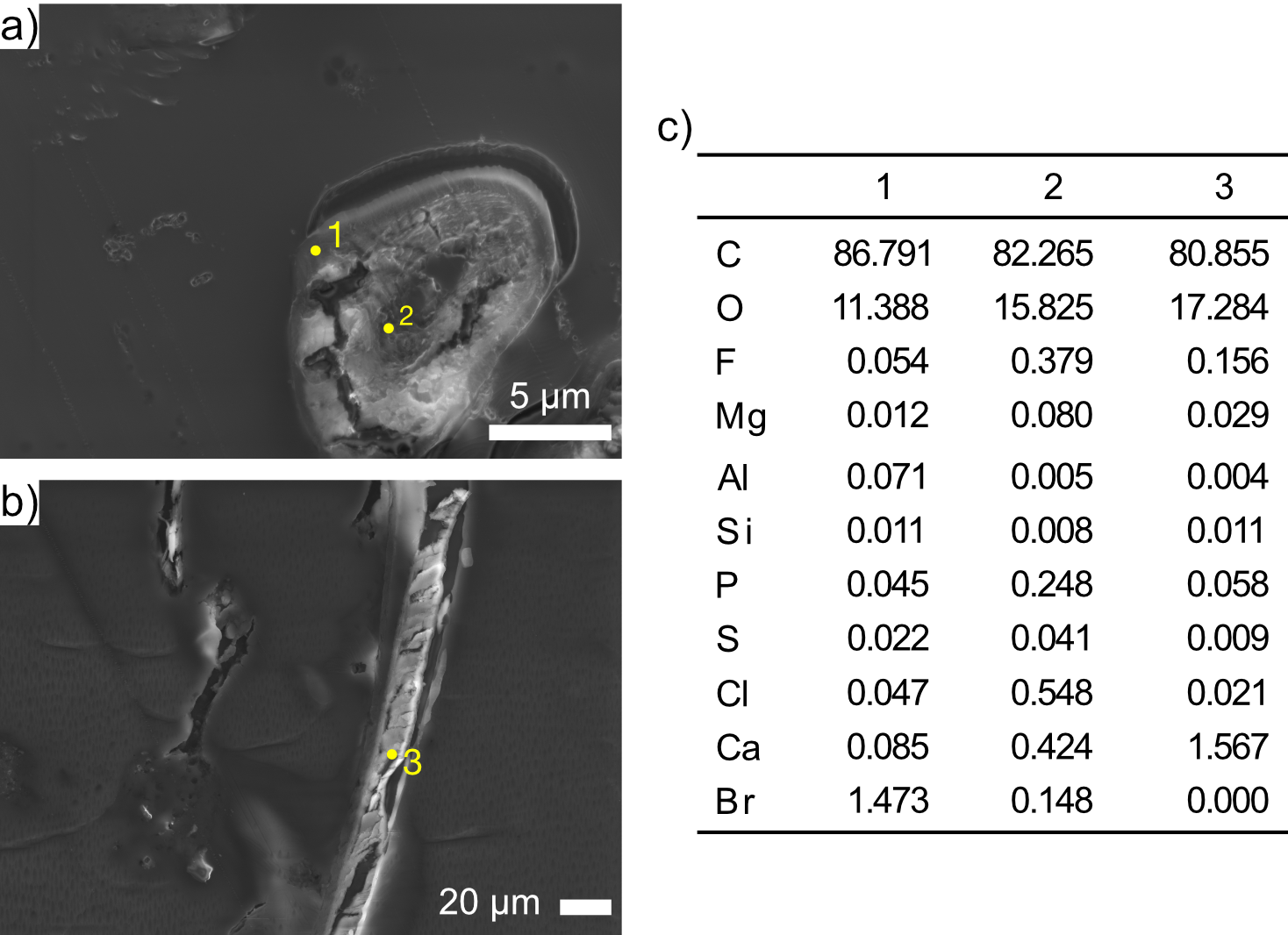

Supplement: S3 Fig — a) SEM images of the gastric mill. b) SEM image of the pereonite. c) Atomic ratio of the elements. K-line of the elements was used for the quantification. The large value of carbon and oxygen is ascribed to the conductive carbon coating and the underlying Kapton tape. Note that the amount of aluminum was <5% of that of bromine, and may contain some error because of the overlap of Al K-line with Br L-line, which is not completely separable under WDS conditions (Fig 2B). Nitrogen was also searched but not observed at any points (0.00), thus not shown. (TIF) [file pone.0272032.s003.tif]

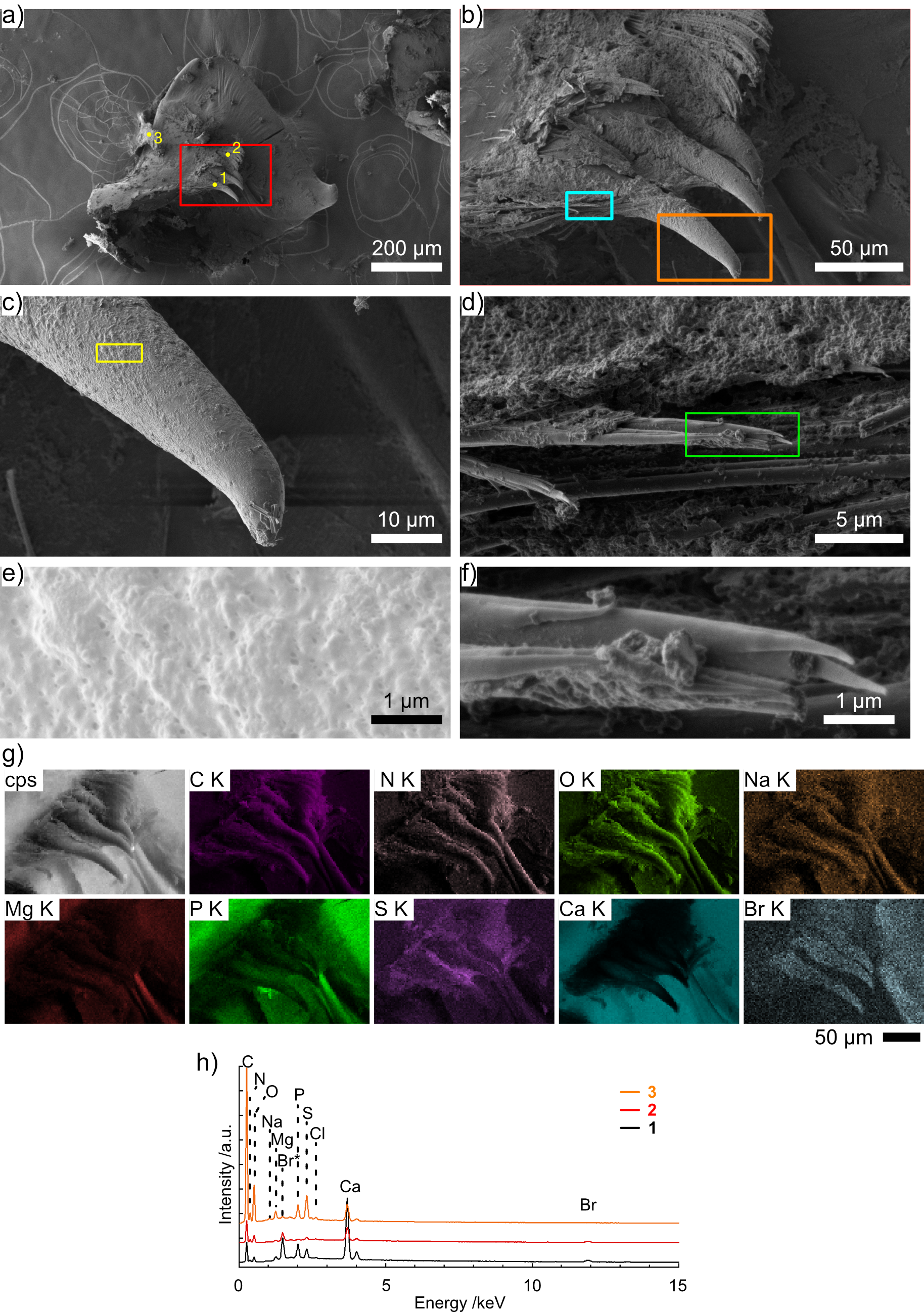

Supplement: S4 Fig — a) Low-magnification SEM image. b) Magnified image of the red rectangle in (a). c) Magnified image of the orange rectangle in b). d) Magnified image of the blue rectangle in (b). e) Magnified image of the yellow rectangle in (c). f) Magnified image of the green rectangle in (d). g) EDS elemental mapping of the red rectangle in (a). h) EDS spectra of the yellow points in (a). Atom labels correspond to K-line except for Br*, which is L-line. (TIF) [file pone.0272032.s004.tif]

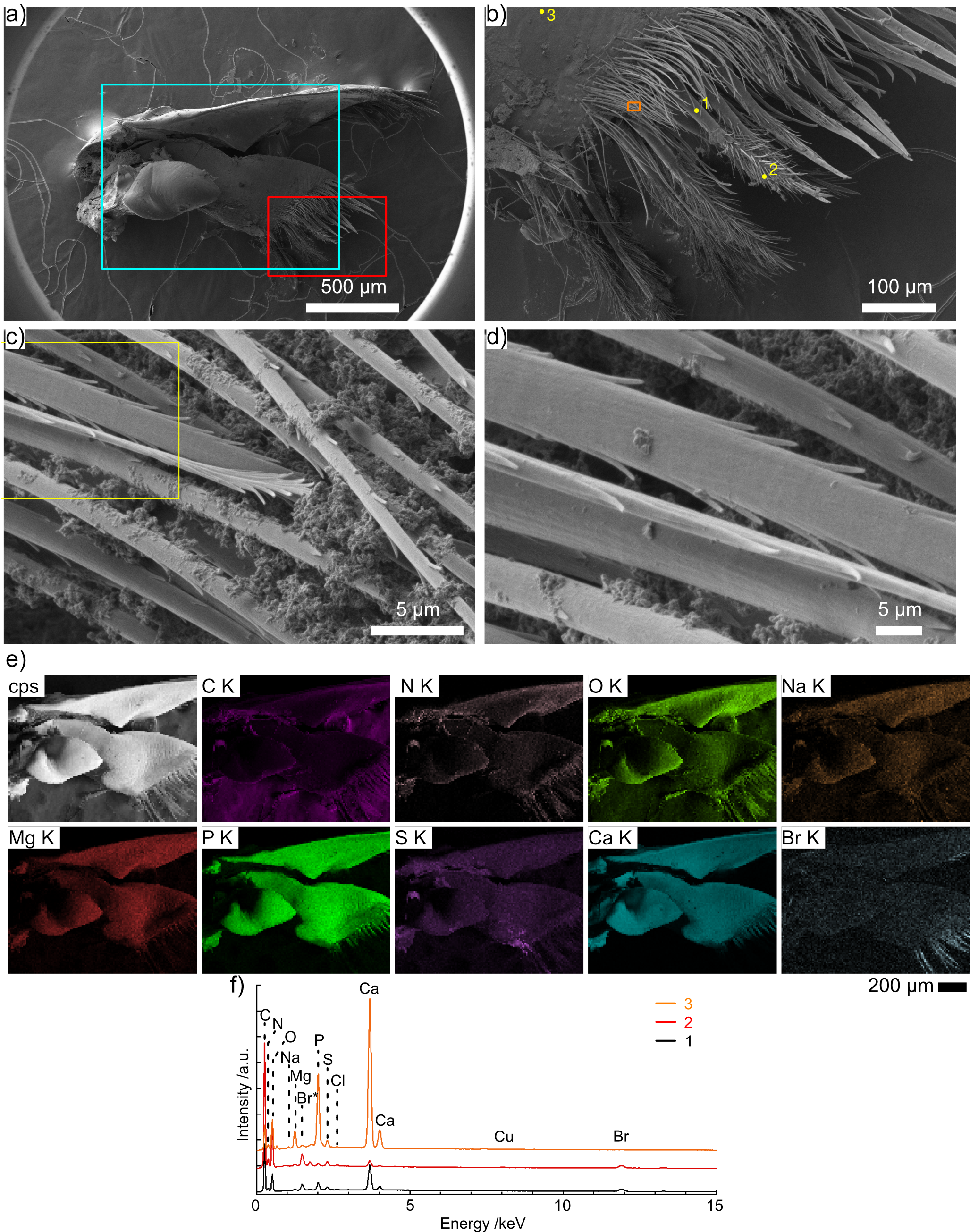

Supplement: S5 Fig — a) Low-magnification SEM image. b) Magnified image of the red rectangle in (a). c) Magnified image of the orange rectangle in b). d) Magnified image of the yellow rectangle in (c). e) EDS elemental mapping of the blue rectangle in (a). f) EDS spectra of the yellow points in (b). Atom labels correspond to K-line except for Br*, which is L-line. (TIF) [file pone.0272032.s005.tif]

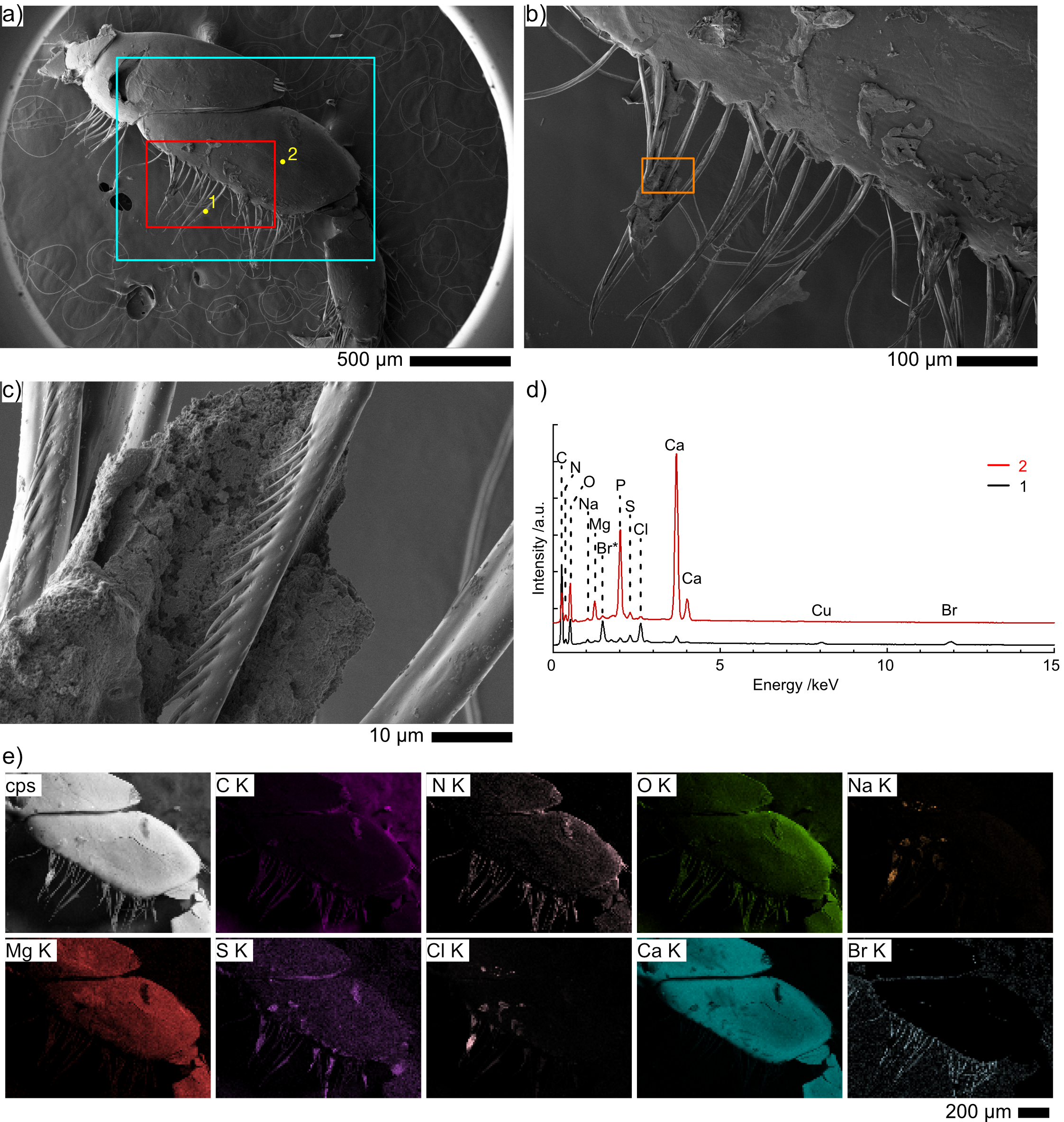

Supplement: S6 Fig — a) Low-magnification SEM image. b) Magnified image of the red rectangle in (a). c) Magnified image of the orange rectangle in b). Note that the solid between setae are unremoved sodium chloride, assigned based on EDS mapping in (e). d) EDS spectra of the yellow points in (b). e) EDS elemental mapping of the blue rectangle in (a). Atom labels correspond to K-line except for Br*, which is L-line. (TIF) [file pone.0272032.s006.tif]

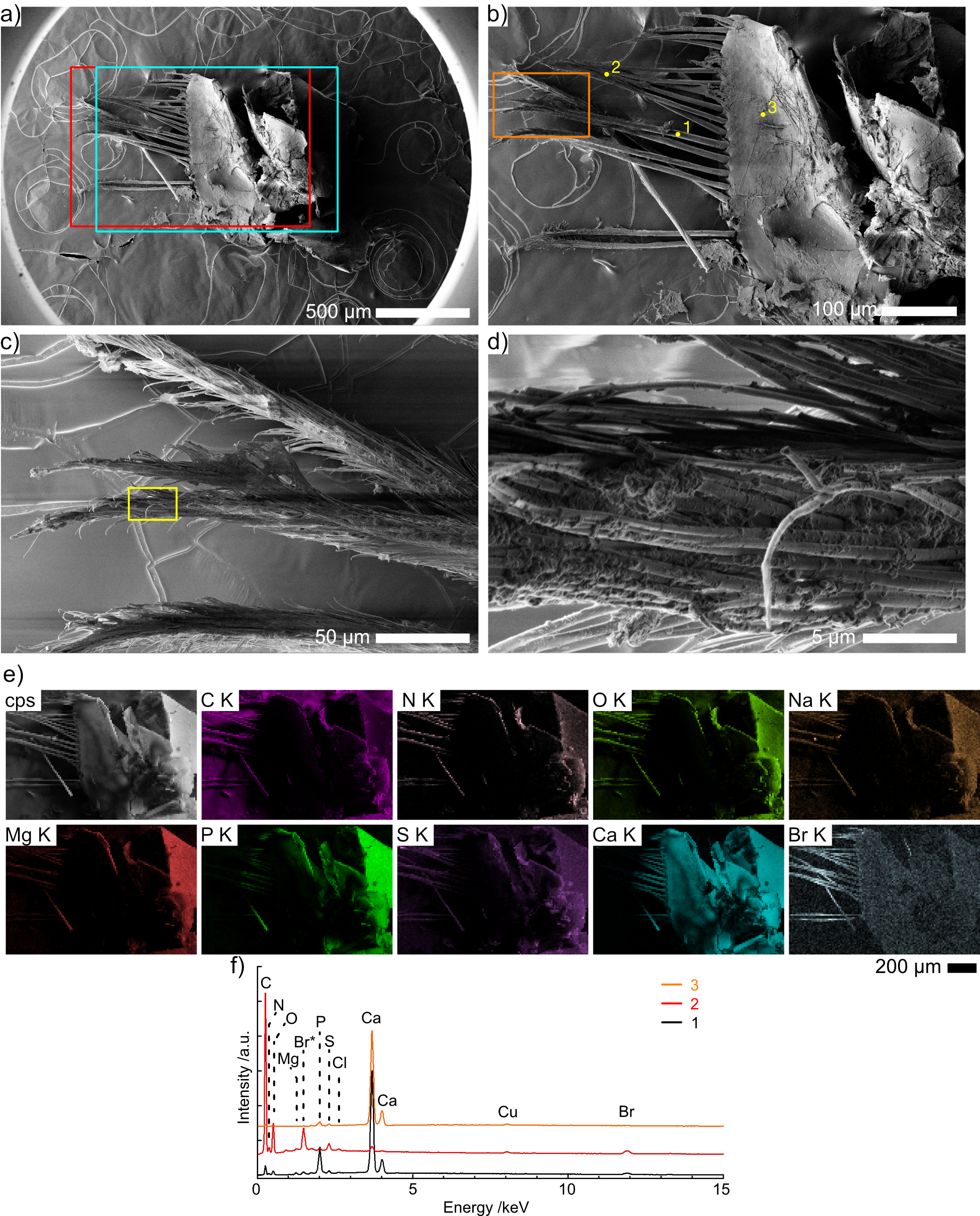

Supplement: S7 Fig — a) Low-magnification SEM image. b) Magnified image of the red rectangle in (a), c) Magnified image of the orange rectangle in b). d) Magnified image of the yellow rectangle in (c). e) EDS maps of the blue rectangle in (a). f) EDS spectra of the yellow points in (b). Atom labels correspond to K-line except for Br*, which is L-line. (TIF) [file pone.0272032.s007.tif]

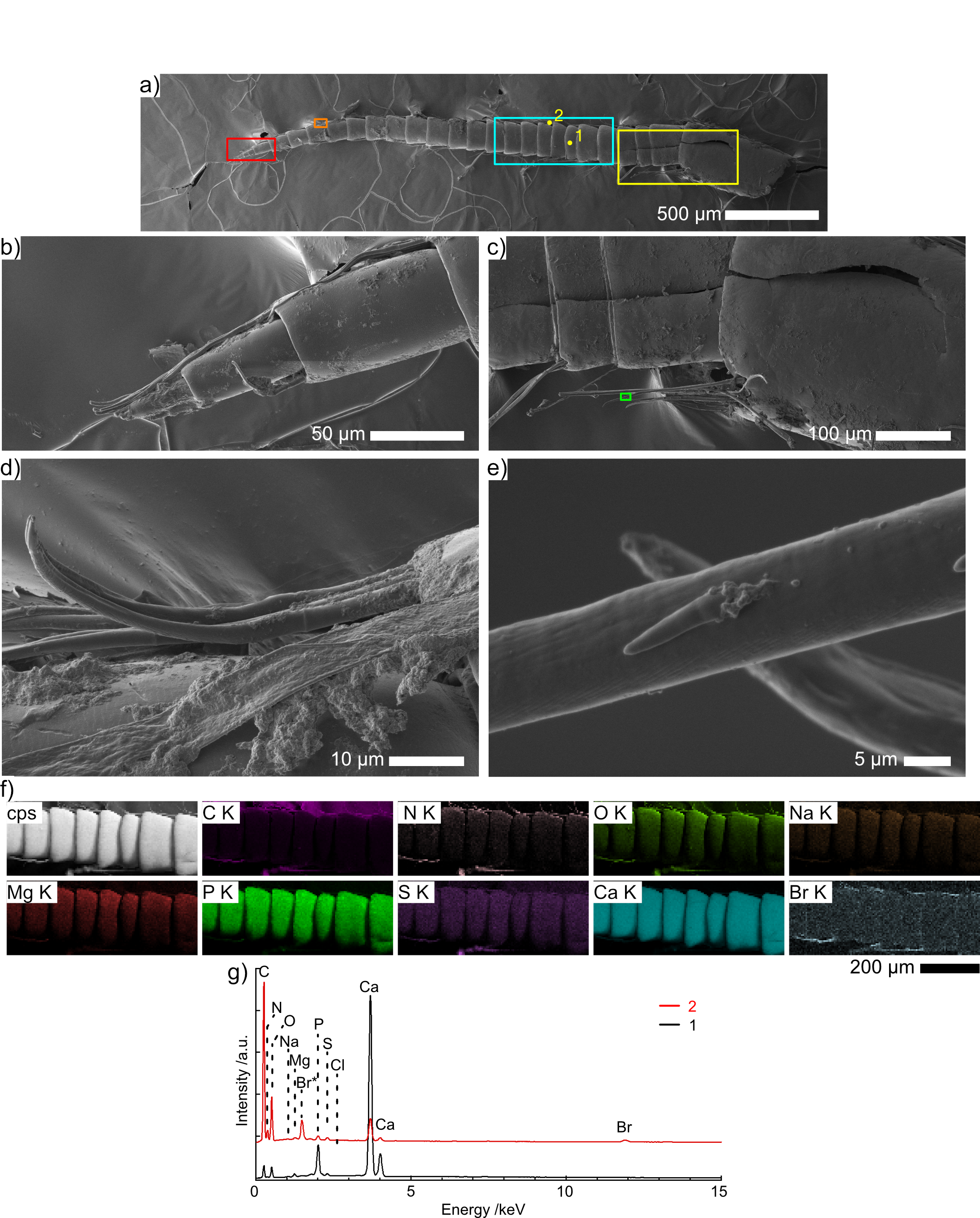

Supplement: S8 Fig — a) Low-magnification SEM image. b) Magnified image of the red rectangle in (a). c) Magnified image of setae from the article in the yellow rectangle in (b). d) Magnified image of the orange rectangle in (a). e) Magnified image of short barbs in the green rectangle in (c). f) EDS elemental mapping of the blue rectangle in (a). g) EDS spectra of the yellow points in (a). Atom labels correspond to K-line except for Br*, which is L-line. (TIF) [file pone.0272032.s008.tif]

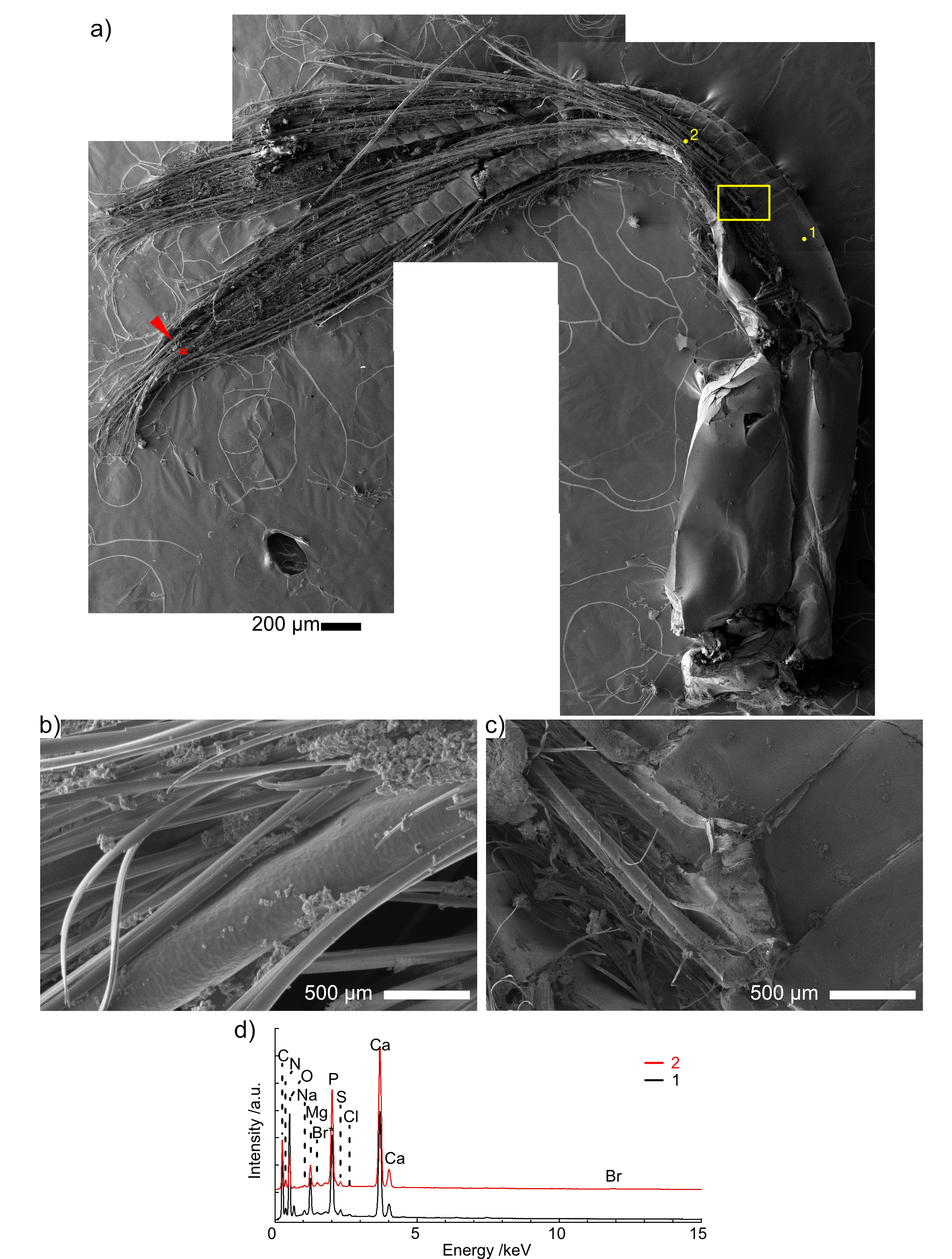

Supplement: S9 Fig — a) low-magnification SEM montage image. b) Magnified image of the red rectangle near the red triangle in (a) showing the two types of setae. c) Magnified image of the yellow rectangle in b). d) EDS spectra of the yellow points in (a). Atom labels correspond to K-line except for Br*, which is L-line. (TIF) [file pone.0272032.s009.tif]

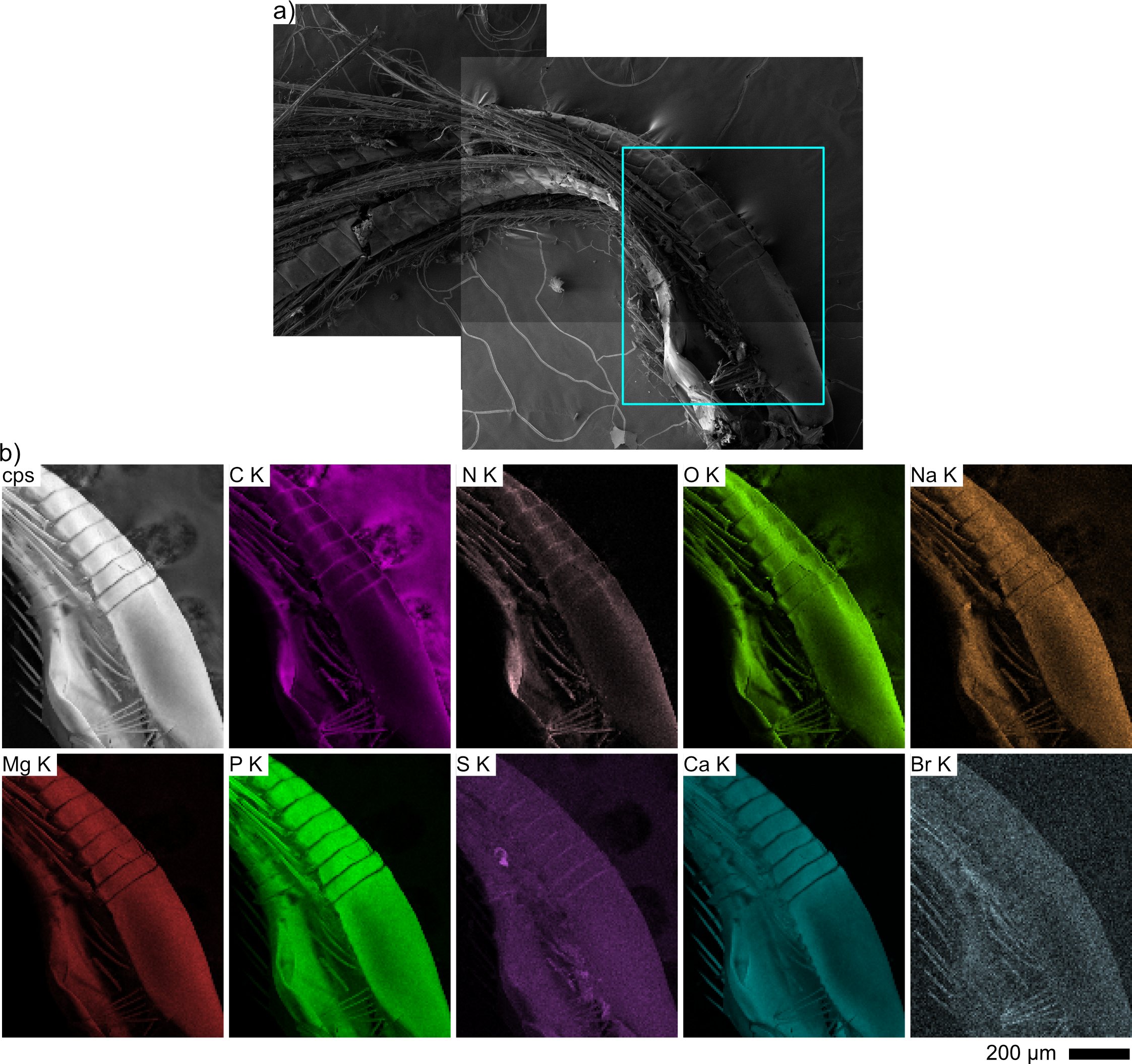

Supplement: S10 Fig — a) Low-magnification SEM montage image, which is a part of S8 Fig. b) EDS elemental mapping of the blue rectangle in (a). (TIF) [file pone.0272032.s010.tif]

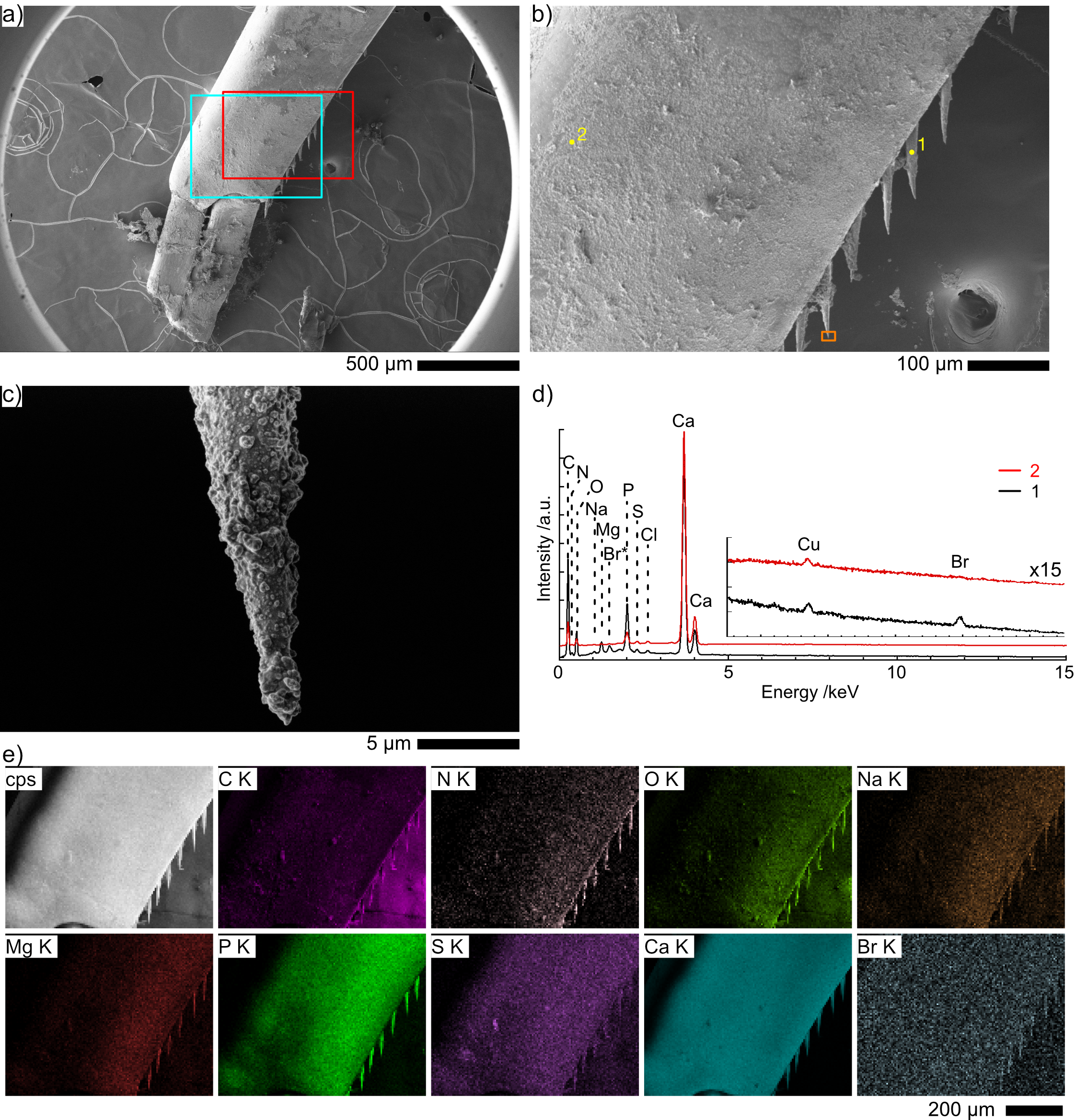

Supplement: S11 Fig — a) Low-magnification SEM image. b) Magnified image of the red rectangle, left middle in (a). c) Magnified image of the orange rectangle in b). d) EDS spectra of the yellow points in (b). e) EDS elemental mapping of the blue rectangle in (a). Atom labels correspond to K-line except for Br*, which is L-line. (TIF) [file pone.0272032.s011.tif]

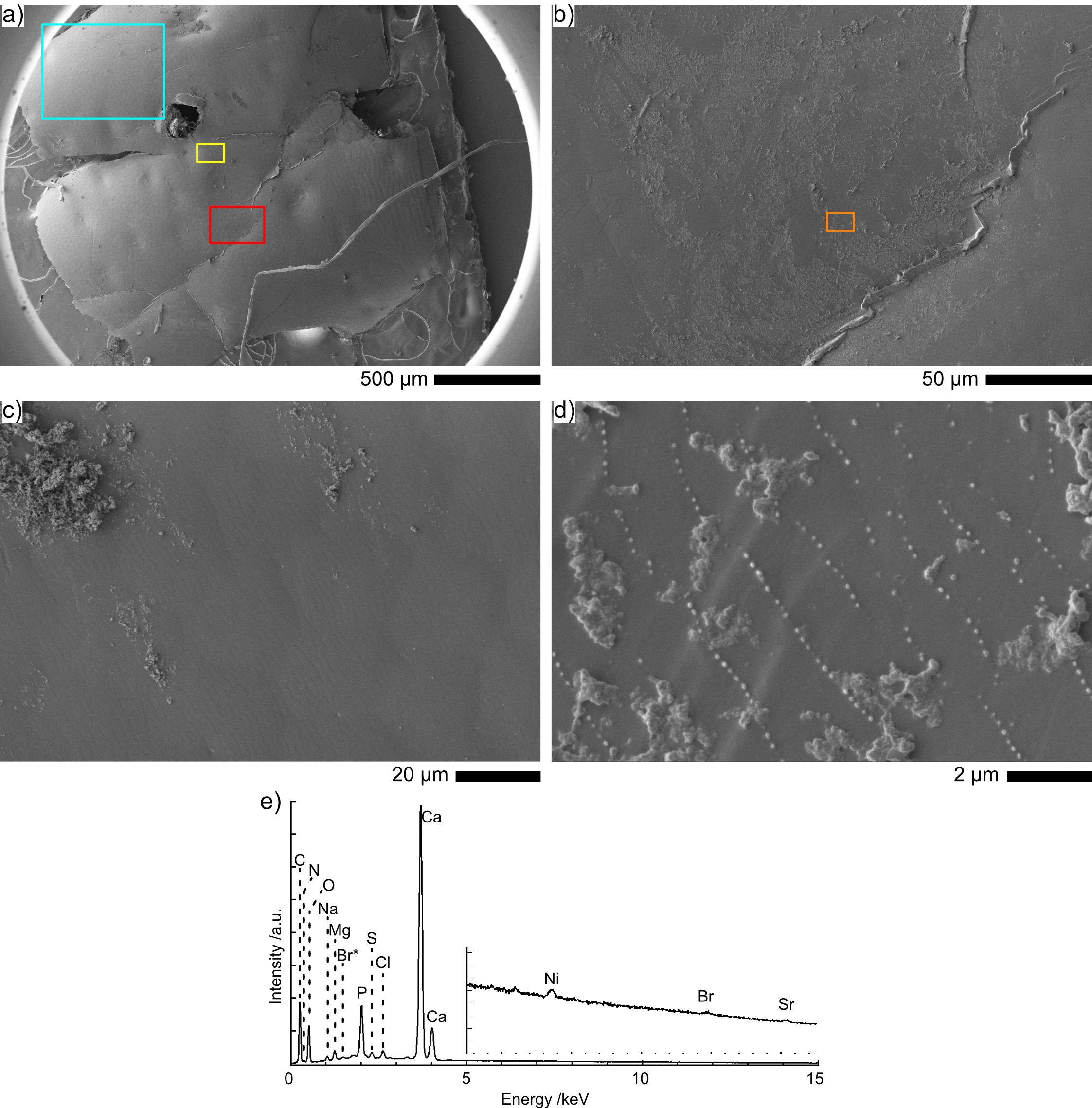

Supplement: S12 Fig — a) Low-magnification SEM image, b) Magnified image of the red rectangle, left middle in (a). c) Magnified image of the orange rectangle in (b) showing the hexagonal elements. d) Magnified image of (b) showing the bead-like structure. e) EDS sum spectra of (e). The surface was elementally homogeneous and the EDS maps are not shown. Atom labels correspond to K-line except for Br*, which is L-line. (TIF) [file pone.0272032.s012.tif]
