# Supplementary material for: Unusual bromine enrichment in the gastric mill and setae of the hadal amphipod Hirondellea gigas
Source: PLoS One. 2022 Aug 4;17(8):e0272032. doi: 10.1371/journal.pone.0272032 (PMC9352070; doi:10.1371/journal.pone.0272032)
Supplement: S2 Table — Top 10 values are shown in bold for visibility. (PDF) [file pone.0272032.s014.pdf]

S2 Table

	C	O	F	Mg	Al	Si	P	Cl	Ca	Br
C	1	-0.07	-0.02	-0.08	-0.01	-0.03	-0.09	-0.15	-0.04	0.07
O		1	**0.15**	**0.29**	0.06	0.05	**0.28**	-0.04	**0.70**	**0.11**
F			1.	**0.12**	0.02	0.03	0.08	-0.02	**0.19**	0.02
Mg				1	0.02	0.06	**0.24**	0.01	**0.37**	0.08
Al					1	-0.01	0.06	-0.02	0.09	0.08
Si						1	0.04	0.04	0.08	0.02
P							1	0.00	**0.35**	0.07
Cl								1	-0.07	0.01
Ca									1	0.15
Br										1
