# Supplementary material for: Unusual bromine enrichment in the gastric mill and setae of the hadal amphipod Hirondellea gigas
Source: PLoS One. 2022 Aug 4;17(8):e0272032. doi: 10.1371/journal.pone.0272032 (PMC9352070; doi:10.1371/journal.pone.0272032)
Supplement: S3 Table — GM, gastric mill; Mxp, maxillipet; Md, mandible; Mx2, maxilla 2; An, antenna; Pl, pleopod; Ur, uropod; Cx, coxal plate; Pe, pereonite. Values are reported by the atomic ratio ± errors in per cent. Note that the spectra were acquired under non-optimal condition, i.e. curved surface, and may contain large errors in the relative values. Area analysis was performed on Cx and Pe. (PDF) [file pone.0272032.s015.pdf]

S3 Table

| | GM 1 | GM 2 | GM 3 | Mxp 1 | Mxp 2 | Md 1 | Md 2 | Mx2 1 | Mx2 2 | Mx2 3 |
|---|---|---|---|---|---|---|---|---|---|---|
| C K | 43.07±3.75 | 52.87±3.71 | 58.08±3.65 | 57.00±5.02 | 27.35±2.44 | 32.31±3.13 | 13.99±1.55 | 60.32±4.12 | 62.32±3.50 | 28.26±2.50 |
| N K | 16.45±1.97 | 17.72±2.19 | 13.95±1.79 | 14.32±1.57 | 14.02±1.87 | 14.60±2.63 | <0.01 | 10.26±1.58 | 11.29±1.64 | 11.56±1.60 |
| O K | 21.47±2.33 | 25.89±2.71 | 24.20±2.50 | 17.13±1.75 | 39.19±3.93 | 21.76±2.46 | 14.08±1.98 | 25.57±2.66 | 25.27±2.62 | 37.93±3.80 |
| F K | 0.90±0.25 | 0.64±0.39 | 0.86±0.41 | 0.13±0.05 | 1.84±0.24 | 1.31±0.33 | 1.18±0.75 | <0.01 | <0.01 | 3.27±0.38 |
| Na K | 0.14±0.05 | 0.03±0.02 | 0.35±0.04 | 5.97±0.50 | 0.37±0.06 | <0.01 | <0.01 | 0.06±0.03 | 0.05±0.02 | 0.30±0.05 |
| Mg K | 0.66±0.07 | 0.21±0.03 | 0.51±0.04 | 0.35±0.03 | 2.18±0.18 | 0.59±0.07 | <0.01 | 0.17±0.02 | 0.08±0.01 | 2.59±0.21 |
| P K | 2.23±0.11 | 0.17±0.02 | 0.51±0.02 | 0.22±0.01 | 5.22±0.20 | 6.15±0.29 | 2.75±0.18 | 0.59±0.03 | 0.06±0.00 | 5.33±0.21 |
| S K | 1.07±0.05 | 0.29±0.02 | 0.53±0.01 | 0.40±0.01 | 0.35±0.03 | 0.53±0.05 | 0.72±0.06 | 0.18±0.01 | 0.13±0.01 | 0.44±0.03 |
| Cl K | 0.16±0.02 | 0.09±0.01 | 0.03±0.00 | 3.82±0.06 | 0.14±0.01 | 0.13±0.02 | 0.02±0.01 | 0.03±0.01 | 0.03±0.00 | 0.03±0.01 |
| K K | 0.04±0.01 | <0.01 | <0.01 | 0.05±0.01 | 0.02±0.01 | 0.07±0.02 | 0.30±0.05 | 0.02±0.01 | <0.01 | 0.02±0.01 |
| Ca K | 12.18±0.16 | 1.21±0.02 | 0.90±0.02 | 0.32±0.01 | 9.04±0.11 | 20.92±0.31 | 58.09±0.75 | 1.94±0.03 | 0.20±0.01 | 10.04±0.12 |
| Br K | 1.22±0.06 | 0.74±0.04 | <0.01 | 0.06±0.01 | 0.04±0.02 | 1.13±0.09 | 1.09±0.19 | 0.64±0.04 | 0.28±0.02 | 0.05±0.01 |

| | An 1 | An 2 | Pl 1 | Pl 2 | Ur 1 | Ur 2 | Cx | Pe |
|---|---|---|---|---|---|---|---|---|
| C K | 25.88±2.21 | 61.81±3.26 | 33.59±2.70 | 38.74±3.24 | 57.94±4.14 | 40.30±2.99 | 30.23±2.34 | 43.75±3.36 |
| N K | <0.01 | 11.60±1.63 | 7.60±1.00 | 15.31±1.67 | 6.27±0.81 | 5.31±2.76 | 8.00±1.21 | <0.01 |
| O K | 36.30±3.80 | 25.16±2.62 | 44.11±4.16 | 30.45±3.06 | 23.46±2.41 | 23.69±2.58 | 42.70±4.13 | 35.46±3.54 |
| F K | 1.01±0.18 | <0.01 | 4.85±0.53 | 1.28±0.17 | 0.36±0.08 | 0.62±0.13 | 2.13±0.25 | 0.52±0.08 |
| Na K | 0.25±0.06 | 0.22±0.03 | 0.73±0.08 | 0.26±0.04 | 0.34±0.04 | 0.27±0.05 | 0.94±0.09 | 0.87±0.09 |
| Mg K | 0.76±0.08 | 0.11±0.01 | 2.77±0.22 | 2.09±0.17 | 0.94±0.08 | 0.26±0.04 | 1.68±0.13 | 0.97±0.08 |
| P K | 4.72±0.20 | 0.12±0.01 | 2.80±0.11 | 4.78±0.17 | 2.14±0.07 | 1.63±0.08 | 3.35±0.13 | 2.70±0.10 |
| S K | 0.25±0.03 | 0.06±0.00 | 0.15±0.01 | 0.20±0.02 | 0.11±0.01 | 0.25±0.02 | 0.26±0.01 | 0.28±0.01 |
| Cl K | 0.02±0.01 | <0.01 | 0.03±0.00 | 0.04±0.01 | 0.08±0.01 | 0.21±0.02 | 0.03±0.00 | 0.33±0.01 |
| K K | 0.04±0.01 | <0.01 | <0.01 | 0.03±0.01 | <0.01 | 0.09±0.01 | <0.01 | 0.06±0.01 |
| Ca K | 24.99±0.26 | 0.67±0.01 | 3.22±0.03 | 6.56±0.07 | 8.17±0.08 | 27.03±0.22 | 10.44±0.10 | 11.95±0.11 |
| Br K | 0.05±0.03 | 0.17±0.01 | 0.02±0.01 | 0.07±0.01 | 0.10±0.02 | 0.11±0.04 | 0.04±0.00 | 0.03±0.01 |
