# Supplementary material for: Unusual bromine enrichment in the gastric mill and setae of the hadal amphipod Hirondellea gigas
Source: PLoS One. 2022 Aug 4;17(8):e0272032. doi: 10.1371/journal.pone.0272032 (PMC9352070; doi:10.1371/journal.pone.0272032)
Supplement: Attachment — Submitted filename: PONE-D-22-02007_MS reviewEDITS.pdf [file pone.0272032.s016.pdf]

PLOS ONE

Unusual bromine enrichment in the gastric mill and setae of the hadal amphipod Hirondellea gigas

--Manuscript Draft--

**Manuscript Number:**	PONE-D-22-02007
**Article Type:**	Research Article
**Full Title:**	Unusual bromine enrichment in the gastric mill and setae of the hadal amphipod Hirondellea gigas
**Short Title:**	Bromine enrichment in the gastric mill and setae of Hirondellea gigas
**Corresponding Author:**	Satoshi Okada<br>Japan Agency for Marine-Earth Science and Technology: Kaiyo Kenkyu Kaihatsu Kiko<br>Yokosuka, Kanagawa JAPAN
**Keywords:**	amphipod; Hirondellea gigas; scanning electron microscopy; artefact; bromine; carbonate compensation depth; gastric mill
**Abstract:**	The hadal amphipod Hirondellea gigas is an emblematic animal of the Pacific trenches, and has a number of special adaptations to thrive in this 'extreme' environment including the deepest part of the Earth's ocean. One such adaptation that has been suggested is the presence of an 'aluminum gel shield' on the surface of its body in order to prevent the dissolution of calcitic exoskeleton below the carbonate compensation depth. However, this has not been investigated under experimental conditions that sufficiently prevents aluminum artefacts, and the possibility of other elements with similar characteristic X-ray energy as aluminum (such as bromine) has not been considered. Here, we show with new electron microscopy data carried under optimized conditions to minimize aluminum artefacts that H. gigas actually does not have an aluminum shield – instead many parts of its body are enriched in bromine, particularly gastric ossicles and setae. Results from elemental analyses pointed to the use of calcite partially substituted with magnesium by H. gigas in its exoskeleton, in order to suppress dissolution. Our results exemplify the necessity of careful sample preparation and analysis of the signals in EDS analysis, and the importance to analyze at different electron energies in microscopic 'elemental anatomy'.
**Order of Authors:**	Satoshi Okada
	Chong Chen
	Hiromi Kayama Watanabe
	Noriyuki Isobe
	Ken Takai
**Additional Information:**	
**Question**	**Response**
**Financial Disclosure**<br><br>Enter a financial disclosure statement that describes the sources of funding for the work included in this submission. Review the submission guidelines for detailed requirements. View published research articles from *PLOS ONE* for specific examples.<br><br>This statement is required for submission and **will appear in the published article** if the submission is accepted. Please make	S.O., [Grant-in-Aid for Young Scientists Grant Number JP19K15379], Japan Society for the Promotion of Science (JSPS), https://www.jsps.go.jp/; C.C. and H.K.W., [Grant-in-Aid for Scientific Research Grant Number 18K06401], JSPS. The funders had no role in study design, data collection and analysis, decision to publish, or preparation of the manuscript.

Powered by Editorial Manager® and ProduXion Manager® from Aries Systems Corporation

sure it is accurate.<br><br>**Unfunded studies**<br>Enter: *The author(s) received no specific funding for this work.*<br><br>**Funded studies**<br>Enter a statement with the following details:<br>• Initials of the authors who received each award<br>• Grant numbers awarded to each author<br>• The full name of each funder<br>• URL of each funder website<br>• Did the sponsors or funders play any role in the study design, data collection and analysis, decision to publish, or preparation of the manuscript?<br>• **NO** - Include this sentence at the end of your statement: *The funders had no role in study design, data collection and analysis, decision to publish, or preparation of the manuscript.*<br>• **YES** - Specify the role(s) played.<br><br>* typeset	
**Competing Interests**<br><br>Use the instructions below to enter a competing interest statement for this submission. On behalf of all authors, disclose any competing interests that could be perceived to bias this work—acknowledging all financial support and any other relevant financial or non-financial competing interests.<br><br>This statement is required for submission and **will appear in the published article** if the submission is accepted. Please make sure it is accurate and that any funding sources listed in your Funding Information later in the submission form are also declared in your Financial Disclosure statement.<br><br>View published research articles from *PLOS ONE* for specific examples.	The authors have declared that no competing interests exist.

Powered by Editorial Manager® and ProduXion Manager® from Aries Systems Corporation

**NO authors have competing interests**<br><br>Enter: *The authors have declared that no competing interests exist.*<br><br>**Authors with competing interests**<br><br>Enter competing interest details beginning with this statement:<br><br>*I have read the journal's policy and the authors of this manuscript have the following competing interests: [insert competing interests here]*<br><br>* typeset	
**Ethics Statement**<br><br>Enter an ethics statement for this submission. This statement is required if the study involved:<br><br>• Human participants<br>• Human specimens or tissue<br>• Vertebrate animals or cephalopods<br>• Vertebrate embryos or tissues<br>• Field research<br><br>Write "N/A" if the submission does not require an ethics statement.<br><br>General guidance is provided below. Consult the submission guidelines for detailed instructions. **Make sure that all information entered here is included in the Methods section of the manuscript.**	N/A

Powered by Editorial Manager® and ProduXion Manager® from Aries Systems Corporation

**Format for specific study types**<br><br>**Human Subject Research (involving human participants and/or tissue)**<br>• Give the name of the institutional review board or ethics committee that approved the study<br>• Include the approval number and/or a statement indicating approval of this research<br>• Indicate the form of consent obtained (written/oral) or the reason that consent was not obtained (e.g. the data were analyzed anonymously)<br><br>**Animal Research (involving vertebrate animals, embryos or tissues)**<br>• Provide the name of the Institutional Animal Care and Use Committee (IACUC) or other relevant ethics board that reviewed the study protocol, and indicate whether they approved this research or granted a formal waiver of ethical approval<br>• Include an approval number if one was obtained<br>• If the study involved *non-human primates*, add *additional details* about animal welfare and steps taken to ameliorate suffering<br>• If anesthesia, euthanasia, or any kind of animal sacrifice is part of the study, include briefly which substances and/or methods were applied<br><br>**Field Research**<br><br>Include the following details if this study involves the collection of plant, animal, or other materials from a natural setting:<br>• Field permit number<br>• Name of the institution or relevant body that granted permission	
**Data Availability**<br><br>Authors are required to make all data underlying the findings described fully available, without restriction, and from the time of publication. PLOS allows rare exceptions to address legal and ethical concerns. See the PLOS Data Policy and FAQ for detailed information.	Yes - all data are fully available without restriction

A Data Availability Statement describing where the data can be found is required at submission. Your answers to this question constitute the Data Availability Statement and **will be published in the article**, if accepted.<br><br>**Important:** Stating 'data available on request from the author' is not sufficient. If your data are only available upon request, select 'No' for the first question and explain your exceptional situation in the text box.<br><br>Do the authors confirm that all data underlying the findings described in their manuscript are fully available without restriction?	
**Describe where the data may be found in full sentences. If you are copying our sample text, replace any instances of XXX with the appropriate details.**<br><br>• If the data are **held or will be held in a public repository**, include URLs, accession numbers or DOIs. If this information will only be available after acceptance, indicate this by ticking the box below. For example: *All XXX files are available from the XXX database (accession number(s) XXX, XXX.*).<br>• If the data are all contained **within the manuscript and/or Supporting Information files**, enter the following: *All relevant data are within the manuscript and its Supporting Information files.*<br>• If neither of these applies but you are able to provide **details of access elsewhere**, with or without limitations, please do so. For example:<br><br>*Data cannot be shared publicly because of [XXX]. Data are available from the XXX Institutional Data Access / Ethics Committee (contact via XXX) for researchers who meet the criteria for access to confidential data.*<br><br>*The data underlying the results presented in the study are available from (include the name of the third party*	All relevant data are within the manuscript and its Supporting Information files.

and contact information or URL).<br>• This text is appropriate if the data are owned by a third party and authors do not have permission to share the data.<br><br>* typeset	
Additional data availability information:	

Powered by Editorial Manager® and ProduXion Manager® from Aries Systems Corporation

Manuscript

Click here to access/download;Manuscript;okada_manuscript.docx

Unusual bromine enrichment in the gastric mill and setae of the hadal amphipod *Hirondellea gigas*

Satoshi Okada[1], Chong Chen[1], Hiromi Kayama Watanabe[1], Noriyuki Isobe[2], Ken Takai[1]

[1] Institute for Extra-cutting-edge Science and Technology Avant-garde Research (X-STAR), Japan Agency for Marine-Earth Science and Technology (JAMSTEC), 2-15 Natsushima-cho, Yokosuka, Kanagawa 237-0061, Japan

[2] Biogeochemistry Research Center, Research Institute for Marine Resources Utilization (MRU), Japan Agency for Marine-Earth Science and Technology (JAMSTEC), 2-15 Natsushima-cho, Yokosuka, Kanagawa 237-0061, Japan

Abstract (abstract 150-200 words)

The hadal amphipod *Hirondellea gigas* is an emblematic animal of the Pacific trenches, and has a number of special adaptations to thrive in this 'extreme' environmen[illegible] including the deepest part of the Earth's ocean. One such adaptation that has been suggested is the presence of an 'aluminum gel shield' on the surface of its body in order to prevent the dissolution of calcitic exoskeleton below the carbonate compensation depth. However, this has not been investigated under experimental conditions that sufficiently prevents aluminum artefacts, and the possibility of other elements with similar characteristic X-ray energy as aluminum (such as bromine) has not been considered. Here, we show with new electron microscopy data carried under optimized conditions to minimize aluminum artefacts that *H. gigas* actually does not have an aluminum shield – instead many parts of its body are enriched in bromine, particularly gastric ossicles and setae. Results from elemental analyses pointed to the use of calcite partially substituted with magnesium by *H. gigas* in its exoskeleton, in order to suppress dissolution. Our results exemplify the necessity of careful

sample preparation and analysis of the signals in EDS analysis, and the importance to analyze at different electron energies in microscopic 'elemental anatomy'.

Main text

Many animal groups have evolved hard parts for skeletal support (e.g. vertebrate bones), protection (e.g. molluscan shells), and feeding (e.g. mouth parts and teeth), among other functions – and often for multiple purposes. These hard parts are composed of either inorganic minerals such as calcite, aragonite, and hydroxyapatite, or organic polymers such as chitin, or a mixture of both. Inorganic biomineralized parts exposed to the outside world are often coated with organic matrices such as molluscan shells covered by a layer of periostracum made out of a combination of chitin and proteins.[1] In some cases, heavy transition metals and halogens are utilized to coat the mineralized tissues.[2,3] These external layers often play roles in the secretion of the biominerals, and they can also reinforce the structure and prevent the deterioration of the underlying mineral layer. For example, bivalves living deeper than the carbonate compensation depth (CCD; typically ~4500 m) where calcium carbonate dissolves quickly, use their periostracum to buffer against the natural shell dissolution.[4] Crustaceans are among the most successful invertebrates in marine environments and use hard parts for both protection in the form of calcified exoskeletons and feeding in the form of gastric mills. However, coatings of organic matrices in crustacean hard parts are not known with the sole exception of barnacle cuticular and epicuticular layers covering the plates.[5]

The hadal amphipod *Hirondellea gigas* is one of the most numerically dominant crustacean and megafauna at the deepest parts of the ocean, including the Challenger Deep in the Mariana Trench (10,994 m).[6] As special adaptations to the extreme hadal environment, this species possesses unique cellulase and is alleged to digest polysaccharides from sunken plant matter using a unique cellulase,[6,7] and perhaps most strikingly, is said to secrete

aluminum outside its exoskeleton to prevent the dissolution of calcium-based minerals, dubbed the 'aluminum shield'.[8] The aluminum shield was expected to be in a form of gel, supported by combination of energy-dispersive X-ray analysis (EDS) under scanning electron microscopy (SEM), and aluminum extraction experiments from the sediment of Challenger Deep. However, the results presented by Kobayashi et al.[8] was inconclusive and ambiguous due to the localization pattern of aluminum, where the signal intensity of aluminum was especially high only at the edge of the samples close to the aluminum stubs most prone to artefacts. Furthermore, conductive coatings were not performed for imaging, despite observation being undertaken in an aluminum-rich environment that is the SEM chamber. In addition, EDS spectra under scanning transmission electron microscope (STEM) of the exoskeleton did not provide enough information to support the existence of aluminum and its detailed nanostructure.

In order to confirm the presence of the 'aluminum shield' of *H. gigas* and to further substantiate its distribution and potential function, we designed a more careful experimental design to exclude aluminum artefacts. There are two possibilities where aluminum artefacts may be observed: 1) backscattered electron (BSE) from the observed specimen hitting the chamber made of aluminum alloy to generate secondary electrons (called SE3) as well as aluminum X-ray,[9]) overlapping of other elements that has similar characteristic X-ray energy to that of aluminum (Al K-line, 1.486 keV) and inseparable using conventional EDS whose energy resolution being ~130 eV. These elements include bromine (Br L-line, 1.480 keV), thulium (Tm M-line, 1.462 keV), and ytterbium (Yb M-line, 1.521 keV),[10] the latter two being unused in animals.[11,12] Our detailed analysis revealed the absence of aluminum and instead the presence of bromine in *H. gigas*. We serendipitously also discovered a unusual local enrichment of bromine on the surface of the sclerotized spines of its gastric mill components, including clear accumulation on the spines. Although bromine is a common element in seawater,[13] it is rarely preferentially secreted in the hard parts of animals, except in some cases where bromine with transition metals are utilized to

harden the hard parts.[2,3,14] Here, we report our findings from electron microscopic observations of *H. gigas*, with a focus on its bromine accumulation.

Methods

Materials

All the reagents were purchased either from Nacalai tesque Inc., Fuji Film Wako Pure Chemical Co., or Kanto Chemical Co., Inc. and used as received. Deionized water was supplied from Merck Milli-Q Integral 5.

Amphipod samples

Hirondellea gigas individuals were collected using a bait trap attached to a Free Fall Camera System at Izu-Ogasawara Trench near the Boso Triple Junction, (34°20.4296′N 142°0.6006′E), depth 9218 m on September 4, 2019 on R/V Yokosuka cruise YK19-11. The samples were immediately immersed in slush nitrogen (ca. –210 °C) for more than a minute to rapidly freeze the exoskeleton and stored at –80 °C during the cruise, and transferred to liquid nitrogen storage (actual storage temperature <–160 °C) after cruise.

Electron microscope instruments

SEM and scanning transmission electron microscopic (STEM) imaging were performed on a Helios G4 UX (Thermo Fisher Scientific), an Octane Elite Super energy dispersive X-ray analysis (EDS) detector (Octane Elite Super, AMETEK) and a cryogenic stage with preparation chamber (PP3010T, Quorum). Transmission electron microscopy (TEM) was performed on a Tecnai G2 20 (Thermo Fisher Scientific, operated at 200 kV) equipped with a bottom-mounted 2k x 2k Eagle Charge-Coupled Device camera (Eagle, Thermo Fisher Scientific) and RTEM-S 61700NE an EDS detector (RTEM-S 61700NE, AMETEK). Electron probe microanalysis (EPMA) was performed on a JXA-8500 (JEOL Ltd.) equipped with five wave-length dispersive X-ray detectors (WDS, XM-86030 and XM-86010, JEOL Ltd.).

Sample preparation and semi-thin section imaging

~~The~~ frozen amphipod ~~was~~ dehydrated in acetone at –80 °C for three days, and gradually warmed to room temperature. The dehydrated amphipod was replaced with QY-1 (Ohkenshoji Inc.), QY-1/epoxy mixture, then with an epoxy resin (TAAB), trimmed into pieces using laser blades, and embedded in the epoxy resin following the Luft's method. [15] Transverse sections of the head part ~~was~~ taken from ~~the~~ anterior to~~wards the~~ posterior into 1 µm-thick semi-thin sections using an ultramicrotome (Ultracut S or EM UC7, Leica) with a diamond knife (45°, Diatome)~~, the~~ sections were collected on a glass slide covered with Kapton tape (Nitto Denko Co., P-224, 64 µm thickness) using a nickel loop. The slide was coated with carbon for conductivity using CADE (Meiwaforsis Inc.).

Semi-thin sections were subjected to SEM/EDS analysis operated at 1 kV for morphological imaging and at 10 kV for elemental analysis and mapping. Chlorine was observed uniformly in the epoxy resin, and carbon coating ~~were~~ performed for conductivity on carbon-based substrate~~, and~~ the localization of these two elements were not discussed in detail.

Elemental analysis at high energy resolution was performed on EPMA operated at 20 kV and beam current of 12 nA. The elemental colocalization in the EPMA map was quantified using cross correlation image analysis.[16] The cross-correlation factor γ between two elemental maps *a* and *b* was calculated as

$$\gamma(a,b) = \frac{\sum_{ij}\{[I_a(r_{ij}) - \bar{I}_a] \cdot [I_b(r_{ij}) - \bar{I}_b]\}}{\sqrt{\sum_{ij}[I_a(r_{ij}) - \bar{I}_a]^2} \cdot \sqrt{\sum_{ij}[I_b(r_{ij}) - \bar{I}_b]^2}} \quad (1)$$

where $I_x(r_{ij})$ denotes the X-ray intensity of map x at the pixel position (i, j), and $\bar{I}_x$ is the averaged intensity of map x over the image. The cross-correlation calculation and EPMA elemental mapping were implemented in Python 3.9.1.

Microscopic observation and image analysis of a dissected amphipod

Another frozen individual of *H. gigas* (total length 26.0 mm, telson length 5.3 mm) was defrosted and dissected for three-dimensional structural investigation under a stereomicroscope (SZX7, Olympus) and photos were taken using an Olympus E-M5 Mark III DSLR camera. The amphipod was dissected into different parts, with observations focusing on: mandible, maxilla, maxilliped, antennae, gastric mill (pterocardiac ossicles in the cardiac stomach), pereonites, pleopod, uropod, coxal plates. Dissected samples were placed within Eppendorf tubes (0.2 mL), dehydrated by an increasing series of ethanol (50, 60, 70, 70, 70, 80, 90, 99, 100, 100, 100%, more than 10 minutes for each step), then substituted with *tert*-butanol, frozen at –20 °C, and dried under vacuum (<1 x 10^{-1} Pa) to obtain dried specimens, which were then mounted on a Kapton tape on a glass slide using a double-sided carbon tape. The mounted samples were coated with carbon for conductivity, and analyzed in SEM/EDS operated at 1 kV for morphological imaging and 20 kV for EDS analyses.

Precautions to avoiding artificial aluminum signals

To observe the potential aluminum layer in the exoskeleton of *H. gigas*, several precautions were taken to eliminate potential artificial X-ray signals from aluminum sources, which is a very common element and contaminant in electron microscopy.[11] A glass slide covered with carbon-coated Kapton tape was selected as an aluminum-free substrate for SEM imaging. The use of Kapton/carbon reduces emission of backscattered electron (BSE) from the substrate, which may generate secondary electrons (SE3) and X-ray by hitting the SEM chamber made of aluminum alloy.[9] The amphipod tissue was mounted as carbon-coated flat semi-thin sections (unlike the bulk specimen in [8]) due to three benefits. Firstly, the spatial resolution of semi-thin section is higher than that of using a bulk specimen where the irradiated electron beam would scatter within the sample and generates X-ray from the interior and surrounding positions of the irradiated position, resulting in decreased spatial resolution and make it undecisive whether the signal actually originated directly from the

observed surface – the use of semi-thin sections can avoid this issue. Secondly, the charge-up of the thin section is minimized, as primary electrons injected to the thin section are not retained in the sample but instead penetrate through to the underlying layer, where they are neutralized by conductive coatings.[17] In the case of bulk samples with insulative interior, as is in such cases as observation of biological materials, accumulated charges within the sample bends the electron trajectory, resulting in decreased spatial resolution. Finally, using semi-thin sections ensure the electrons and X-rays are emitted homogeneously from a flat surface, which is desirable for quantitative analysis. Surface roughness causes abnormal contrast such as edge effect to brighten edges, and shading of X-rays to decrease signal intensities of low-energy X-ray.[18] Without these precautions, total X-ray counts from each position does not necessarily reflect the sample's true elemental composition. We used the counts per pixel (cps) images, showing total X-ray counts from each pixel, as the criteria to distinguish whether the signal originated from the sample or from the background (bremsstrahlung)

The condition to observe SEM/EDS maps were set either to 10 kV or to 30 kV. The former condition was used to enhance spatial resolution and signal intensities of light elements, and the latter to observe high-energy characteristic X-rays from possible heavy elements.

X-ray measurement

Dried pereonites were cut into flat pieces and mounted on an X-ray diffractometer X'Pert PRO (PANalytical) with with a Cu radiation source (Kα = 1.5418 Å) at 45 kV and 40 mA. The diffractogram from 10° to 60°was acquired at 0.0167° step at the scan speed of 0.07°/s. The 16 spectra were integrated, and mineral assignment was performed on HighScore 3.0.5 (PANalytical).

Results

Elemental distribution and composition of *H. gigas*

Following the ~~precautional~~ methods for the EDS measurement to avoid artificial aluminum signals, thin sections of *Hirondellea gigas* were subjected to SEM/EDS analyses (Fig 1, Fig S1). EDS maps of the pereonite and telson showed no evidence of aluminum, while strong signals of carbon, oxygen, phosphorous, and calcium as well as weak signal of sulfur were observed in the area where bright contrast was observed in the SEM images (Fig 1a). This result agrees with the previous observation[8] that the exoskeleton is composed of calcium carbonate and proteinaceous organic materials. Other elements such as sodium, aluminum, and silicon were within the background levels of bremsstrahlung X-ray. The EDS maps showed uniform existence of these elements, indicating the compositional homogeneity of the exoskeleton. Soft parts within the exoskeleton were composed of organic materials, and the signal of chlorine corresponds to the epoxy resin. The intensities of the aluminum-like signal (as aluminum overlaps with that of bromine, thulium, and ytterbium) was similar to that of the background signals, indicating that this signal was below the detection limit of the imaging condition used here.

To confirm that the mineral components of the pereonites of *H. gigas* is the same as previously observed (i.e. mainly calcitic),[8] XRD measurement ~~was~~ performed using the dissected dried pereonite. Nine ~~among~~ ten peaks were assigned to ~~that of~~ calcite (Fig. S2). The residual peaks at 19.7° and 26.7° were explained by α-chitin, which is ubiquitous in crustacean exoskeletons.[19,20] This result combined with EDS analysis confirmed that the exoskeleton of *H. gigas* is mainly made of calcite and organic matrices, including phosphorous.

The elemental composition of the semi-thin sections through the gastric mill was different from that of pereonite (Fig. 1b,c). The tissues surrounding the gastric mill showed similar elemental content to that of the pereonite. On the other hand, a strikingly different composition was found on the gastric mill itself (Fig. 1c)[21] which appeared to form a bilayered structure with an inner layer composed of carbon, nitrogen, oxygen, sodium,

magnesium, and phosphorous, while the outer layer was composed of carbon, nitrogen, oxygen, sodium, calcium, and aluminum-like signal at 1.48 keV (Fig 1). Magnified EDS maps showed the thickness of the outer layer to be ~1 μm.

EPMA analysis was also performed using similar sections of *H. gigas* to correctly assign the X-ray signal at 1.48 keV (Fig. 2). Qualitative WDS spectrum of outer layer of the gastric mill showed two peaks around 1.48 keV: 1.481 and 1.527 keV. Aluminum and bromine are the two possible candidates of these peaks: L-line of bromine appears at 1.293 and 1.480 kV while K-line of aluminum appears at 1.486 keV[10]. Another signal at 11.92 keV was also observed, corresponding to the K-line of bromine, confirming that the aluminum-like signals we have observed is in fact bromine. The intensity around 1.48 keV can thus be explained without aluminum. EPMA maps showed directional bromine localization toward the side exposed to seawater, similar to that observed in the EDS maps.

The elemental colocalization was calculated by cross correlation analysis of the whole image (Table S1).[16] High positive cross correlation values are obtained when two elements have similar relative intensity in any position, i.e. colocalized, and becomes zero when two random noise images were correlated. Note that correlation with carbon is not reliable, as the surrounding resin and the Kapton tape below the sample contain large amount of carbon which overshadows the carbon in the cross-sectioned tissues. High correlation was observed between Ca-O-F-Mg-P, correlation values ranging from 0.29 to 0.70. On the other hand, the correlation values of bromine against calcium-colocalizing elements was less than 0.1, indicating that the bromine layer is separated from the calcium-based minerals. Quantitative X-ray analysis showed the absence of calcium on bromine-rich outer layer (Fig. S3). Low concentration of bromine and coexistence of fluorine and magnesium were also supported by the quantitative analysis (Fig. S3).

Similarly, EPMA maps of the pereonite were taken to explore elements of the exoskeleton (Fig. 3, Table S2). Cross correlation values indicated the high colocalization of Ca-O-Mg-P ~~by the~~ correlation values ranging from 0.15 to 0.70. The correlation of calcium-

colocalizing minerals were small due to low X-ray counts. Correlation of bromine to calcium-colocalizing minerals were less than 0.1 except for oxygen, denying strong bromine colocalization. Quantitative analysis clearly showed the absence of either bromine or aluminum (Fig. S3).

EDS investigation of the dissected specimen

To further explore the localization of bromine in *Hirondellea gigas*, a dissected individual was freeze-dried and observed under SEM/EDS. EDS maps showed that the hard tissues in the *H. gigas* is mainly composed of carbon, nitrogen, oxygen, phosphorous, and calcium as major elements, and fluorine, sodium, magnesium, chlorine, and bromine as minor elements.

The gastric mill exhibited several large sclerotized spines (Fig. 4, Fig. S4). The dimension of the spines was found to be typically 100 μm in length and 15–25 μm in diameter (Fig. 4a, b). Fine filter setae around the sclerotized spines were simple and covered by adsorbents, presumably mucus (Fig. 4c). Elemental map of Br K indicated accumulation of bromine on the spines (Fig. 4d, Table S3). Line profile analysis of the Br K line showed that the bromine intensity was 12–13 at the root and 15 at the tip, indicating preferential bromine accumulation at the tip (Fig. 4e). The presence of weak calcium signals, combined with EPMA results above, point to a bromine-rich and calcium-poor organic layer coating a calcium-rich inorganic layer. Although the intensity was lower, bromine accumulation was also observed on the filter setae. The pterocardiac ossicle itself at the base of the spines are made of carbon, oxygen, magnesium, phosphorous, and calcium, while bromine was not observed with significant concentration.

Bromine accumulation was also observed on the setae of mouth parts, specifically the robust setae (Fig. 5, Fig. S5–S7). The clearest accumulation was seen on maxilla 2 (Fig. 5, Fig. S5). The maxilla itself was mainly composed of calcium carbonate together with magnesium and phosphorous, and the bristles were found on the surface, where they are

exposed to seawater. Three types of setae were present on ~~the~~ maxilla 2 (Fig. 5a,b). One was simple robust setae with a diameter of 10–20 μm and a length of 150–350 μm, occurring on the distal part of ~~the~~ maxilla 2; a second type of robust setae was similar in size but plumose with many fine setae (diameter of 0.6–2.5 μm and length of 35–50 μm); a third type was fine, setulose setae 0.5–1.5 μm in diameter with a length of 50–150 μm. EDS maps showed that bromine is accumulated on both types of robust setae with carbon, nitrogen, oxygen, and sulfur, but not on the finer, setulose setae which was rich in phosphorous and calcium (Fig. 5c, Table S3). Similarly, selective bromine accumulation on the robust setae was found in both the maxilliped and the mandible (Fig. S6–7, Table S3)

Microscopic observation of the antenna (Fig. 6, Fig. S8) also revealed the presence of bromine specifically on the setae extending from the distal parts of each article. These setae were simple and fine, 50–80 μm in length and 1–3 μm in diameter (Fig. 6a, b). Setae on the distal end of article 1 of the antennae are, however, barbed and have short barbs projecting out laterally. Elemental maps and point analysis revealed bromine accumulation on the setae, free of magnesium, phosphorous, and calcium that were found on the articles themselves (Fig. 6c, Table S3). The setae were characterized by high carbon, nitrogen, oxygen, and sulfur contents, while magnesium and phosphorous were less significant.

Observations of the pleopods revealed bromine accumulation on robust setae as well as the outer surface of the pleopods themselves (Fig. 7, Fig. S9–S10). Two types of setae were present, with the robust setae being simple and approximately 4-5 μm in diameter, and elongate fine setae being approximately 0.3-1.2 μm in diameter and about 3 mm in length (Fig. 7a, b). Bromine was found to be accumulated only on the robust setae. Unlike other parts of the body, some bromine accumulation was also detected on the surface of the pleopods (Fig. 7c, Table S3).

On the uropods, we also found bromine accumulating on the distolateral robust setae, which were approximately 10–15 μm in diameter and 80–100 μm in length (Fig. 8, Fig.

S11). The overall surface of the uropods including the setae lacked bromine accumulation, unlike in the pleopods (Fig. 8d, Table S3).

The dorsal external armature of *H. gigas*, on the other hand, did not exhibit any specific bromine accumulation, from our observations of the coxal plates (Fig. 7, Fig. S12) and the pereonites (Fig. S13). Both coxal plates and pereonites were composed of hexagonal elements approximately 30 μm in size, which were more clearly visible in the coxal plates (Fig. 7b). High-magnification images of the dorsal armature revealed numerous bead-like structure arranged in nearly parallel lines transversing the body, with the typical spacing between the lines being 669 ± 184 nm (N = 36). Elemental mapping of did not exhibit any specific localization of bromine related to surface structure[illegible] also indicated in area analyses (Fig. 7c, Table S3), while the sum spectra showed small amount of bromine.

Discussion

Elemental analysis using electron microscopy

Our results showed that what was previously thought to be aluminum signals from *Hirondellea gigas*[8] was actually bromine. This misassignment of element can be traced back to issues with the sample preparation and selection of electron microscopic imaging conditions in Kobayashi *et al.*[8] Although high-resolution imaging can be performed without any coatings by using low-landing voltage techniques under low beam current,[22,23] EDS measurements require doubled voltage of maximum characteristic X-ray energy of target elements~~, and~~ large current is required for X-ray emission sufficient for elemental mapping. The spectrum acquisition condition is prone to cause charge-up, as electrons can penetrate deep into the insulative organic specimen. In addition, internalized high-energy electrons can escape from the side of curved surface, resulting in enhanced counts of low-angle backscattered electrons – thus the total intensity of X-rays from SEM chamber and substrate at the sample edge, which corresponds to the previous results where aluminum signals were mostly detected ~~in~~ the edges (fig. 1 in [8]). The use of aluminum stub and aluminum-

reinforced double-sided carbon tape, as in [8], is not recommended for aluminum detection for these reasons. Silicon is also an element to be ~~cautious about,~~ because EDS detector window is made of silicon, which can be excited either by backscattered electrons ~~and~~ incoming high-energy X-ray, the latter known as the escape peak.

One criterion to distinguish whether the EDS signals in map images truly originate~~s~~ from elemental localization or are artefacts, is to compare the image with total counts at each pixel, or counts per seconds (cps) images. Uncorrected X-ray maps plot X-ray intensities of a given X-ray energy range at each position, including bremsstrahlung X-rays, which ~~is~~ correlated to the total emitted X-ray. When the relative intensities of X-ray maps and the cps map of the area concerned are similar in ratio, the element localization ~~is~~ in doubt. Note that conventional X-ray corrections such as ZAF correction is not always suitable for EDS analysis of non-flat specimens. Increasing the beam energy is another method to distinguish among elements. In the particular case of aluminum K-line (1.486 keV), bromine L-line (1.480 keV) and two lanthanides absent in animals (thulium and ytterbium)[12] can overlap with it at the energy resolution of EDS (typically ~130 eV). By using high-energy electron beams bromine K-line (11.92 keV) can be detected without overlapping with other elements. High-energy X-rays have less intensities than low-energy ones, and the use of high-sensitivity detectors is desired. The use of semi-thin sections, comparison of X-ray maps and cps maps, irradiation of high-energy electron beam, and use of high-sensitivity detector in our ~~analytic~~ methods ensure that we ~~can~~ avoid such pitfalls ~~of artifacts~~.

In our measurements, sea salt sometimes appeared on the surface, which is an artefact ~~during~~ drying ~~because~~ we did not wash the tissues thoroughly to prevent removal of fragile parts. However, ~~the sea~~ salt ~~does~~ not affect the amount of bromine because the molar amount of bromine is less than 1% of that of sodium, the main content of the sea salt.

Origin of bromine and its biological implication

Bromine is the eighth abundant elements in the ocean after chlorine, sodium, magnesium, sulfur, calcium, potassium, and carbon.[13] Although the explicit amount of bromine in the Izu-Ogasawara Trench is not reported,[24] the relative amount of bromine is expected to be similar to that of standard seawater. A possibility is the simple adhesion of bromine, but the absence of chemically similar chlorine denies this possibility. Rather, it is more plausible to consider that bromine was secreted by the amphipod, most preferentially to the gastric mill but also to the robust setae on various parts of the body, as well as the surface of the pleopods.

Accumulation of bromine has been also found on other marine organisms. In terms of small molecules extracted from marine organisms, the marine snail *Bolinus bandaris* has been historically important as the source of Tyrian purple whose structural isomers have 6-bromo-3-indolinone structure,[25] tripeptide barettin from the marine sponge *Geodia barretti* has dehydrogenated 6-bromotryptophan,[26] and the pentabrominated phenylpyrrole is found as an antibiotic compound from marine bacteria.[27,28] Bromine is attached on aromatic rings in all cases. There are also cases of bromine in the hard parts of marine organisms whose molecular structure has not been identified, such as the fish otolith which contain bromine and chlorine colocalized as minor elements.[29] Such cases of bromine in hard parts are also known from at least two cases where the hard parts have predation or feeding-related functions. In the crab *Pachygrapsus crassipes*, bromine is enriched on the tips of its calcified claws and mechanical tests have shown that this makes the tips less hard but more fracture-resistant.[14,30] Another case is the zinc-based jaws of the marine polychaete worm *Nereis virens* that also contain chlorine, manganese, and iodine; brominated amino acids such as histidine might bind to metals strongly to prevent dissolution. [2,3]

The hadal amphipod *H. gigas* is unique among these animals in that the bromine does not coexist with other halogens and it is poor in other transition metals within the 1-um thick organic coatings containing bromine. In *H. gigas*, bromine is expected to reinforce the gastric mill like other animals' jaws, considering its biological role in crushing its food.

The localization of bromine on the sclerotized spines and the lack of it in the calcified ossicles itself is likely beneficial in that bromine renders the spines less susceptible to breaking, in order to maintain its sharpness and function in food sorting. The ossicles are used for actual crushing, and as such they are beneficial to be calcified to increase the hardness. The same has been suggested for the function of the forcep-like, bromine-enriched, crab claw tips versus the rest of the claw used for crushing, which is calcified and lacks bromine.[14] The form of bromine in the gastric mill and setae remains unclear. The coexisting nitrogen and sulfur with bromine indicate the presence of amino acids, which may imply that the bromine is incorporated in aromatic amino acids, such as phenylalanine and tryptophan, in which case the bromine is most likely secreted by *H. gigas*. The function of bromine on the setae of *H. gigas* remains unclear at this point, although it may be secreted in a similar manner to the spines in the gastric ossicles.

Using calcite as the exoskeleton in depth deeper than the CCD (typically <5000 m) typically results in dissolution, and how *H. gigas* can retain its calcite armature requires a reconsideration now that it has become clear that it does not possess an 'aluminum shield' like previously suggested.[8] EPMA showed the existence of magnesium in the calcite layer with the Mg/Ca ratio being around 2–20% (Fig. S3). The absence of chlorine in the dried specimen indicates that the magnesium is not from the remaining seawater salt but it is within the exoskeleton. Water solubility of calcite decreases by partial substitution of calcium with magnesium, i.e. $(Ca,Mg)CO_3$,[31,32] and the substitution would partially prevent carbonate dissolution below the CCD. Though phosphorous was also present in considerable amount, its role is ambiguous as XRD did not provide the evidence of calcium phosphate minerals.

Conclusion

In summary, we revealed the presence of bromine and magnesium in the hadal amphipod *Hirondellea gigas*, and showed that it does not actually possess an 'aluminum shield' as previously thought, through careful prevention of aluminum artefacts. Instead of

the 'aluminum gel' shielding, the use of magnesium-substituted calcite may be the key for the amphipod to survive without demineralization below the CCD. High concentration of bromine ~~was~~ found on the sclerotized spines in the gastric mill, forming a 1 μm-thick coating layer on mineralized tissue with a relatively high concentration of nitrogen. The bromine-coating on the gastric mill spines is speculated to have the function of maintaining a sharp tip for food processing, similar to what has been suggested for crab claw tips. Our study serves as a reminder of the importance and necessity of appropriate sample preparation and careful analysis of the signals in EDS analysis, as well as the importance ~~to analyze~~ at different electron energies when performing microscopic[illegible]emental anatomy'.

Acknowledgements

The authors thank the Captain and crew of R/V *Yokosuka* during expeditions YK19-11 (cruise PI: Akinori Yabuki, JAMSTEC) for their great support of the scientific activity. All scientists on-board the expeditions are gratefully acknowledged for their tireless work, especially Heather Ritchie Parker who assiste[illegible]e preservation of the amphipod specimens. We also thank Kana Nagashima (JAMSTEC) for her kind offer for XRD analysis. We thank JSPS for financial support with a [Grant-in-Aid for Young Scientists Grant Number JP19K15379] to S.O. and a [Grant-in-Aid for Scientific Research[illegible]ant Number 18K06401] to C.C. and H.K.W.

Author contributions

S.O., C.C., and K.T. designed the experiments. N.I. collected and preserved the amphipod on-board the research vessel. S.O. performed sample preparation and electron microscopic observation. S.O. and N.I. performed XRD analysis. S.O., H.K.W., and C.C. dissected the amphipod. All authors contributed to the writing of this manuscript and approved its submission and publication.

Conflict of interest

The authors declare no competing financial interest.

Supporting Information

Additional figures of SEM images, EDS maps, EDS spectra, XRD data, and tables of cross correlation data in EPMA, and elemental analyses based on EPMA and EDS.

Figure Legends

Figure 1. EDS maps of semi-thin sections of *H. gigas*. (a) transverse section through a subsample of pereonite, (b) transverse section of the cephalic region showing the gastric mill, (c) magnified images of the gastric mill, the red rectangle area in (b). Images from the left are SEM images at (a,b) 1 kV and (c) 10 kV, total X-ray counts (grayscale), phosphorous (green), calcium (blue), and aluminum-like signals around 1.48 keV (silver). EDS spectra were acquired at 10 kV, thus the K-line of bromine was not observed.

Figure 2. X-ray spectra and elemental maps of a semi-thin section of the gastric mill in *Hirondellea gigas*. (a) SEM image of the mapped area. (b) Point analysis at the red point in (a). (c) WDS elemental maps of the orange rectangle in (a). Note that Al K contrast is significantly weaker than that of Br K.

Figure 3. SEM images and elemental maps of a semi-thin section of the pereonite in *Hirondellea gigas*. (a) Low-magnification SEM image. (b) High-magnification SEM image of the red rectangle in (a). (c) WDS elemental maps of the orange rectangle in (b).

Figure 4. SEM/EDS images of the gastric mill of *Hirondellea gigas*. A) low-magnification SEM image, b) magnified image of the spine, yellow rectangle in part (a), c) magnified image of the filter setae, orange rectangle in part (a), d) EDS elemental mapping and sum spectrum

of the red rectangle in part (a). e) Line profile of the Br K intensity in EDS map along with the longitudinal axis of the ossicle spines. The line profile was measured with the line width of 10 μm.

Figure 5. SEM/EDS images of maxilla 2 of *Hirondellea gigas*. A) low-magnification SEM image, b) magnified image of the yellow rectangle in part (a), c) EDS elemental mapping and sum spectrum of the red rectangle in part (a).

Figure 6. SEM/EDS images of an antenna of *Hirondellea gigas*. A) low-magnification SEM image, b) magnified image of the yellow rectangle in part (a), c) EDS elemental mapping and sum spectrum of the red rectangle in part (a).

Figure 7. SEM/EDS images of a pleopod of *Hirondellea gigas*. A) low-magnification SEM image, b) magnified image of the yellow rectangle in part (a), c) EDS elemental mapping and sum spectrum of the red rectangle in part (a).

Figure 8. SEM images and EDS maps of dissected dry uropod of *Hirondellea gigas*. (a) Low-magnification image. (b) High-magnification image of the yellow square in (a). (c) Sum spectrum and EDS maps of the red square in (a). The sum spectra at 10–15 kV were magnified and displayed in the inset above with the same energy scale for clarity.

Figure 9. SEM/EDS images of a coxal plate of *Hirondellea gigas*. A) low-magnification SEM image, b) magnified image of the yellow rectangle in part (a), c) EDS elemental mapping and sum spectrum of the red rectangle in part (a).

References

1. Meenakshi, V. R., Hare, P. E., Watabe, N. & Wilbur, K. M. The chemical composition of the periostracum of the molluscan shell. *Comp Biochem Physiol* 29, 611–620 (1969).

2. Broomell, C. C., Zok, F. W. & Waite, J. H. Role of transition metals in sclerotization of biological tissue. *Acta Biomater* 4, 2045–2051 (2008).

3. Broomell, C. C., Mattoni, M. A., Zok, F. W. & Waite, J. H. Critical role of zinc in hardening of Nereis jaws. *J. Exp. Biol.* 209, 3219–3225 (2006).

4. Brandao, S. N. *et al.* Review of Ostracoda (Crustacea) living below the Carbonate Compensation Depth and the deepest record of a calcified ostracod. *Progress Oceanogr.* 178, (2019).

5. Bourget, E. Barnacle shells: Composition, structure and growth. in *Barnacle Biology* (ed. Southward, A. J.) (Taylor & Francis, 1987). doi:10.1201/9781315138053.

6. Kobayashi, H., Hatada, Y., Tsubouchi, T., Nagahama, T. & Takami, H. The Hadal Amphipod Hirondellea gigas Possessing a Unique Cellulase for Digesting Wooden Debris Buried in the Deepest Seafloor. *PLoS ONE* 7, e42727–8 (2012).

7. Kobayashi, H. *et al.* Polysaccharide hydrolase of the hadal zone amphipods Hirondellea gigas. *Biosci. Biotechnol. Biochem.* 1–11 (2018) doi:10.1080/09168451.2018.1459178.

8. Kobayashi, H., Shimoshige, H., Nakajima, Y., Arai, W. & Takami, H. An aluminum shield enables the amphipod Hirondellea gigas to inhabit deep-sea environments. *PLoS ONE* 14, e0206710 (2019).

9. Suri, A. *et al.* Analysis and detection of low-energy electrons in scanning electron microscopes using a Bessel box electron energy analyser. *J. Elec. Spec. Rel. Phen.* 241, 146823 (2020).

10. Bearden, J. A. X-ray wavelengths and x-ray atomic energy levels. *Rev. Mod. Phys.* 39, 78–124 (1967).

11. Newbury, D. E. Mistakes encountered during automatic peak identification in low beam energy X-ray microanalysis. *Scanning* 29, 137–151 (2007).

12. Cotruvo, J. A. The Chemistry of Lanthanides in Biology: Recent Discoveries, Emerging Principles, and Technological Applications. *ACS Cent Sci* 5, 1496–1506 (2019).

13. Millero, F. J., Feistel, R., Wright, D. G. & McDougall, T. J. The composition of Standard Seawater and the definition of the Reference-Composition Salinity Scale. *Deep Sea Res Part Oceanogr Res Pap* 55, 50–72 (2008).

14. Schofield, R. M. S. *et al.* The homogenous alternative to biomineralization: Zn- and Mn-rich materials enable sharp organismal "tools" that reduce force requirements. *Sci. Rep.* 11, 1–23 (2021).

15. Luft, J. H. Improvements in epoxy resin embedding methods. *J. Biophys. Biochem. Cytol.* 9, 409–414 (1961).

16. Du, K., Hochmeister, K. von & Phillipp, F. Quantitative comparison of image contrast and pattern between experimental and simulated high-resolution transmission electron micrographs. *Ultramicrosc.* 107, 281–292 (2007).

17. Joy, D. C. & Pawley, J. B. High-resolution scanning electron microscopy. *Ultramicrosc.* 47, 80–100 (1992).

18. Postek, M. T. & Vladár, A. E. Does Your SEM Really Tell the Truth?–How Would You Know? Part 4: Charging and its Mitigation. *Proc SPIE Int Soc Opt Eng* 9636, 963605 (2015).

19. Cauchie, H.-M. Chitin production by arthropods in the hydrosphere. *Hydrobiologia* 470, 63–95 (2002).

20. Sugawara, A. *et al.* Self-Organization of Oriented Calcium Carbon- ate/Polymer Composites: Effects of a Matrix Peptide Isolated from the Exoskeleton of a Crayfish. *Angew. Chem. Int. Ed.* 45, 2876–2879 (2006).

21. Becker, C., Dick, J. T. A., Cunningham, E. M., Schmitt, C. & Sigwart, J. D. The crustacean cuticle does not record chronological age: New evidence from the gastric mill ossicles. *Arthropod Structure and Development* 1–15 (2021) doi:10.1016/j.asd.2018.07.002.

22. Harano, K., Okada, S., Furukawa, S., Tanaka, H. & Nakamura, E. Formation of a polycrystalline film of donor material on PEDOT:PSS buffer induced by crystal nucleation. *J. Polym. Sci. Part B: Polym. Phys.* 52, 833–841 (2014).

23. Gorgoll, R. M., Harano, K. & Nakamura, E. Nanoscale Control of Polymer Assembly on a Synthetic Catalyst-Bilayer System. *J. Am. Chem. Soc.* 138, 9675–9681 (2016).

24. Obata, H. *et al.* Dissolved iron and zinc in Sagami Bay and the Izu-Ogasawara Trench. *J Oceanogr* 73, 333–344 (2017).

25. Cooksey, C. Tyrian purple: the first four thousand years. *Sci Prog* 96, 171–186 (2013).

26. Sölter, S., Dieckmann, R., Blumenberg, M. & Francke, W. Barettin, revisited? *Tetrahedron Lett* 43, 3385–3386 (2002).

27. Lovell, F. M. The structure of a bromine-rich marine antibiotic. *J. Am. Chem. Soc.* 88, 4510–4511 (1966).

28. Burkholder, P. R., Pfister, R. M. & Leitz, F. H. Production of a pyrrole antibiotic by a marine bacterium. *Appl Microbiol* 14, 649–653 (1966).

29. Arai, N., Sakamoto, W. & Maeda, K. Analysis of Trace Elements in Otoliths of Red Sea Bream Pagrus major. *Fisheries science* 61, 43–47 (1995).

30. Schofield, R. M. S. *et al.* Br-rich tips of calcified crab claws are less hard but more fracture resistant: A comparison of mineralized and heavy-element biological materials. *J Struct Biol* 166, 272–287 (2009).

31. Mucci, A. & Morse, J. W. The solubility of calcite in seawater solutions of various magnesium concentration, It = 0.697 m at 25 ° C and one atmosphere total pressure. *Geochim. Cosmochim. Acta* 48, 815–822 (1984).

32. Morse, J. W., Mucci, A., Walter, L. M. & Kaminsky, M. S. Magnesium interaction with the surface of calcite in seawater. *Science* 205, 904–905 (1979).

Figure 1

Click here to access/download;Figure;Figure1.tiff

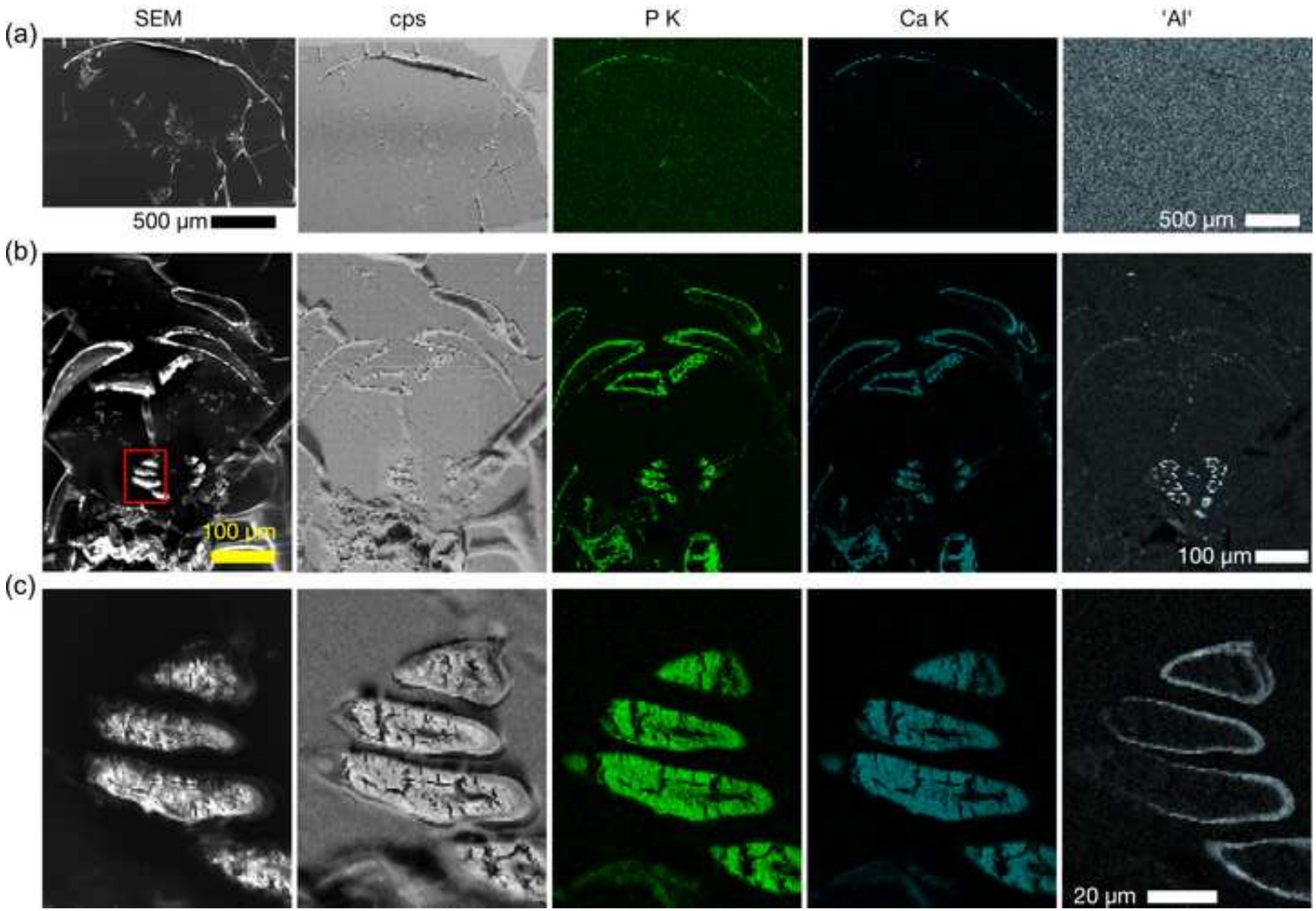

Figure 2

Click here to access/download;Figure;Figure2.tiff

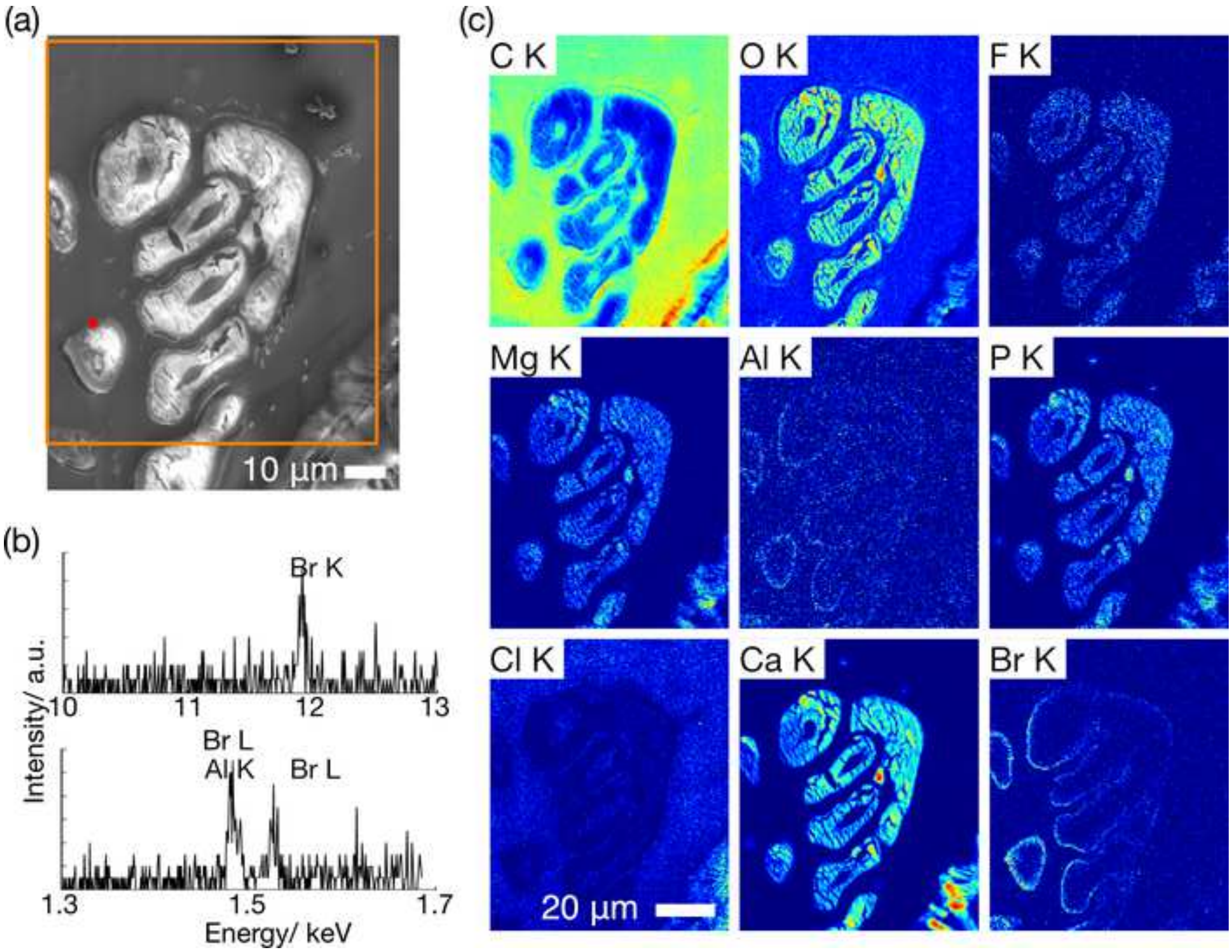

Figure 3

Click here to access/download;Figure;Figure3.tiff

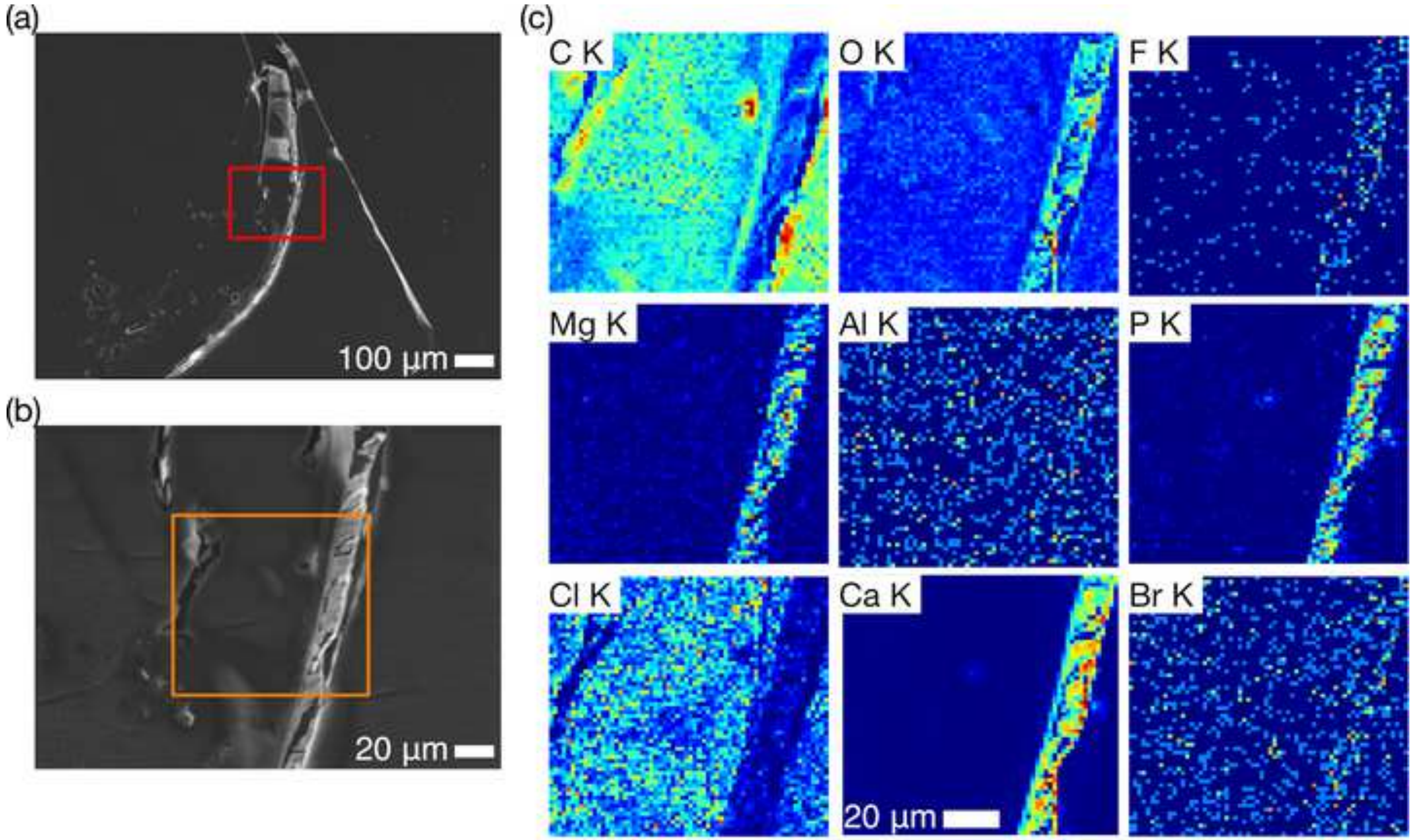

Figure 4

Click here to access/download;Figure;Figure4.tiff

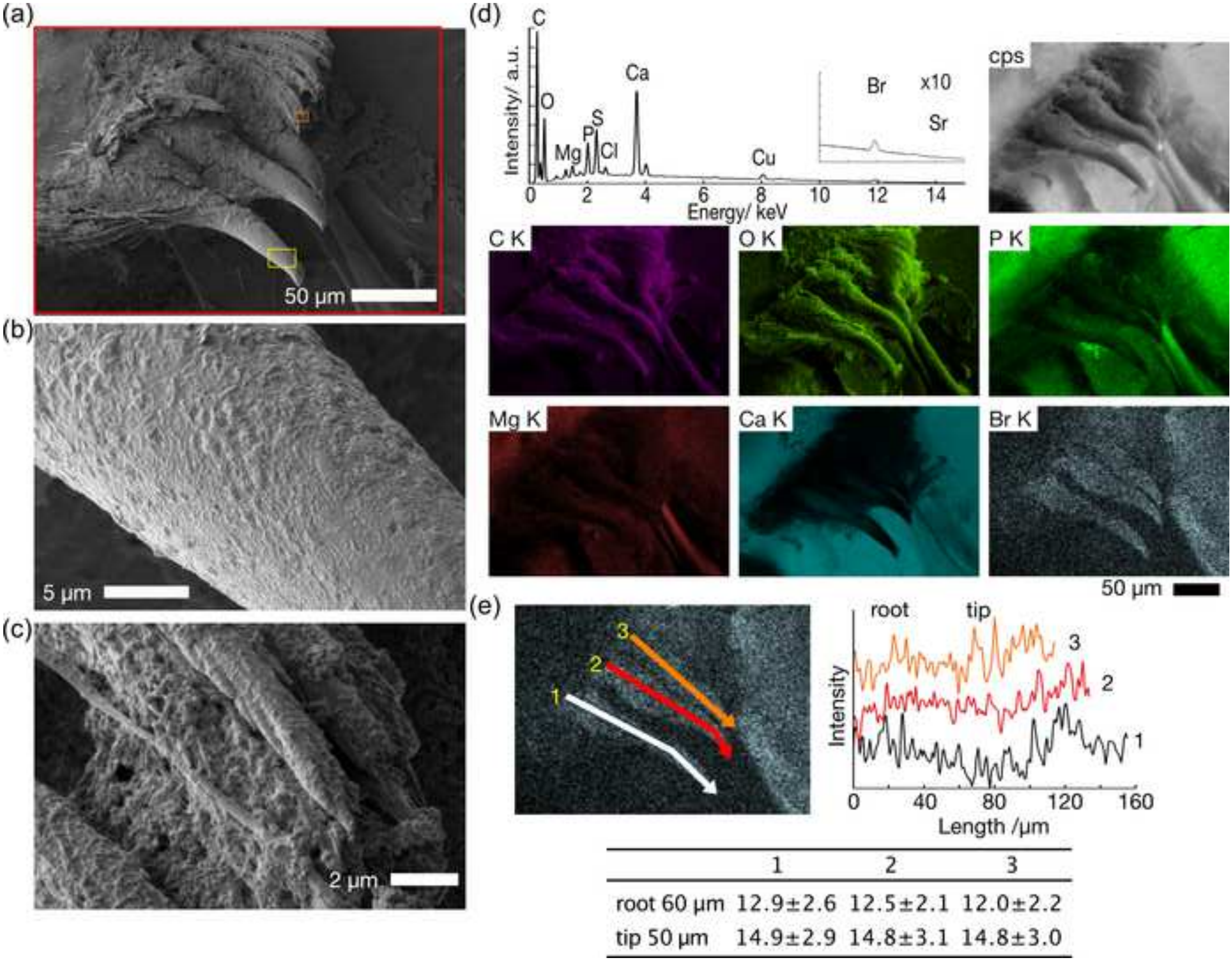

	1	2	3
root 60 µm	12.9±2.6	12.5±2.1	12.0±2.2
tip 50 µm	14.9±2.9	14.8±3.1	14.8±3.0

Figure 5

Click here to access/download;Figure;Figure5.tiff

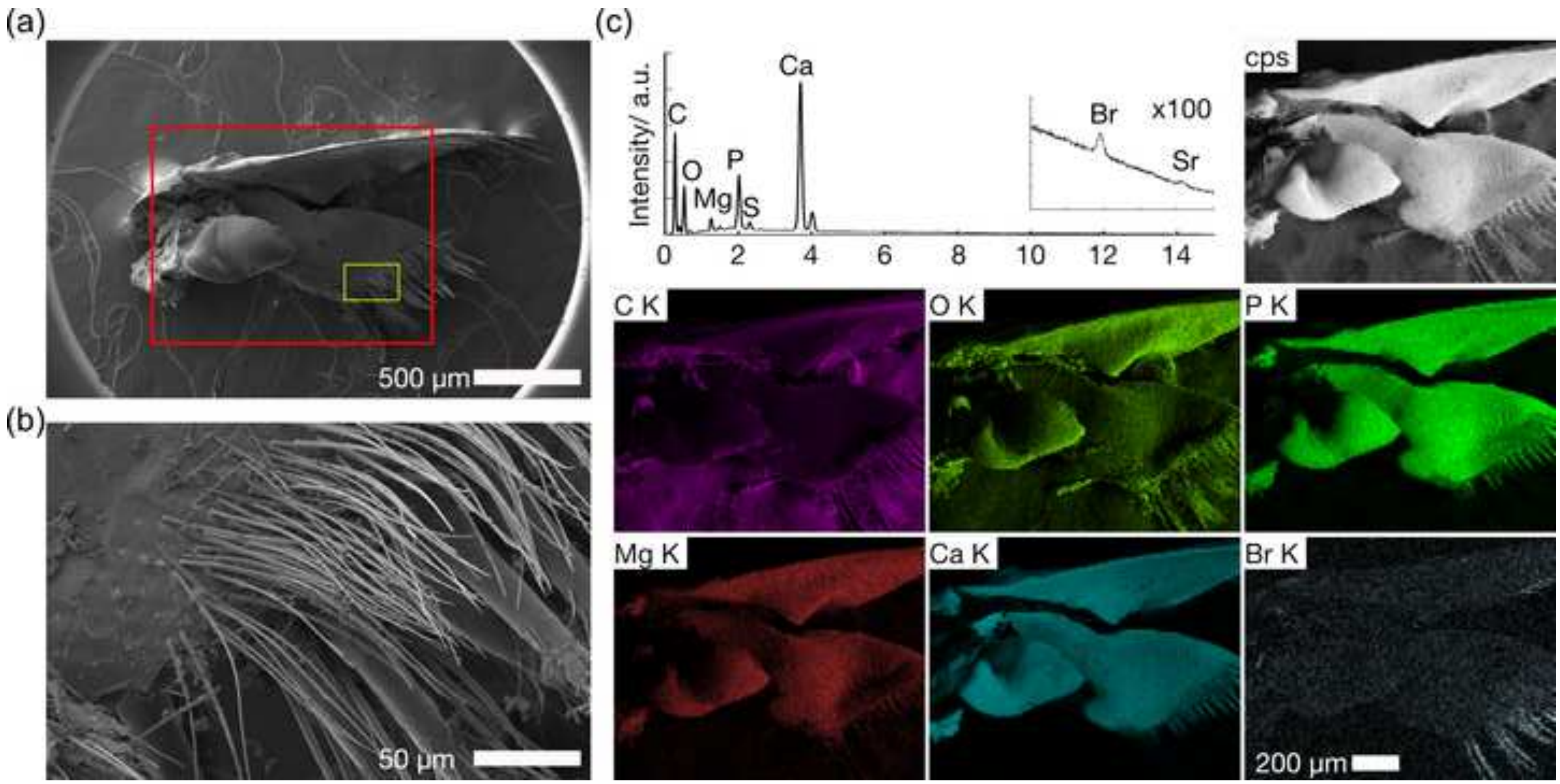

Figure 6

Click here to access/download;Figure;Figure6.tiff

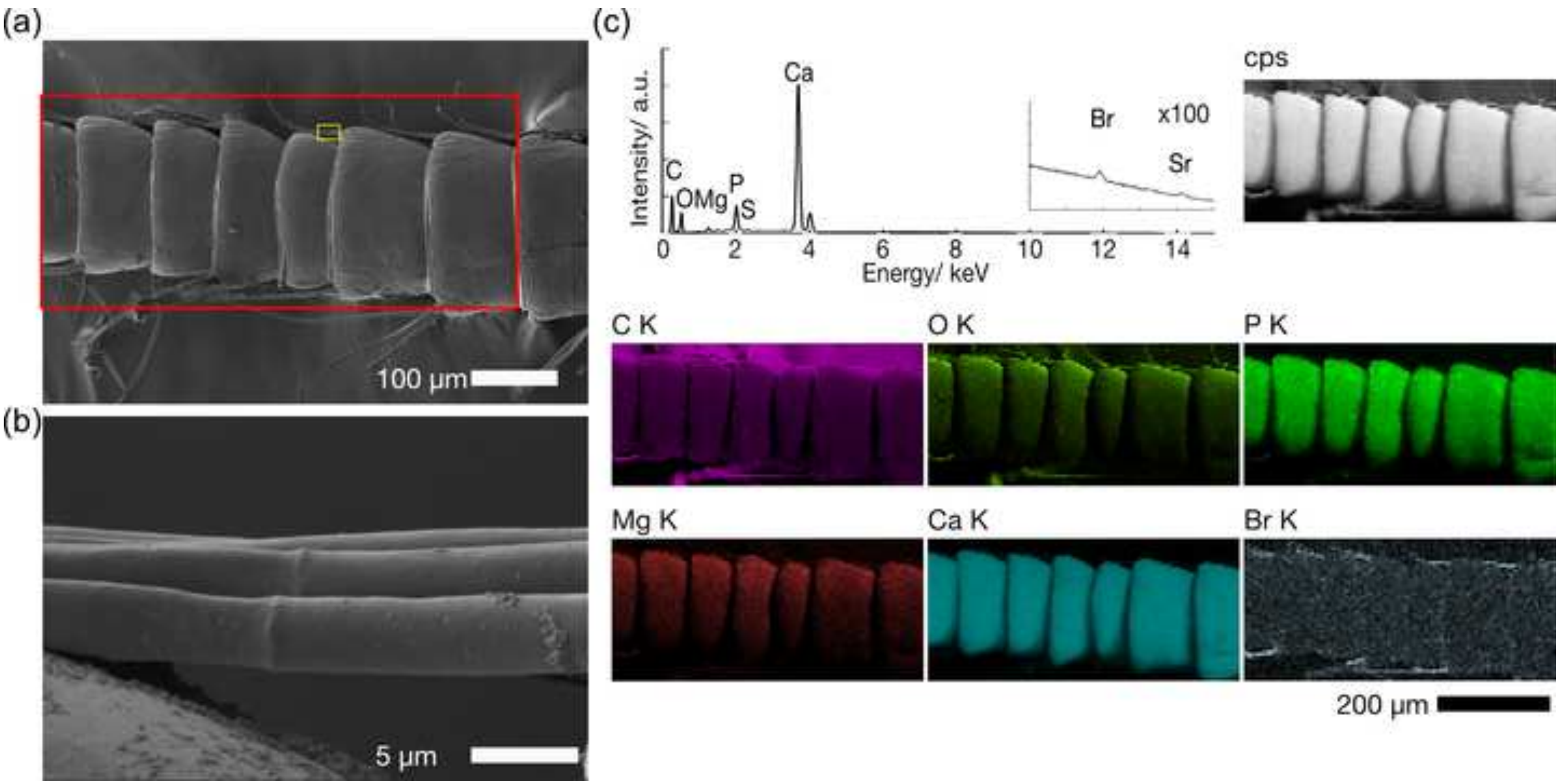

Figure 7

Click here to access/download;Figure;Figure7.tiff

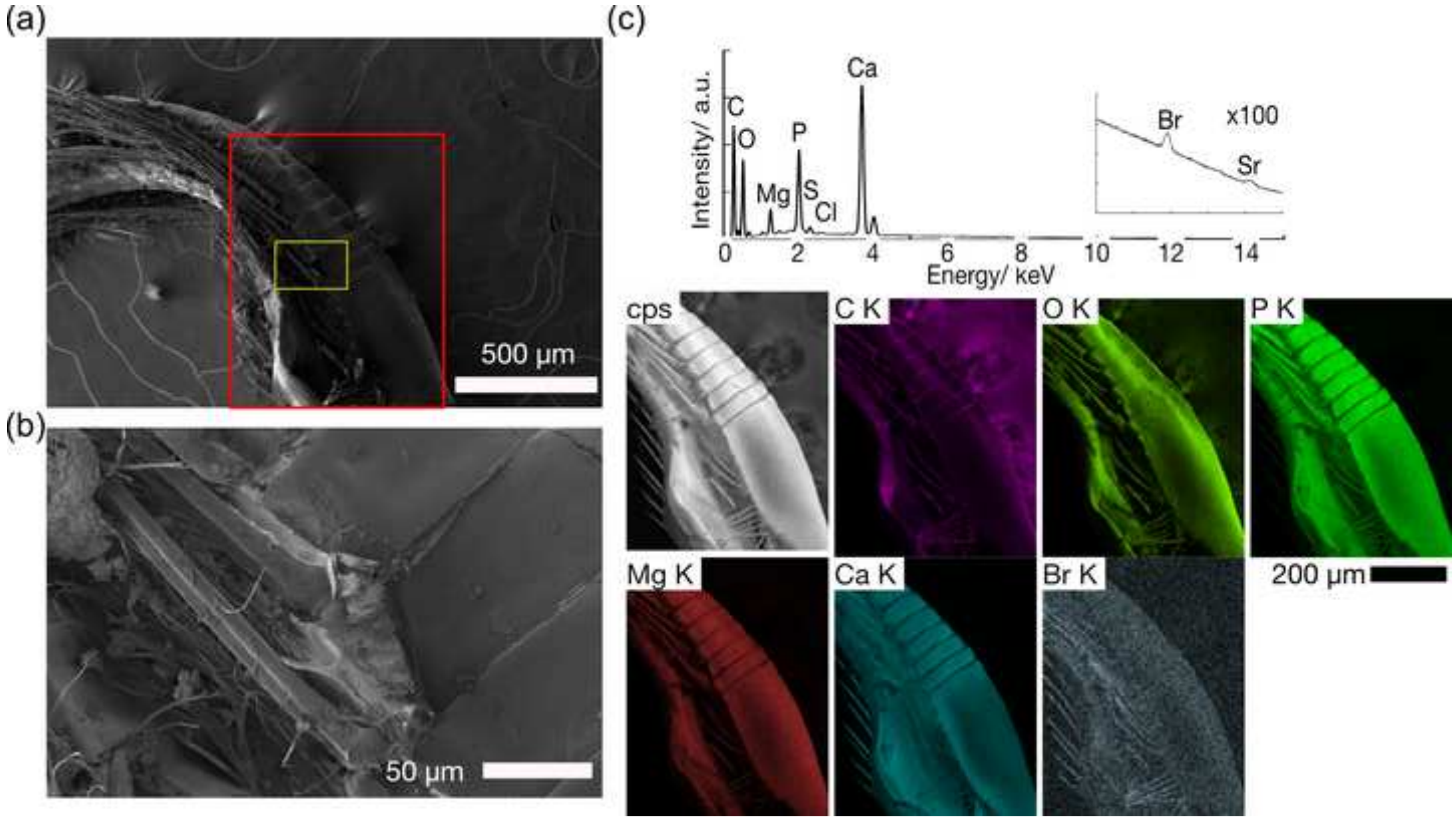

Figure 8

Click here to access/download;Figure;Figure8.tiff

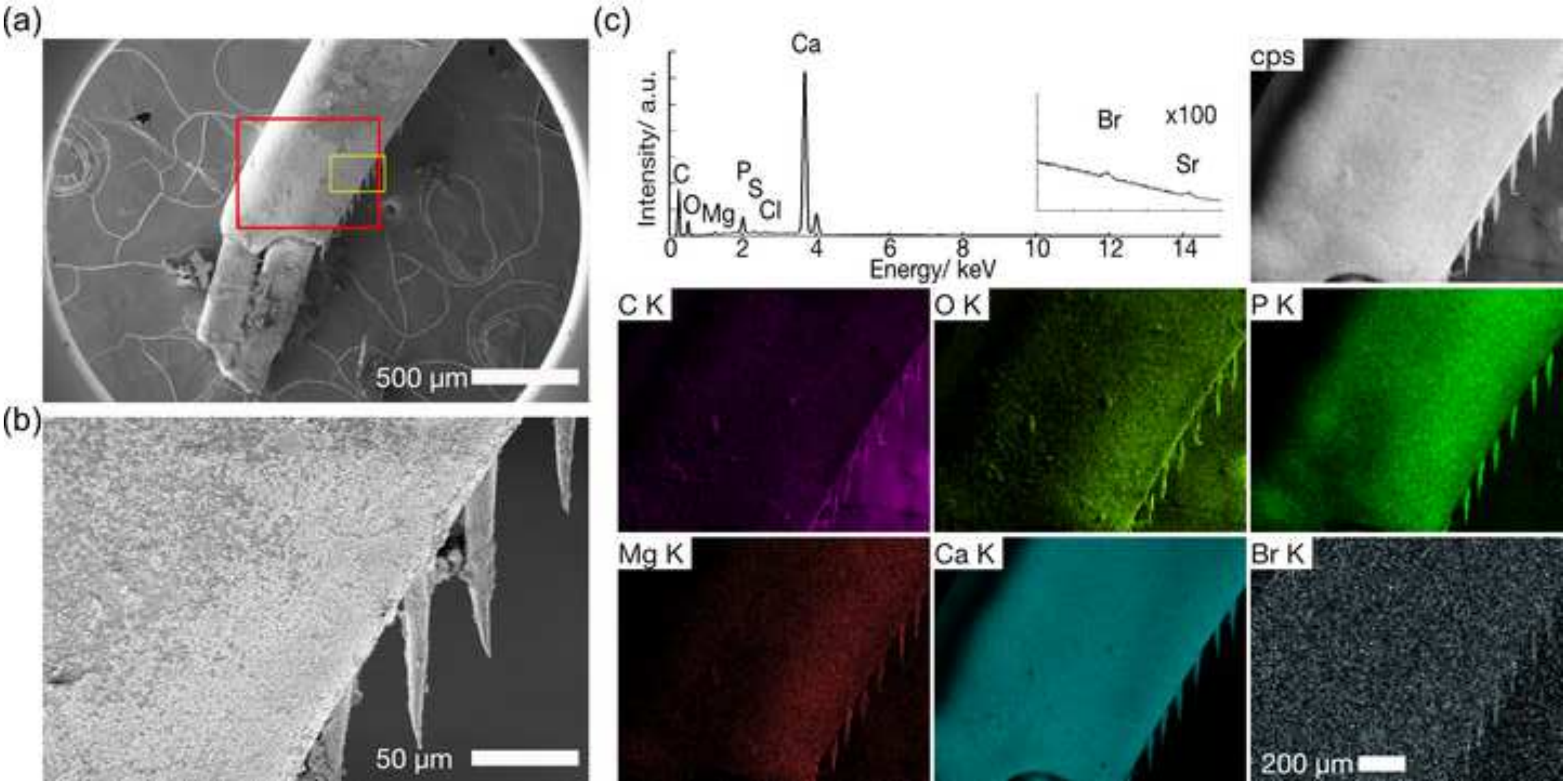

Figure 9

Click here to access/download;Figure;Figure9.tiff

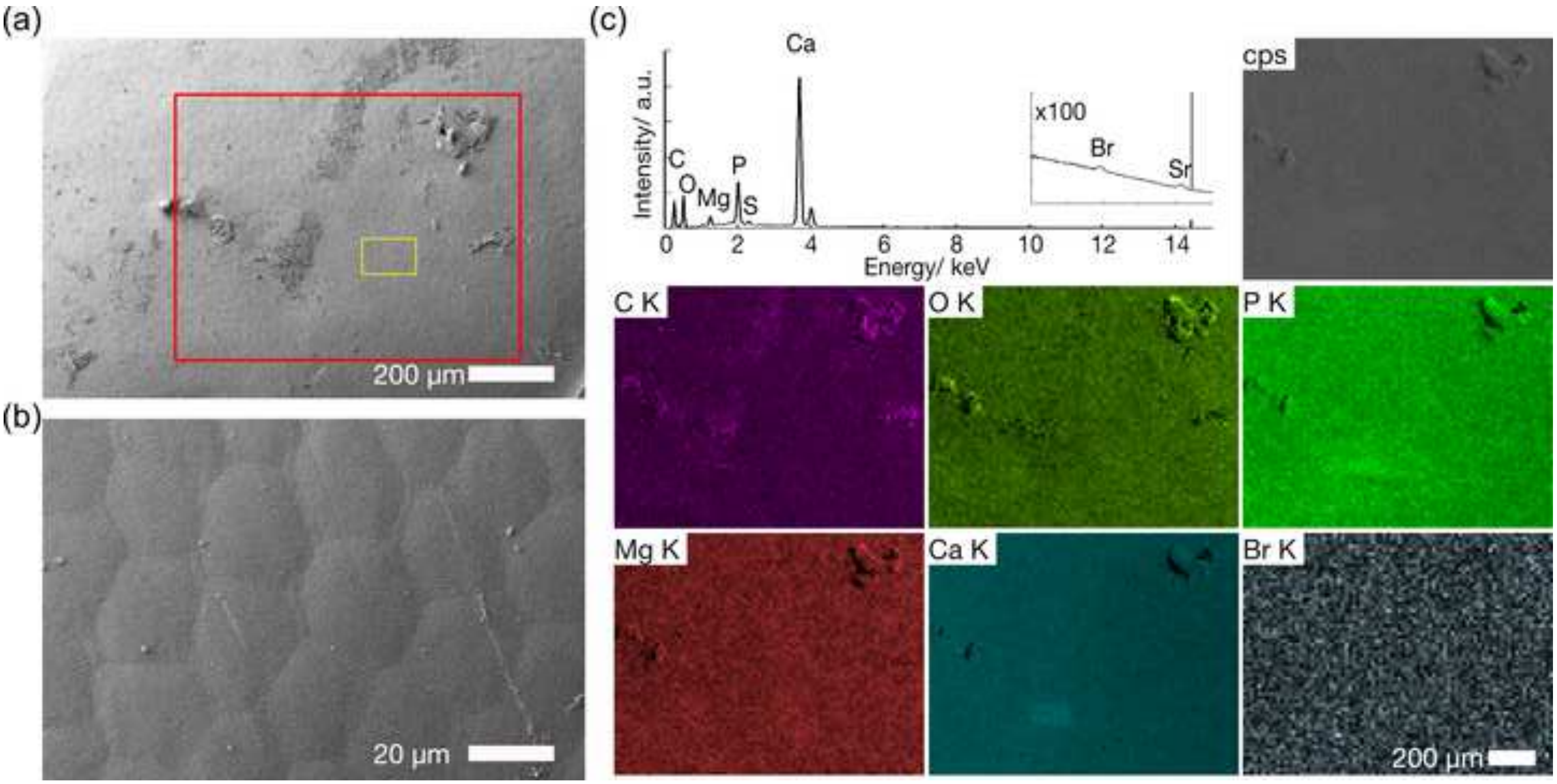

Click here to access/download
Supporting Information
okada_supplement.docx
