# Supplementary material for: Unusual bromine enrichment in the gastric mill and setae of the hadal amphipod Hirondellea gigas
Source: PLoS One. 2022 Aug 4;17(8):e0272032. doi: 10.1371/journal.pone.0272032 (PMC9352070; doi:10.1371/journal.pone.0272032)
Supplement: Attachment — Submitted filename: PLOS One_Review Document for Okada et al.pdf [file pone.0272032.s017.pdf]

Review of “Unusual bromine enrichment in the gastric mill and setae of the hadal amphipod *Hirondellea gigas*” by Okada, et al.

I have reviewed the ms by Okada, et al, regarding the absence of an aluminum gel shield in the deep-water amphipod, *H. gigas* and the presence of bromine, instead, demonstrating the importance of careful and proper SEM analyses of samples for ‘elemental anatomy.’ I found the manuscript (ms) of interest and of potential interest to the PLOS One audience of readers. However, although the work appears to be thorough and meticulously done, demonstrating careful analytical rigor, I have, nevertheless found the writing to be rather simplistic with faults in the logical flow of the paper. It appears more analogous to an undergraduate laboratory report, than a ms for publication, and for the most part, is poorly prepared.

For these and the following reasons, I do not recommend the publication of this work until it has been carefully recrafted and revised according to my comments below. I have provided a PDF of the text with my many small comments and edits, which I recommend the authors carefully consider and revise the ms accordingly. In addition, I am providing in this document, a list of both Minor and Major areas of revisions for consideration by the authors. I hope the comments provided facilitate the revising of the ms and are helpful to the authors. I believe that once revised, the paper would be of interest and use to other researchers with interests in the area of deep see arthropods and their anatomy and physiology.

Additionally, not having read the Instructions for Authors, I wonder why the ms does not have line numbers and why the many supplemental figures and tables were not provided for the purposes of review. Having line numbers facilitates a much easier review process for the reviewer and authors. I recommend that manuscripts NOT be sent to reviewers by the Editors unless continuous line numbers have been included!!

Minor Revisions

There are a plethora of small grammatical errors in the text which I have endeavored to correct (as far as I could understand some of the sentences written). I recommend the authors have a native science writer review the text before resubmitting the revised ms to the journal (or any journal).

Abstract

L??? – the code EDS is used. However, EDS is never spelled out in the prior text. This needs to be spelled out in full the first time it is used, as here in this sentence.

Main Text

L??? – second para. The autors use the term “megafauna” for *H. gigas*. This is incorrect. *H. gigas* would be considered macrofauna, *not* megafauna. Please check the definitions of these.

L??? – the authors state, “The aluminum shield was expected to…” Who was it that was expecting this, and what is the citation/reference for this?

L??? - ??? – “The aluminum shield…Challenger Deep.” This sentence is long and convoluted. It makes little sense, and also needs a reference at the end to state who was expecting this gel shield.

L??? - ??? – first part of first paragraph on pg. 3. “In order to confirm…artefacts.” This sentence should be moved to the end of the Introduction section as a segway into the methods section.

L??? - ??? – “There are two possibilities where aluminum…latter two being unused in animals.” This section should be moved to the top of the first full paragraph on Pg. 3.

L??? - ??? “Our detailed analysis revealed…harden the hard parts.” This entire section belongs in the Results section, not in the introduction section. It is not part of the literature review or the justifications for the study.

Methods

Amphipod Samples

L??? - ??? – it would be helpful to provide a map of the study location as Fig. 1.

L??? - ???. In this section, no mention is made of how many amphipod samples were actually collected in total, and neither is a total sample size mentioned in the Results.

Electron microscope instruments.

L??? – “…2k x 2K…” us the multiplication symbol (×), not the letter “x”

L??? – “The dehydrated amphipod was replaced with QY-1…” Is this really what the authors meant to say; that the actual animal was replaced with QY-1? I do not understand this. Should this mean something, such as “fluid from the amphipod was replaced by QY-1…”?

L??? “…HighScore 3.0.5…” Is this hardware or software? It is not clear and not stated. Please clarify.

L??? – “…maxilla (Fig. 5, Fig. S5).” Which part of Fig. 5? Nothing specific about Fig. 5 is mentioned or explained in the text. As well, why do you need a S fig for almost every fig in the text. This seems to me to be very redundant.

Discussion

L??? - ??? – “A possibility [of what?] is the simple…this possibility.” This sentence does not make sense. What are the authors trying to say?

L??? - ??? – “In terms od small molecules…compound from marine bacteria.” This is a run-on sentence that makes little sense. Please rewrite and break into at least 2 sentences.

Major Revisions

Main Text

L??? – end of first paragraph. There is need to provide a more thorough and exhaustive introduction to the possible impregnation of Crustacean exoskeletons with other materials. Here, several examples from the previous literature should be provided.

Methods

Precautions to avoid artificial aluminum signals

L??? - ??? – "…due to three benefits. Firstly, the spatial resolution…reflect the sample's true elemental composition." Since this is actually a discussion of the benefits of the process, these should NOT be presented here in the Methods section, but instead should be used to discuss the results and support them as benefits of the process undertaken by the authors. Having them in the Methods does not make sense – they are NOT methods, but explanations of the results.

Results

It is never mentioned in the Results how many specimens of *H. gigas* were actually collected from the expedition and used in the study. This needs to be provided.

L??? – "…1.48 keV (Fig. 2)." Authors need to be specific about what part of Fig. 2 they are referring to; A, B, or C? Also, here in the results, they should refer to each part of Fig. 2, including A, B, and C.

In this same area of the text, the authors mention a plot of two peaks. However, in Fig. 2B, they have two separate plots of peaks. Which is which? Explain both and refer to both in the text. Need to be very specific here.

L??? – "The elemental colocalization…in the cross-section tissues." All of this section should be removed and used in the Discussion section, NOT here in Results.

L??? – "…(Fig. 8d…)." There is no "d" labelled in Fig. 8.

L??? – "…the coxal plates (Fig. 7, Fig. S12)…" I do not believe these should be referring back to Fig. 7, but instead to Fig. 9. At this point the ms has a total of 21 figures. This seems to me to be a huge number of figs. Are they all really necessary? I do not know about the supplemental figs because I did not see that they were included with the ms for review.

L??? - ??? – "However, salt did not affect the amount of bromine…sea salt." This does not appear to be a valid explanation as to why sea salt would not affect bromine. Are there no bonding interactions, no crystal structure influences?? Explain your point here and provide support from the literature for your stance.

General

Figures have many parts and sections, yet authors do not refer to the parts and sections in reporting the results. This makes it VERY confusing as to which part of the figure is being described in the Results text.

Again, I do not understand why there are so many supplemental figures – one for every figure presented in the text, and more! Are all of these truly necessary to comprehend the research done?

In general, there appears to be a lack of understanding of how to formulate an ms for publication. The authors need to carefully outline the ms so that there is logical flow from one section to the next, and ensure that within the major sections of the paper (i.e. Introduction, Methods, Results, Discussion), that they keep within those boundaries of the sections. Do not put discussion into the Methods, or Results in the Introduction, and so on.
