# Supplementary material for: Unusual bromine enrichment in the gastric mill and setae of the hadal amphipod Hirondellea gigas
Source: PLoS One. 2022 Aug 4;17(8):e0272032. doi: 10.1371/journal.pone.0272032 (PMC9352070; doi:10.1371/journal.pone.0272032)
Supplement: Attachment — Submitted filename: PONE-D-22-02007_R1_ReReview Text with Comments_Edits.pdf [file pone.0272032.s019.pdf]

PLOS ONE

Unusual bromine enrichment in the gastric mill and setae of the hadal amphipod Hirondellea gigas

--Manuscript Draft--

**Manuscript Number:**	PONE-D-22-02007R1
**Article Type:**	Research Article
**Full Title:**	Unusual bromine enrichment in the gastric mill and setae of the hadal amphipod Hirondellea gigas
**Short Title:**	Bromine enrichment in the gastric mill and setae of Hirondellea gigas
**Corresponding Author:**	Satoshi Okada<br>Japan Agency for Marine-Earth Science and Technology: Kaiyo Kenkyu Kaihatsu Kiko<br>Yokosuka, Kanagawa JAPAN
**Keywords:**	amphipod; Hirondellea gigas; scanning electron microscopy; artefact; bromine; carbonate compensation depth; gastric mill
**Abstract:**	The hadal amphipod Hirondellea gigas is an emblematic animal of the Pacific trenches, and has a number of special adaptations to thrive in this 'extreme' environment, which includes including the deepest part of the Earth's ocean. One such adaptation that has been suggested is the presence of an 'aluminum gel shield' on the surface of its body in order to prevent the dissolution of calcitic exoskeleton below the carbonate compensation depth. However, this has not been investigated under experimental conditions that sufficiently prevent aluminum artefacts, and the possibility of other elements with similar characteristic X-ray energy as aluminum (such as bromine) has not been considered. Here, we show with new electron microscopy data gathered under optimized conditions to minimize aluminum artefacts that H. gigas actually does not have an aluminum shield – instead many parts of its body are enriched in bromine, particularly gastric ossicles and setae. Results from elemental analyses pointed to the use of calcite partially substituted with magnesium by H. gigas in its exoskeleton, in order to suppress dissolution. Our results exemplify the necessity of careful sample preparation and analysis of the signals in energy-dispersive X-ray spectroscopic analysis, and the importance analyses at different electron energies.
**Order of Authors:**	Satoshi Okada
	Chong Chen
	Hiromi Kayama Watanabe
	Noriyuki Isobe
	Ken Takai
**Response to Reviewers:**	Thank you very much for Reviewer 2's critical comments, which were extremely helpful to revise our manuscript. The manuscript was revised accordingly, and the point-to-point response is shown in the separate file.
**Additional Information:**	
**Question**	**Response**
**Financial Disclosure**<br><br>Enter a financial disclosure statement that describes the sources of funding for the work included in this submission. Review the submission guidelines for detailed requirements. View published research articles from *PLOS ONE* for specific examples.	S.O., [Grant-in-Aid for Young Scientists Grant Number JP19K15379], Japan Society for the Promotion of Science (JSPS), https://www.jsps.go.jp/; C.C. and H.K.W., [Grant-in-Aid for Scientific Research Grant Number 18K06401], JSPS. The funders had no role in study design, data collection and analysis, decision to publish, or preparation of the manuscript.

Powered by Editorial Manager® and ProduXion Manager® from Aries Systems Corporation

This statement is required for submission and **will appear in the published article** if the submission is accepted. Please make sure it is accurate.<br><br>**Unfunded studies**<br>Enter: *The author(s) received no specific funding for this work.*<br><br>**Funded studies**<br>Enter a statement with the following details:<br>• Initials of the authors who received each award<br>• Grant numbers awarded to each author<br>• The full name of each funder<br>• URL of each funder website<br>• Did the sponsors or funders play any role in the study design, data collection and analysis, decision to publish, or preparation of the manuscript?<br>• **NO** - Include this sentence at the end of your statement: *The funders had no role in study design, data collection and analysis, decision to publish, or preparation of the manuscript.*<br>• **YES** - Specify the role(s) played.<br><br>* typeset	
**Competing Interests**<br><br>Use the instructions below to enter a competing interest statement for this submission. On behalf of all authors, disclose any competing interests that could be perceived to bias this work—acknowledging all financial support and any other relevant financial or non-financial competing interests.<br><br>This statement is required for submission and **will appear in the published article** if the submission is accepted. Please make sure it is accurate and that any funding sources listed in your Funding Information later in the submission form are also declared in your Financial Disclosure statement.<br><br>View published research articles from *PLOS ONE* for specific examples.	The authors have declared that no competing interests exist.

**NO authors have competing interests**<br><br>Enter: *The authors have declared that no competing interests exist.*<br><br>**Authors with competing interests**<br><br>Enter competing interest details beginning with this statement:<br><br>*I have read the journal's policy and the authors of this manuscript have the following competing interests: [insert competing interests here]*<br><br>* typeset	
**Ethics Statement**<br><br>Enter an ethics statement for this submission. This statement is required if the study involved:<br><br>• Human participants<br>• Human specimens or tissue<br>• Vertebrate animals or cephalopods<br>• Vertebrate embryos or tissues<br>• Field research<br><br>Write "N/A" if the submission does not require an ethics statement.<br><br>General guidance is provided below. Consult the submission guidelines for detailed instructions. **Make sure that all information entered here is included in the Methods section of the manuscript.**	N/A

Powered by Editorial Manager® and ProduXion Manager® from Aries Systems Corporation

**Format for specific study types**<br><br>**Human Subject Research (involving human participants and/or tissue)**<br>• Give the name of the institutional review board or ethics committee that approved the study<br>• Include the approval number and/or a statement indicating approval of this research<br>• Indicate the form of consent obtained (written/oral) or the reason that consent was not obtained (e.g. the data were analyzed anonymously)<br><br>**Animal Research (involving vertebrate animals, embryos or tissues)**<br>• Provide the name of the Institutional Animal Care and Use Committee (IACUC) or other relevant ethics board that reviewed the study protocol, and indicate whether they approved this research or granted a formal waiver of ethical approval<br>• Include an approval number if one was obtained<br>• If the study involved *non-human primates*, add *additional details* about animal welfare and steps taken to ameliorate suffering<br>• If anesthesia, euthanasia, or any kind of animal sacrifice is part of the study, include briefly which substances and/or methods were applied<br><br>**Field Research**<br><br>Include the following details if this study involves the collection of plant, animal, or other materials from a natural setting:<br>• Field permit number<br>• Name of the institution or relevant body that granted permission	
**Data Availability**<br><br>Authors are required to make all data underlying the findings described fully available, without restriction, and from the time of publication. PLOS allows rare exceptions to address legal and ethical concerns. See the PLOS Data Policy and FAQ for detailed information.	Yes - all data are fully available without restriction

A Data Availability Statement describing where the data can be found is required at submission. Your answers to this question constitute the Data Availability Statement and **will be published in the article**, if accepted.<br><br>**Important:** Stating 'data available on request from the author' is not sufficient. If your data are only available upon request, select 'No' for the first question and explain your exceptional situation in the text box.<br><br>Do the authors confirm that all data underlying the findings described in their manuscript are fully available without restriction?	
**Describe where the data may be found in full sentences. If you are copying our sample text, replace any instances of XXX with the appropriate details.**<br><br>• If the data are **held or will be held in a public repository**, include URLs, accession numbers or DOIs. If this information will only be available after acceptance, indicate this by ticking the box below. For example: *All XXX files are available from the XXX database (accession number(s) XXX, XXX.).*<br>• If the data are all contained **within the manuscript and/or Supporting Information files**, enter the following: *All relevant data are within the manuscript and its Supporting Information files.*<br>• If neither of these applies but you are able to provide **details of access elsewhere**, with or without limitations, please do so. For example:<br><br>*Data cannot be shared publicly because of [XXX]. Data are available from the XXX Institutional Data Access / Ethics Committee (contact via XXX) for researchers who meet the criteria for access to confidential data.*<br><br>*The data underlying the results presented in the study are available from (include the name of the third party*	All relevant data are within the manuscript and its Supporting Information files.

Powered by Editorial Manager® and ProduXion Manager® from Aries Systems Corporation

and contact information or URL).<br>• This text is appropriate if the data are owned by a third party and authors do not have permission to share the data.<br><br>* typeset	
Additional data availability information:	

Powered by Editorial Manager® and ProduXion Manager® from Aries Systems Corporation

Manuscript
Click here to access/download;Manuscript;okada_manuscript_rev_notrack.d

Unusual bromine enrichment in the gastric mill and setae of the hadal amphipod *Hirondellea gigas*

Satoshi Okada[1*], Chong Chen[1], Hiromi Kayama Watanabe[1], Noriyuki Isobe[2], Ken Takai[1]

[1] Institute for Extra-cutting-edge Science and Technology Avant-garde Research (X-STAR), Japan Agency for Marine-Earth Science and Technology (JAMSTEC), 2-15 Natsushima-cho, Yokosuka, Kanagawa 237-0061, Japan

[2] Biogeochemistry Research Center, Research Institute for Marine Resources Utilization (MRU), Japan Agency for Marine-Earth Science and Technology (JAMSTEC), 2-15 Natsushima-cho, Yokosuka, Kanagawa 237-0061, Japan

* Corresponding author

E-mail: okadasa@jamstec.go.jp

Abstract

The hadal amphipod *Hirondellea gigas* is an emblematic animal of the Pacific trenches, and has a number of special adaptations to thrive in this ‘extreme’ environment, which includes inclu[illegible]g the deepest part of the Earth’s ocean. One such adaptation that has been suggested is the presence of an ‘aluminum gel shield’ on the surface of its body in order to prevent the dissolution of calcitic exoskeleton below the carbonate compensation depth. However, this has not been investigated under experimental conditions that sufficiently prevent aluminum artefacts, and the possibility of other elements with similar characteristic X-ray energy as aluminum (such as bromine) has not been considered. Here, we show with new electron microscopy data gathered under optimized conditions to minimize aluminum artefacts that *H. gigas* actually does not have an aluminum shield – instead many parts of its body are enriched in bromine, particularly gastric ossicles and setae. Results from elemental analyses pointed to the use of calcite partially substituted with magnesium by *H. gigas* in its exoskeleton, in order to suppress dissolution. Our results exemplify the necessity of careful sample preparation and analysis of the signals in energy-dispersive X-ray spectroscopic analysis, and the importance [illegible]alyses at different electron energies.

Introduction

Many animal groups have evolved hard parts for skeletal support (e.g. vertebrate bones), protection (e.g. molluscan shells), and feeding (e.g. mouth parts and teeth), among other functions – and often for multiple purposes. These hard parts are composed of either inorganic minerals, such as calcite, aragonite, and hydroxyapatite, or organic polymers, such as chitin, or a mixture of both. Inorganic biomineralized parts exposed to the outside world are often coated with organic matrices, such as molluscan shells covered by a layer of periostracum made out of a combination of chitin and proteins.[1] In some cases, such as the marine polychaete worm *Nereis virens*, heavy transition metals and halogens are utilized to coat the mineralized tissues.[2,3] These external layers often play roles in the secretion of the biominerals, and they can also reinforce the structure and prevent the deterioration of the underlying mineral layer. For example, bivalves living deeper than the carbonate compensation depth (CCD; typically ~4500 m), where calcium carbonate dissolves quickly, use their periostracum to buffer against the natural shell dissolution.[4]

Crustaceans are among the most successful invertebrates in marine environments and use hard parts for both protection in the form of calcified exoskeletons and feeding in the form of gastric mills. Their exoskeletons are mainly composed of chitinous biopolymers hardened through sclerotization, the cross-linking of protein components. [5,6] To achieve further mechanical strength and/or adaptive functionality, the exoskeletons are often additionally impregnated with other materials. The most widespread example is

amorphous calcium carbonate and calcium phosphate.[5–7] Though most crustaceans with calcified exoskeleton predominantly use calcium carbonate, some such as barnacles in the genus *Ibla* chiefly utilize calcium phosphate.[8,9] Phosphate being more common in mandibles.[10] Calcium phosphate is often used to strengthen the mandible, such as in the crayfish which forms fluoroapatite as a crown on its mandible.[11,12] Some crustaceans such as woodlices, use crystalline magnesium calcite where a part of calcium is substituted with magnesium.[13] Furthermore, a number of crustaceans exhibit further coatings outside the exoskeleton. For examples barnacles where the hardened plates are overlaid with cuticular and epicuticular layers,[8] deep-sea ostracods which cover the mineralized layer with epidermis to prevent mineral dissolution even below the CCD,[14,15] and crabs such as *Pachygrapsus crassipes* possesses bromine-enriched coating on their claw tips that increases the fracture-resistance.[16,17]

The hadal amphipod *Hirondellea gigas* is one of the most numerically dominant crustacean and macrofauna at the deepest areas of the ocean, including the Challenger Deep in the Mariana Trench (10,994 m).[18] As special adaptations to this extreme hadal environment, this species possesses unique cellulase and is alleged to digest polysaccharides from sunken plant matter using a unique cellulase.[18,19] Perhaps most strikingly, it is said to secrete aluminum to the outside its exoskeleton to prevent the dissolution of calcium-based minerals, dubbed the ‘aluminum shield’.[20] Based on the combination of energy-dispersive X-ray analysis (EDS) under scanning electron microscopy

(SEM), and aluminum extraction experiments from the sediment of Challenger Deep, Kobayashi et al.[20] expected that the 'aluminum shield' of *H. gigas* would be in gel form. However, their results were inconclusive and ambiguous due to the localization pattern of aluminum, where the signal intensity of aluminum was especially high only at the edge of the samples close to the aluminum stubs most prone to artefacts. [illegible]thermore, samples were not covered with conductive coatings were not performed for imaging, despite observations being undertaken in the aluminum-rich environment that is the SEM chamber. In addition, EDS spectra under a scanning transmission electron microscope (STEM) of the exoskeleton did not provide enough information to support the existence of aluminum and its detailed nanostructure.[20] In ord[illegible]o confirm the presence of the 'aluminum shield' of *H. gigas* and to further substantiate its distribution and potential function, we designed a more careful experimental design to exclude aluminum artefacts.

There are two possibilities where aluminum artefacts may be observed: 1) backscattered electrons (BSE) from the observed specimen hitting the chamber made of aluminum alloy to generate secondary electrons (called SE3) as well as aluminum X-ray,[21] overlapping of other elements that have similar characteristic X-ray energy signatures to that of aluminum (Al K-line, 1.486 keV) and which are inseparable using conventional EDS whose energy resolution b[illegible]g ~130 eV. These elements include bromine (Br L-line, 1.480 keV), thulium (Tm M-line, 1.462 keV), and ytterbium (Yb M-line, 1.521 keV),[22] the latter two i[illegible]nused in animals.[23,24] Here, we report our findings from detailed electron

microscopy observations of *H. gigas*, revealing the absence of aluminum on the exoskeleton and instead the accumulation of bromine on hard tissues as the sclerotized spines of its gastric mill components, a rare example of preferentially secreted bromine in the hard parts of animals.

Materials & Methods

Materials

All reagents were purchased either from Nacalai tesque Inc., Fuji Film Wako Pure Chemical Co., or Kanto Chemical Co., Inc. and used as received. Deionized water was supplied from Merck Milli-Q Integral 5.

Amphipod samples

Hirondellea gigas individuals were collected using a bait trap attached to a Free Fall Camera System at Izu-Ogasawara Trench near the Boso Triple Junction (34°20.4296′N 142°0.6006′E, Fig 1a), depth 9218 m on September 4, 2019, on R/V Yokosuka cruise YK19-11. Over 200 individuals (Fig 1b) were collected, of which three individuals were used for this study. The amphipods were immediately immersed in slush nitrogen (ca. –210 °C) for more than a minute to rapidly freeze the exoskeleton and stored at –80 °C during the cruise, then transferred to liquid nitrogen storage (actual storage temperature <–160 °C) after cruise.

Fig 1. Sampling of *Hirondellea gigas*. a) Sampling location in a red dot. b) *H. gigas* collected in the bait trap. More than 200 individuals are collected. c) A micrographic montage of *H. gigas* under stereomicroscope.

Electron microscope instruments

SEM and scanning transmission electron microscopic (STEM) imaging were performed on a Helios G4 UX (Thermo Fisher Scientific), an Octane Elite Super energy dispersive X-ray analysis (EDS) detector (Octane Elite Super, AMETEK) and a cryogenic stage with preparation chamber (PP3010T, Quorum). Transmission electron microscopy (TEM) was performed on a Tecnai G2 20 (Thermo Fisher Scientific, operated at 200 kV) equipped with a bottom-mounted 2k × 2k Eagle Charge-Coupled Device camera (Eagle, Thermo Fisher Scientific) and RTEM-S 61700NE an EDS detector (RTEM-S 61700NE, AMETEK). Electron probe microanalysis (EPMA) was performed on a JXA-8500 (JEOL Ltd.) equipped with five wavelength dispersive X-ray detectors (WDS, XM-86030 and XM-86010, JEOL Ltd.).

Sample preparation and semi-thin section imaging

Frozen amphipods were dehydrated in acetone at –80 °C for three days and gradually warmed to room temperature. The dehydrated amphipod was immersed into QY-1 (Ohkenshoji Inc.), QY-1/epoxy mixture, then with an epoxy resin (TAAB), trimmed

into pieces using laser blades, and embedded in the epoxy resin following the Luft's method.[25] Transverse sections of the head part were taken from anterior to posterior into 1 μm-thick semi-thin sections using an ultramicrotome (Ultracut S or EM UC7, Leica) with a diamond knife (45°, Diatome). Sections were collected on a glass slide covered with Kapton tape (Nitto Denko Co., P-224, 64 μm thickness) using a nickel loop. The slide was coated with carbon for conductivity using CADE (Meiwaforsis Inc.). Semi-thin sections of pereonite were prepared in the same manner.

Semi-thin sections were subjected to SEM/EDS analysis operated at 1 kV for morphological imaging, and at 10 kV for elemental analysis and mapping. Chlorine was observed uniformly in the epoxy resin as its contaminant, and carbon coating was performed for conductivity on the polyimide-based substrate. However, the localization of these two elements was not discussed in detail.

Elemental analysis at high energy resolution was performed on an EPMA operated at 20 kV and beam current of 12 nA. The elemental colocalization in the EPMA map was quantified using cross-correlation image analysis.[26] The cross-correlation factor γ between two elemental maps a and b was calculated as

$$\gamma(a,b) = \frac{\sum_{ij}\{[I_a(r_{ij}) - \bar{I}_a] \cdot [I_b(r_{ij}) - \bar{I}_b]\}}{\sqrt{\sum_{ij}[I_a(r_{ij}) - \bar{I}_a]^2} \cdot \sqrt{\sum_{ij}[I_b(r_{ij}) - \bar{I}_b]^2}} \quad (1)$$

where $I_x(r_{ij})$ denotes the X-ray intensity of map x at the pixel position (I, j), and $\bar{I}_x$ is the averaged intensity of map x over the image. The cross-correlation calculation and EPMA elemental mapping were implemented in Python 3.9.1.

Microscopic observation and image analysis of a dissected amphipod

Another frozen individual of *H. gigas* (total length 26.0 mm, telson length 5.3 mm) was defrosted and dissected for three-dimensional structural investigation under a stereomicroscope (SZX7, Olympus) and photos were taken using an Olympus E-M5 Mark III DSLR camera (Fig 1c). The amphipod was dissected into different parts, with observations focusing on: mandible, maxilla, maxilliped, antennae, gastric mill (pterocardiac ossicles in the cardiac stomach), pereonites, pleopod, uropod, and coxal plates. Dissected samples were placed within Eppendorf tubes (0.2 mL), dehydrated by an increasing series of ethanol (50, 60, 70, 70, 70, 80, 90, 99, 100, 100, 100%, with more than 10 minutes at each step), then substituted with *tert*-butanol, frozen at –20 °C, and dried under vacuum (<1 × 10^{-1} Pa) to obtain dried specimens, which were then mounted on ~~a~~ Kapton tape on a glass slide using ~~a~~ double-sided carbon adhesive tape. The mounted samples were coated with carbon for conductivity, and analyzed in SEM/EDS operated at 1 kV for morphological imaging and 10 or 20 kV for EDS analyses.

X-ray measurement

Dried pereonites were cut into flat pieces and mounted on an X-ray diffractometer (X'Pert PRO, PANalytical) with a Cu radiation source (Kα = 1.5418 Å) at 45 kV and 40 mA. The diffractogram from 10° to 60° was acquired at 0.0167° steps at the scan speed of 0.07°/s. The 16 spectra were integrated, and the mineral assignment was performed on the software (HighScore 3.0.5, PANalytical).

Results

Elemental distribution and composition of *H. gigas*

To observe the potential aluminum layer in the exoskeleton of *Hirondellea gigas*, the elemental composition was investigated by SEM/EDS of thin sections to avoid artificial aluminum signals caused by the substrate and non-flat surfaces (Figs 2, S1 Fig). EDS maps of the pereonite and telson showed no evidence of aluminum, while strong signals of carbon, oxygen, phosphorous, and calcium as well as a weak signal of sulfur were observed in the area where bright contrast was observed in the SEM images (Figs 2a, S1a Fig). This result agrees with the previous observation[20] that the exoskeleton is composed of calcium carbonate and proteinaceous organic materials. Other elements, such as sodium, aluminum, and silicon were within the background levels of the continuous X-ray (bremsstrahlung), while chlorine was from the epoxy resin. The EDS maps showed uniform existence of these elements, indicating the compositional homogeneity of the exoskeleton. Soft parts within the exoskeleton were composed of organic materials, and the signal of chlorine corresponds

to the epoxy resin. The intensities of the aluminum-like signal (as aluminum overlaps with that of bromine, thulium, and ytterbium) was similar to that of the background signals, indicating that this signal was below the detection limit of the imaging condition used here.

Fig 2. EDS maps of semi-thin sections of *H. gigas*. a) transverse section through a subsample of pereonite, b) transverse section of the cephalic region showing the gastric mill, c) magnified images of the gastric mill, the red rectangle area in (b). Images from the left are SEM images at (a,b) 1 kV and (c) 10 kV, total X-ray counts (grayscale), phosphorous (green), calcium (blue), and aluminum-like signals around 1.48 keV (silver). EDS spectra were acquired at 10 kV, thus the K-line of bromine was not observed.

To confirm that the mineral components of the pereonites of *H. gigas* are the same as previously observed (i.e. mainly calcitic),[20] XRD measurements were performed using the dissected dried pereonite. Nine of ten peaks were assigned to that of calcite (S2 Fig). The residual peaks at 19.7° and 26.7° were explained by α-chitin, which is ubiquitous in crustacean exoskeletons.[27,28] This result combined with EDS analysis confirmed that the exoskeleton of *H. gigas* is mainly made of calcite and organic matrices, including phosphorous.

The elemental composition of the semi-thin sections through the gastric mill was different from that of pereonite (Figs 2b and2c, S1b and S1c Figs). The tissues surrounding

the gastric mill showed similar elemental content to that of the pereonite. On the other hand, a strikingly different composition was found on the gastric mill itself (Fig 2c)[29] which appeared to form a bilayered structure with an inner layer composed of carbon, nitrogen, oxygen, sodium, magnesium, and phosphorous, while the outer layer was composed of carbon, nitrogen, oxygen, sodium, calcium, and [illegible]uminum-like signal at 1.48 keV. Magnified EDS maps showed the thickness of the outer layer to be ~1 μm.

EPMA analysis was also performed using similar sections of *H. gigas* (Fig 3a)to correctly assign the X-ray signals at 1.48 keV. Qualitative WDS spectrum of the outer layer of the gastric mill showed two peaks around 1.48 keV: 1.481 and 1.527 keV (Fig 3b top). Aluminum and bromine are the two possible candidates for these peaks: L-line of bromine appears at 1.293 and 1.480 kV while K-line of aluminum appears at 1.486 keV[22]. The intensity around 1.48 keV can therefore be explained without aluminum being present, indicating that the aluminum-like signals we have observed could in fact be bromine (S3 Fig). The presence of bromine was further supported by another signal at 11.92 keV [illegible]as also observed, corresponding to the K-line of bromine (Fig 3b bottom). EPMA maps showed directional bromine localization toward the side exposed to seawater, similar to that observed in the EDS maps.

Fig 3. X-ray spectra and elemental maps of a semi-thin section of the gastric mill in *Hirondellea gigas*. a) SEM image of the mapped area. b) Point analysis at the red point in

(a). c) WDS elemental maps of the orange rectangle in (a). Note that Al K contrast is significantly weaker than that of Br K.

The elemental colocalization was further confirmed by calculating cross-correlation factor of the whole image (S1 Table).[26] A high ~~High~~ correlation was observed between Ca-O-F-Mg-P, with correlation values ranging from 0.29 to 0.70. On the other hand, the correlation values of bromine against calcium-colocalizing elements were less than 0.1, indicating that the bromine layer is separated from the calcium-based minerals. Quantitative X-ray analysis showed the absence of calcium on the bromine-rich outer layer (S3c Fig). The low concentration of bromine and the coexistence of fluorine and magnesium were also supported by the quantitative analysis (S3 Fig).

Similarly, EPMA maps of the pereonite were taken to explore elements of the exoskeleton (Fig 4, S2 Table). Cross-correlation values indicated the high colocalization of Ca-O-Mg-P with correlation values ranging from 0.15 to 0.70. The correlation of calcium-colocalizing minerals was small due to low X-ray counts. The correlation of bromine to calcium-colocalizing minerals was less than 0.1 except for oxygen, denying strong bromine colocalization. Quantitative analysis clearly showed the absence of either bromine or aluminum (S3b and S3c Figs).

Fig 4. SEM images and elemental maps of a semi-thin section of the pereonite in *Hirondellea gigas*. a) Low-magnification SEM image. b) High-magnification SEM image of the red rectangle in (a). c) WDS elemental maps of the orange rectangle in (b).

EDS investigation of the dissected specimen

To further explore the localization of bromine in *Hirondellea gigas*, a dissected individual was freeze-dried and observed under SEM/EDS. EDS maps showed that the hard tissues in ~~the~~ *H. gigas* are mainly composed of carbon, nitrogen, oxygen, phosphorous, and calcium as major elements, and fluorine, sodium, magnesium, chlorine, and bromine as minor elements.

Since bromine accumulation was detected on the sclerotized spines of the gastric mill by EPMA, we first examined the gastric mill. The gastric mill exhibited several large, sclerotized spines and numerous fine filter setae, the latter not well observed in thin sections (Fig 5, S4a and S4b Figs). The dimensions of the spines were found to be typically 100 μm in length and 15–25 μm in diameter (Figs 5a and 5b, S4c and S4e Figs). Fine filter setae around the sclerotized spines were simple and covered by adsorbents, presumably mucus (Figs 5c, S4d and S4f Figs). Elemental maps of Br K indicated an accumulation of bromine on the spines (Fig 5d, S3 Table). Line profile analysis of the Br K line showed that the bromine intensity was 12–13 at the root and 15 at the tip, indicating preferential bromine accumulation at the tip (Fig 5e). The presence of weak calcium signals, combined with

EPMA results Fig 3), point to a bromine-rich and calcium-poor organic layer coating a calcium-rich inorganic layer. Although the intensity was lower, bromine accumulation was also observed on the filter setae (S4h Fig). The pterocardiac ossicle itself at the base of the spines is made of carbon, oxygen, magnesium, phosphorous, and calcium, while bromine was not observed in significant concentrations (S4h Fig).

Fig 5. SEM/EDS images of the gastric mill of *Hirondellea gigas*. a) low-magnification SEM image, b) magnified image of the spine, yellow rectangle in part (a), c) magnified image of the filter setae, orange rectangle in part (a), d) EDS elemental mapping and sum spectrum of the red rectangle in part (a). e) Line profile of the Br K intensity in EDS map along with the longitudinal axis of the ossicle spines. Profiles were shifted to the intensity direction for visibility, and average ± standard deviation of the intensity of 60 μm from root and 50 μm from the tip are shown in green and blue bars, respectively. The line profile was measured with the line width of 10 μm.

Because fine setae on the gastric mill also accumulated bromine, mouth parts around the gastric mill with fine setae, namely maxilla 2, maxilliped, and mandible, were analyzed by SEM/EDS (Fig 6, S5–S7 Figs). Bromine accumulation was also observed on the setae of these mouth parts, specifically the robust setae. Bromine accumulation was clearest on maxilla 2 (Figs 6a and 6b, S5 Fig). The maxilla itself was mainly composed of calcium

carbonate together with magnesium and phosphorous, and the bristles were found on the surface, where they are exposed to seawater (Fig 6c, S5e and S5f Figs). Three types of setae were present on maxilla 2 (Figs 6a and 6b). One was simple robust setae with a diameter of 10–20 μm and a length of 150–350 μm, occurring on the distal part of maxilla 2 (S5b right Fig); a second type of robust setae was similar in size but plumose with many fine setae (diameter of 0.6–2.5 μm and length of 35–50 μm, Fig S5b bottom); a third type was fine, setulose setae 0.5–1.5 μm in diameter with a length of 50–150 μm (S5b top, S6c, and S6d Figs). EDS maps showed that bromine was accumulated on both types of robust setae with carbon, nitrogen, oxygen, and sulfur, but not on the finer, setulose setae which were rich in phosphorous and calcium (Fig 6c, S3 Table). Similarly, selective bromine accumulation on the robust setae was found in both the maxilliped and the mandible (S6 and S7 Figs, S3 Table). Since the tissues were not washed excessively in order to prevent damage to potentially fragile setae, sodium chloride particles were observed between the setae of maxilliped, and bromine was not observed on the particles (S6c and S6e Figs).

Fig 6. SEM/EDS images of maxilla 2 of *Hirondellea gigas*. a) low-magnification SEM image, b) magnified image of the yellow rectangle in part (a), c) EDS elemental mapping and sum spectrum of the red rectangle in part (a).

These accumulations of bromine raised the possibility that bromine is widely present on setae across the whole amphipod, and we therefore proceeded to investigate other parts of the body where setae are present, including antenna, pleopods, and uropods. Antenna, fine parts most close to the mouth, (Fig 7, S8a Fig) also contained the presence of bromine specifically on the setae extending from the distal parts of each article. These setae were simple and fine, 50–80 μm in length and 1–3 μm in diameter (Figs 7a and 7b, S8b and S8d Figs). Setae on the distal end of article 1 of the antennae are, however, barbed and have short barbs projecting out laterally (S8c and S8e Figs). Elemental maps and point analysis revealed bromine accumulation on the setae, free of magnesium, phosphorous, and calcium that were found on the articles themselves (Fig 7c, S3 Table). The setae were characterized by high carbon, nitrogen, oxygen, and sulfur contents, while magnesium and phosphorous were less significant.

Fig 7. SEM/EDS images of an antenna of *Hirondellea gigas*. a) low-magnification SEM image, b) magnified image of the yellow rectangle in part (a), c) EDS elemental mapping and sum spectrum of the red rectangle in part (a).

Microscopic observations of pleopods revealed bromine accumulation on robust setae as well as the outer surface of the pleopods themselves (Fig 8, S9 and S10 Figs). Two types of setae were present, with the robust setae being simple and approximately 4-5 μm

in diameter, and elongate fine setae being approximately 0.3-1.2 μm in diameter and about 3 mm in length (Figs 8a and 8b, S9b and S9c Figs). Bromine was found to be accumulated only on the robust setae (Fig 8c, S9d Fig). Unlike other parts of the body, some bromine accumulation was also detected on the surface of the pleopods (Figs 8c, S10 Fig, S3 Table).

Fig 8. SEM/EDS images of a pleopod of *Hirondellea gigas*. a) low-magnification SEM image, b) magnified image of the yellow rectangle in part (a), c) EDS elemental mapping and sum spectrum of the red rectangle in part (a).

Uropods possess short distolateral robust setae (Fig 9, S11a Fig), and we also found bromine accumulating on the setae, which were approximately 10–15 μm in diameter and 80–100 μm in length (Fig 9b, S11b and S11c Figs). The overall surface of the uropods including the setae lacked bromine accumulation, unlike in the pleopods (Fig 9c, S11d Fig, S3 Table).

Fig 9. SEM images and EDS maps of dissected dry uropod of *Hirondellea gigas*. a) Low-magnification image. b) High-magnification image of the yellow square in (a). c) Sum spectrum and EDS maps of the red square in (a). The sum spectra at 10–15 kV were magnified and displayed in the inset above with the same energy scale for clarity.

Since EPMA investigation showed an absence of bromine on the pereonite, suggesting that bromine could be absent on the surface of the dorsal armature of *H. gigas*. We therefore performed SEM/EDS analysis on the coxal plates and pereonites (Fig 10, S12a Fig). Both coxal plates and pereonites were composed of hexagonal elements approximately 30 μm in size, which were more clearly visible on the coxal plates (Fig 10b, S12c Fig). High-magnification images of the dorsal armature revealed numerous bead-like structures arranged in nearly parallel lines transversing the body, with the typical spacing between the lines being 669 ± 184 nm (*N* = 36, S12d Fig). Elemental mapping showed a lack of any specific localization of bromine related to the surface structure in these parts of the exoskeleton, as was also indicated in area analyses (Fig 8c, S12e Fig, S3 Table); though the sum spectra indicated small amounts of bromine.

Fig 10. SEM/EDS images of a coxal plate of *Hirondellea gigas*. a) low-magnification SEM image, b) magnified image of the yellow rectangle in part (a), c) EDS elemental mapping and sum spectrum of the red rectangle in part (a).

Discussion

Elemental analysis using electron microscopy

We took several precautions during sample preparation to avoid artificial X-ray signals from aluminum outside the sample, which is a very common element and contaminant in electron microscopy.[23] The use of Kapton/carbon reduces the emission of BSE from the substrate, preventing secondary electrons from the SEM chamber (SE3) as well as X-rays from the chamber made of aluminum alloy.[21] The amphipod tissue was mounted as carbon-coated flat semi-thin sections (unlike the bulk specimen in ref. [20]) due to three benefits in X-ray analysis. Firstly, the spatial resolution of a semi-thin section is higher than that of a bulk specimen because X-ray generation distant from the irradiated position by electrons scattered inside the sample is suppressed. Secondly, the charge-up of the thin section is minimized, as primary electrons irradiated to the thin section are not retained in the sample, but instead penetrate through to the underlying layer, where they are neutralized by the conductive coatings.[30] In the case of bulk samples with an insulative interior, as is the case with biological materials, accumulated charges bend the electron trajectory to decrease spatial resolution. Finally, using semi-thin sections ensure the electrons and X-rays are emitted homogeneously from a flat surface, which is desirable for quantitative analysis. Surface roughness causes shading of X-rays to decrease signal intensities of low-energy X-rays.[31] Without these precautions, total X-ray counts from each position do not necessarily reflect the sample's true elemental composition. We used the counts per second (cps) images, corresponding to total X-ray counts from each pixel, as our criteria to distinguish whether the signal originated from the sample or bremsstrahlung.

After performing the analyses with these precautions, our results showed that what was previously thought to be aluminum signals from *Hirondellea gigas*[20] was most likely bromine. This misassignment of elements can be traced back to issues with the sample preparation and selection of electron microscopic imaging conditions in Kobayashi *et al*.[20] Although high-resolution imaging can be performed without any coatings by using low-landing voltage techniques under low beam current,[32,33] EDS measurements typically require doubled voltage of maximum characteristic X-ray energy of analyzing elements. In this situation, a large beam current is required for X-ray emission sufficient for elemental mapping. The spectrum acquisition condition is prone to cause charge-up, as electrons can penetrate deep into the insulative organic specimen. In addition, internalized high-energy electrons can escape from the side of curved surfaces, resulting in enhanced counts of low-angle BSE – thus the total intensity of X-rays from the SEM chamber and substrate at the sample edge, which corresponds to the previous results where aluminum signals were mostly detected on the sample edges (Fig 1 in ref. [20]). The use of aluminum stubs and aluminum-reinforced double-sided carbon tape, as in ref. [20], is not recommended for aluminum detection for these reasons. Silicon is also an element to be used with caution, because the EDS detector window is made of silicon, which can be excited either by BSE or incoming high-energy X-rays, the latter of which is known as the escape peak.

One criterion to distinguish whether the EDS signals in map images truly originate from elemental localization or are artefacts, is to compare the image with total X-ray counts

at each pixel, or counts per second (cps) images. Uncorrected X-ray maps display X-ray intensities of a given X-ray energy range at each position, including bremsstrahlung X-ray that are in correlation with the total emitted X-rays. When the relative intensities of X-ray maps and the cps map of the area concerned are similar in ratio, the element localization is in doubt. Note that conventional X-ray corrections, such as ZAF correction using atom number (Z), X-ray absorption (A) and fluorescence excitation (F) are not necessarily accurate for EDS analysis of non-flat specimens. Increasing the beam energy is another method to distinguish among elements. In the particular case of the aluminum K-line (1.486 keV), the bromine L-line (1.480 keV) and M-lines of two lanthanides absent in animals (thulium and ytterbium)[24] can overlap with it at the energy resolution of EDS (typically ~130 eV). By using high-energy electron beams, the bromine K-line (11.92 keV) can be detected without overlapping with other elements. High-energy X-rays have lower intensities than low-energy ones, and the use of high-sensitivity detectors is desired. The use of semi-thin sections, comparison of X-ray maps and cps maps, irradiation of high-energy electron beam, and the use of the high-sensitivity detector in our analytical methods ensured that we were likely to avoid such artifact pitfalls.

In our measurements, sea salt sometimes appeared on the surface, which is an artefact of the drying process, because we did not wash the tissues thoroughly to prevent removal of fragile parts. However, sea salt only appeared as granules, not as surface coatings, judging from the amount of Na and Cl being far lower than that of Br on fine setae

(S4–S8 Figs). This indicates that salt did not affect the amount of bromine because the molar amount of bromine is less than 1% of that of sodium, the main content of the sea salt.

Origin of bromine and its biological implication

Bromine is the eighth most abundant element in the ocean after chlorine, sodium, magnesium, sulfur, calcium, potassium, and carbon.[34] Although the explicit amount of bromine in the Izu-Ogasawara Trench has not been reported,[35] the relative amount of bromine is expected to be similar to that of standard seawater. The accumulation of bromine could be a result of 1) solidification and concentration from sea salt during sample preparation, 2) exogenous adhesion on the amphipod's exoskeleton while the amphipod was still alive, or 3) active accumulation and secretion by the amphipod itself. We observed solidified sea salt on the setae of maxilliped but these were without bromine coexistence (S6c–S6e Figs), rejecting the bromine signals being results of artefacts from sample preparation. Exogenous adhesion is also considered to be unlikely since chlorine, which has similar chemical properties as a water-dissolved anion and being more abundant in seawater than bromine, was absent on the setae. Rather, it is more plausible to consider that bromine was secreted by the amphipod, most preferentially to the gastric mill but also to the robust setae on various parts of the body, as well as the surface of the pleopods.

Accumulation of bromine has been ~~also~~ found on other marine organisms, and bromine-rich aromatic small molecules have been isolated from marine organisms. For examples, the marine snail *Bolinus bandaris* (a historically important source of Tyrian purple)

secretes 6-bromo-3-indolinone derivatives,[36] the tripeptide barettin from the marine sponge *Geodia barretti* has dehydrogenated 6-bromotryptophan,[37] and marine bacteria such as *Pseudomonas bromoutilis* produces pentabrominated phenylpyrrole, which is an antibiotic compound.[38,39] Bromine is attached on aromatic rings in all cases. There are also examples of bromine being accumulated on the hard parts of marine organisms whose molecular structure has not been identified, such as the fish otolith containing bromine and chlorine colocalized as minor elements.[40] Such cases of bromine in hard parts are also known from at least two cases where the hard parts have predation or feeding-related functions. In the crab *Pachygrapsus crassipes*, bromine is enriched on the tips of its calcified claws, and mechanical tests have shown that this makes the tips less hard, yet more fracture-resistant.[16,17] Another case is the zinc-based jaws of the marine polychaete worm *Nereis virens* that also contain chlorine, manganese, and iodine; brominated amino acids, such as histidine, might bind to metals strongly to prevent dissolution. [2,3]

The hadal amphipod *H. gigas* is unique among these animals in that the bromine does not coexist with other halogens and it is poor in other transition metals within the 1-um thick organic coatings containing bromine. In *H. gigas*, bromine is expected to reinforce the gastric mill to crush food in the same manner as reinforcement occurs in the jaws of other animals. The localization of bromine on the sclerotized spines and the lack of it in the calcified ossicles itself is likely beneficial in that bromine renders the spines less susceptible to breaking, in order to maintain its sharpness and function in food sorting. The ossicles are

used for actual crushing, and as such it is beneficial for them to be calcified to increase the hardness. The same has been suggested for the function of the forceps-like, bromine-enriched, crab claw tips versus the rest of the claw used for crushing, which is calcified and lacks bromine.[16] The form of bromine in the gastric mill and setae remains unclear. The coexisting nitrogen and sulfur with bromine indicate the presence of amino acids, which may imply that the bromine is incorporated in aromatic amino acids, such as phenylalanine and tryptophan, in which case the bromine is most likely secreted by *H. gigas*. The function of bromine on the setae of *H. gigas* remains unclear at this point, although it may be secreted in a similar manner to the spines in the gastric ossicles.

Using calcite as the exoskeleton in depths deeper than the CCD results in dissolution, and how *H. gigas* can retain its calcite armature requires a reconsideration now that it has become clear that it does not possess an 'aluminum shield' like previously suggested.[20] EPMA showed the existence of magnesium in the calcite layer with the Mg/Ca ratio being around 2–20% (S2 Fig). The absence of chlorine in the dried specimen (S4–S12 Fig) indicates that the magnesium is not from the remaining seawater salt, but is within the exoskeleton. Water solubility of calcite decreases by slight substitution of calcium with magnesium, i.e. $(Ca,Mg)CO_3$,[41,42] and the substitution would partially prevent carbonate dissolution below the CCD. Phosphorous may be present as calcium phosphate, contributing to the stabilization of calcium carbonate minerals.[5,6,10]

Conclusion

In summary, we revealed the presence of bromine and magnesium in the hadal amphipod *Hirondellea gigas*, and showed that it does not actually possess an 'aluminum shield' as previously thought, through careful prevention of aluminum artefacts. Instead of the 'aluminum gel' shielding, the use of magnesium-substituted calcite may be the key for the amphipod to survive without demineralization below the CCD. A high concentrations of bromine were found on the sclerotized spines in the gastric mill, forming a 1 μm-thick coating layer on mineralized tissue with a relatively high concentration of nitrogen. The bromine-coating on the gastric mill spines is speculated to have the function of maintaining a sharp tip for food processing, similar to what has been suggested for crab claw tips. Our study serves as a reminder of the importance and necessity of appropriate sample preparation and careful analysis of the signals in EDS analyses, as well as the importance of analyzing samples at different electron energies when performing microscopic studies of 'elemental anatomy'.

Acknowledgements

The authors thank the Captain and crew of R/V *Yokosuka* during expeditions YK19-11 (cruise PI: Akinori Yabuki, JAMSTEC) for their great support of the scientific activity. All scientists on-board the expeditions are gratefully acknowledged for their tireless work, especially Heather Ritchie Parker who assisted with the preservation of the amphipod specimens. We also thank Kana Nagashima (JAMSTEC) for her kind offer for XRD analysis.

References

1. Meenakshi VR, Hare PE, Watabe N, Wilbur KM. The chemical composition of the periostracum of the molluscan shell. Comp Biochem Physiol. 1969;29: 611–620.

2. Broomell CC, Zok FW, Waite JH. Role of transition metals in sclerotization of biological tissue. Acta Biomater. 2008;4: 2045–2051.

3. Broomell CC, Mattoni MA, Zok FW, Waite JH. Critical role of zinc in hardening of Nereis jaws. J Exp Biol. 2006;209: 3219–3225.

4. Brandao SN, Hoppema M, Kamenev GM, Karanovic I, Riehl T, Tanaka H, et al. Review of Ostracoda (Crustacea) living below the Carbonate Compensation Depth and the deepest record of a calcified ostracod. Progress Oceanogr. 2019;178. doi:10.1016/j.pocean.2019.102144

5. Luquet G. Biomineralizations: insights and prospects from crustaceans. Zookeys. 2012;176: 103–121.

6. Bentov S, Abehsera S, Sagi A. The Mineralized Exoskeletons of Crustaceans. In: Cohen E, Moussian B, editors. Extracellular Composite Matrices in Arthropods. Cham: Springer; 2016. pp. 137–163.

7. Becker A, Ziegler A, Epple M. The mineral phase in the cuticles of two species of Crustacea consists of magnesium calcite, amorphous calcium carbonate, and amorphous calcium phosphate. Dalton Trans. 2005;0: 1814–1820.

8. Bourget E. Barnacle shells: Composition, structure and growth. In: Southward AJ, editor. Barnacle Biology. London: Taylor & Francis; 1987.

9. Lowenstam HA, Weiner S. Phosphatic shell plate of the barnacle Ibla (Cirripedia): A bone-likestructure. Proc Nat Acad Sci. 1992;89: 10573–10577.

10. Bentov S, Aflalo ED, Tynyakov J, Glazer L, Sagi A. Calcium phosphate mineralization is widely applied in crustacean mandibles. Sci Rep. 2016;6: 22118.

11. Tynyakov J, Bentov S, Abehsera S, Yehezkel G, Roth Z, Khalaila I, et al. A crayfish molar tooth protein with putative mineralized exoskeletal chitinous matrix properties. J Exp Biol. 2015;218: 3487–3498.

12. Bentov S, Zaslansky P, Al-Sawalmih A, Masic A, Fratzl P, Sagi A, et al. Enamel-like apatite crown covering amorphous mineral in a crayfish mandible. Nat Commun. 2012;3: 839.

13. Neues F, Hild S, Epple M, Marti O, Ziegler A. Amorphous and crystalline calcium carbonate distribution in the tergite cuticle of moulting Porcellio scaber (Isopoda, Crustacea). J Struct Biol. 2011;175: 10–20.

14. Keyser D, Walter R. Calcification in ostracodes. Revista Española de Micropaleontología. 2004;36: 1–11.

15. Brandão SN, Hoppema M, Kamenev GM, Karanovic I, Riehl T, Tanaka H, et al. Review of Ostracoda (Crustacea) living below the Carbonate Compensation Depth and the deepest record of a calcified ostracod. Prog Oceanogr. 2019;178: 102144.

16. Schofield RMS, Bailey J, Coon JJ, Devaraj A, Garrett RW, Goggans MS, et al. The homogenous alternative to biomineralization: Zn- and Mn-rich materials enable sharp organismal "tools" that reduce force requirements. Sci Rep. 2021;11: 17481.

17. Schofield RMS, Niedbala JC, Nesson MH, Tao Y, Shokes JE, Scott RA, et al. Br-rich tips of calcified crab claws are less hard but more fracture resistant: A comparison of mineralized and heavy-element biological materials. J Struct Biol. 2009;166: 272–287.

18. Kobayashi H, Hatada Y, Tsubouchi T, Nagahama T, Takami H. The Hadal Amphipod Hirondellea gigas Possessing a Unique Cellulase for Digesting Wooden Debris Buried in the Deepest Seafloor. PLoS ONE. 2012;7: e42727.

19. Kobayashi H, Nagahama T, Arai W, Sasagawa Y, Umeda M, Hayashi T, et al. Polysaccharide hydrolase of the hadal zone amphipods Hirondellea gigas. Biosci Biotechnology Biochem. 2018;82: 1123–1133.

20. Kobayashi H, Shimoshige H, Nakajima Y, Arai W, Takami H. An aluminum shield enables the amphipod Hirondellea gigas to inhabit deep-sea environments. PLoS ONE. 2019;14: e0206710.

21. Suri A, Pratt A, Tear S, Walker C, Kincal C, Kamber U, et al. Analysis and detection of low-energy electrons in scanning electron microscopes using a Bessel box electron energy analyser. J Elec Spec Rel Phen. 2020;241: 146823.

22. Bearden JA. X-ray wavelengths and x-ray atomic energy levels. Rev Mod Phys. 1967;39: 78-124.

23. Newbury DE. Mistakes encountered during automatic peak identification in low beam energy X-ray microanalysis. Scanning. 2007;29: 137-151. doi:10.1002/sca.20009

24. Cotruvo JA. The Chemistry of Lanthanides in Biology: Recent Discoveries, Emerging Principles, and Technological Applications. ACS Cent Sci. 2019;5: 1496-1506.

25. Luft JH. Improvements in epoxy resin embedding methods. J Biophys Biochem Cytol. 1961;9: 409-414.

26. Du K, Hochmeister K von, Phillipp F. Quantitative comparison of image contrast and pattern between experimental and simulated high-resolution transmission electron micrographs. Ultramicrosc. 2007;107: 281-292.

27. Cauchie H-M. Chitin production by arthropods in the hydrosphere. Hydrobiologia. 2002;470: 63-95.

28. Sugawara A, Nishimura T, Yamamoto Y, Inoue H, Nagasawa H, Kato T. Self-Organization of Oriented Calcium Carbon- ate/Polymer Composites: Effects of a Matrix Peptide Isolated from the Exoskeleton of a Crayfish. Angew Chem Int Ed. 2006;45: 2876–2879.

29. Becker C, Dick JTA, Cunningham EM, Schmitt C, Sigwart JD. The crustacean cuticle does not record chronological age: New evidence from the gastric mill ossicles. Arthropod Structure and Development. 2018;5: 498–512.

30. Joy DC, Pawley JB. High-resolution scanning electron microscopy. Ultramicrosc. 1992;47: 80–100.

31. Postek MT, Vladár AE. Does Your SEM Really Tell the Truth?–How Would You Know? Part 4: Charging and its Mitigation. Proc SPIE Int Soc Opt Eng. 2015;9636: 963605.

32. Harano K, Okada S, Furukawa S, Tanaka H, Nakamura E. Formation of a polycrystalline film of donor material on PEDOT:PSS buffer induced by crystal nucleation. J Polym Sci Part B: Polym Phys. 2014;52: 833–841.

33. Gorgoll RM, Harano K, Nakamura E. Nanoscale Control of Polymer Assembly on a Synthetic Catalyst–Bilayer System. J Am Chem Soc. 2016;138: 9675–9681.

34. Millero FJ, Feistel R, Wright DG, McDougall TJ. The composition of Standard Seawater and the definition of the Reference-Composition Salinity Scale. Deep Sea Res Part Oceanogr Res Pap. 2008;55: 50–72.

35. Obata H, Nishioka J, Kim T, Norisuye K, Takeda S, Wakuta Y, et al. Dissolved iron and zinc in Sagami Bay and the Izu–Ogasawara Trench. J Oceanogr. 2017;73: 333–344.

36. Cooksey C. Tyrian purple: the first four thousand years. Sci Prog. 2013;96: 171–186.

37. Sölter S, Dieckmann R, Blumenberg M, Francke W. Barettin, revisited? Tetrahedron Lett. 2002;43: 3385–3386.

38. Lovell FM. The structure of a bromine-rich marine antibiotic. J Am Chem Soc. 1966;88: 4510–4511.

39. Burkholder PR, Pfister RM, Leitz FH. Production of a pyrrole antibiotic by a marine bacterium. Appl Microbiol. 1966;14: 649–653.

40. Arai N, Sakamoto W, Maeda K. Analysis of Trace Elements in Otoliths of Red Sea Bream Pagrus major. Fish sci. 1995;61: 43–47.

41. Mucci A, Morse JW. The solubility of calcite in seawater solutions of various magnesium concentration, It = 0.697 m at 25 ° C and one atmosphere total pressure. Geochim Cosmochim Acta. 1984;48: 815–822.

42. Morse JW, Mucci A, Walter LM, Kaminsky MS. Magnesium interaction with the surface of calcite in seawater. Science. 1979;205: 904–905.

Supporting Information

S1 Fig. EDS maps of semi-thin sections of *H. gigas*. a) transverse section through a subsample of pereonite, b) transverse section of the cephalic region showing the gastric mill, c) magnified images of the gastric mill, the red rectangle area in (b). SEM images, total X-ray counts (cps), and elemental maps are shown, and the labels are denoted in the left top. 'Al' corresponds to the aluminum-like signal around 1.48 keV, that overlaps with bromine L-line, and thulium and ytterbium M-line. d–f) EDS sum spectra of the (d) pereonite, (e) cephalic region, and (f) gastric mill created from the EDS maps (a)–(c). SEM images in (a) were acquired at 1 kV, and other images and spectra were acquired at 10 kV.

S2 Fig. XRD spectrum of dried pereonite of *H. gigas*. Diffraction peak position in 2θ and corresponding crystal planes of calcite and α-chitin in parenthesis are shown.

S3 Fig. Quantitative point analysis of EPMA using semi-thin sections. a) SEM images of the gastric mill, b) SEM image of the pereonite, c) atomic ratio of the elements. K-line of the elements were used for the quantification. The large value of carbon and oxygen is ascribed to the conductive carbon coating and the underlying Kapton tape. Note that the amount of aluminum was <5% of that of bromine, and may contain some error because of the overlap of Al K-line with Br L-line, which is not completely separable under WDS condition (Fig. 2b). Nitrogen was also searched but not observed at any points (0.00), thus not shown.

S4 Fig. SEM images and EDS spectra of the gastric mill of *Hirondellea gigas*. a) low-magnification SEM image, b) magnified image of the red rectangle in (a), c) magnified image of the orange rectangle in b), d) magnified image of the blue rectangle in (b), e) magnified image of the yellow rectangle in (c), f) magnified image of the green rectangle in (d), g) EDS elemental mapping of the red rectangle in (a), h) EDS spectra of the yellow points in (a). Atom labels correspond to K-line except for Br*, which is L-line.

S5 Fig. SEM images and EDS spectra of the maxilla 2 of *Hirondellea gigas*. a) low-magnification SEM image, b) magnified image of the red rectangle in (a), c) magnified image of the orange rectangle in b), d) magnified image of the yellow rectangle in (c), e) EDS elemental mapping of the blue rectangle in (a), f) EDS spectra of the yellow points in (b). Atom labels correspond to K-line except for Br*, which is L-line.

S6 Fig. SEM images and EDS spectra of the maxilliped of *Hirondellea gigas*. a) low-magnification SEM image, b) magnified image of the red rectangle in (a), c) magnified image of the orange rectangle in b). Note that the solid between setae are unremoved sodium chloride, assigned based on EDS mapping in (e). d) EDS spectra of the yellow points in (b), e) EDS elemental mapping of the blue rectangle in (a). Atom labels correspond to K-line except for Br*, which is L-line.

S7 Fig. SEM images and EDS spectra of the mandible of *Hirondellea gigas*. a) low-magnification SEM image, b) magnified image of the red rectangle in (a), c) magnified image of the orange rectangle in b), d) magnified image of the yellow rectangle in (c), e) EDS maps of the blue rectangle in (a), f) EDS spectra of the yellow points in (b). Atom labels correspond to K-line except for Br*, which is L-line.

S8 Fig. SEM images and EDS spectra of the antenna of *Hirondellea gigas*. a) low-magnification SEM image, b) magnified image of the red rectangle in (a), c) magnified image of setae from the article in the yellow rectangle in (b), d) magnified image of the orange rectangle in (a), e) magnified image of short barbs in the green rectangle in (c), f) EDS elemental mapping of the blue rectangle in (a), g) EDS spectra of the yellow points in (a). Atom labels correspond to K-line except for Br*, which is L-line.

S9 Fig. SEM images and EDS spectra of the pleopod of *Hirondellea gigas*. a) low-magnification SEM montage image, b) magnified image of the red rectangle near the red triangle in (a) showing the two types of setae, c) magnified image of the yellow rectangle in b), d) EDS spectra of the yellow points in (a). Atom labels correspond to K-line except for Br*, which is L-line.

S10 Fig. SEM/EDS mapping of the pleopod of *Hirondellea gigas*. a) low-magnification SEM montage image, which is a part of Fig. S8, b) EDS elemental mapping of the blue rectangle in (a).

S11 Fig. SEM images and EDS spectra of the uropod of *Hirondellea gigas*. a) low-magnification SEM image, b) magnified image of the red rectangle, left middle in (a), c) magnified image of the orange rectangle in b), d) EDS spectra of the yellow points in (b), e) EDS elemental mapping of the blue rectangle in (a). Atom labels correspond to K-line except for Br*, which is L-line.

S12 Fig. SEM images and EDS spectra of the pereonite of *Hirondellea gigas*. a) low-magnification SEM image, b) magnified image of the red rectangle, left middle in (a), c) magnified image of the orange rectangle in (b) showing the hexagonal elements, d) magnified image of (b) showing the bead-like structure, e) EDS sum spectra of (e). The surface was elementally homogeneous and the EDS maps are not shown. Atom labels correspond to K-line except for Br*, which is L-line.

S1 Table. Cross correlation of EPMA elemental maps of semi-thin section of the pereonite in Fig. 2. Top 10 values are shown in bold for visibility.

S2 Table. Cross correlation of EPMA elemental maps of semi-thin section of the gastric mill in Fig. 3. Top 10 values are shown in bold for visibility.

S3 Table. Elemental ratios of EDS point/area analyses in Fig. S4–13. GM, gastric mill; Mxp, maxillipet; Md, mandible; Mx2, maxilla 2; An, antenna; Pl, pleopod; Ur, uropod; Cx, coxal plate; Pe, pereonite. Values are reported by the atomic ratio ± errors in per cent. Note that the spectra were acquired under non-optimal condition, i.e. curved surface, and may contain large errors in the relative values. Area analysis was performed on Cx and Pe.

Fig 1

Click here to access/download;Figure;Fig1.tif

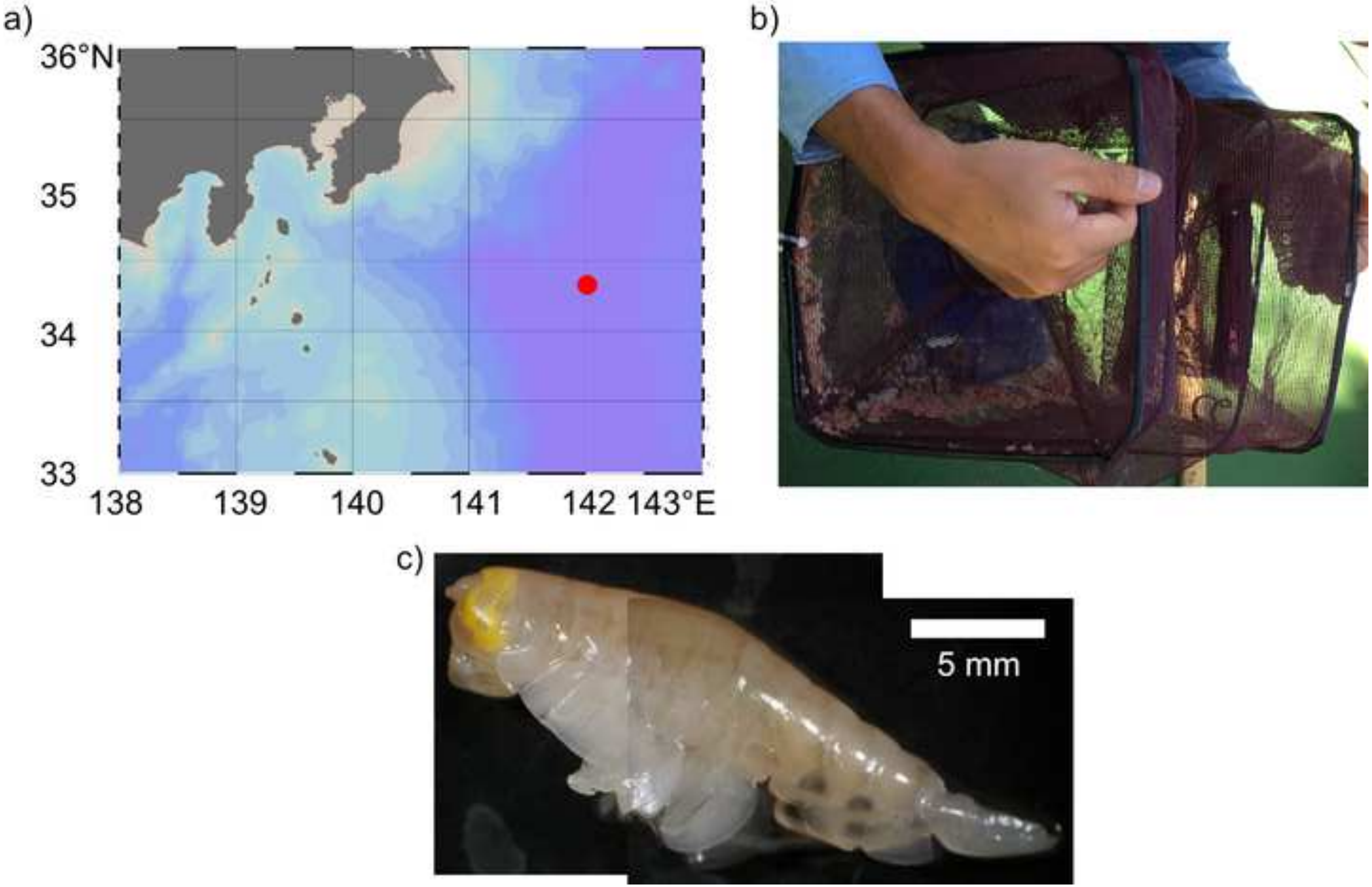

Fig 2

Click here to access/download;Figure;Fig2.tif

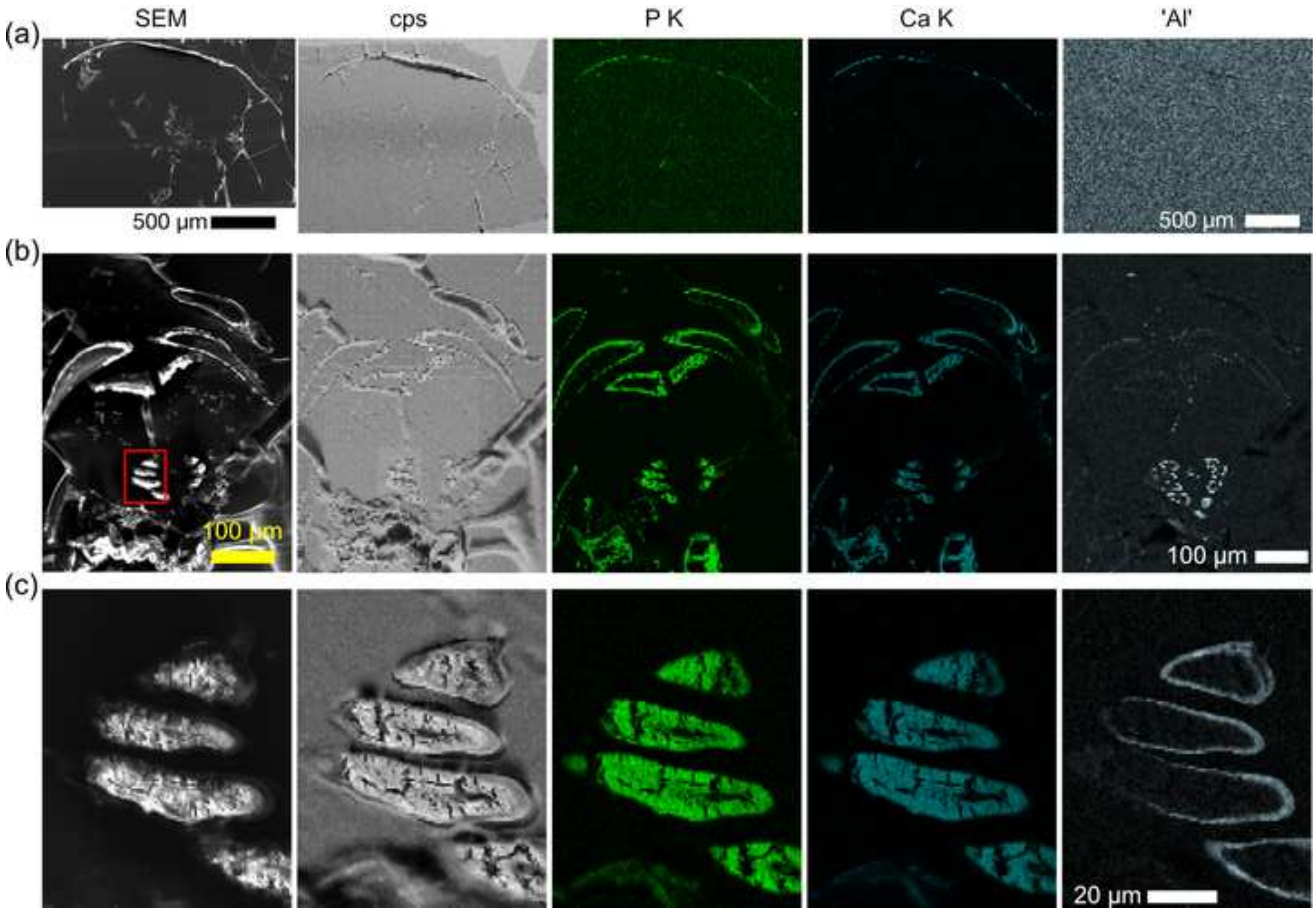

Fig 3

Click here to access/download;Figure;Fig3.tif

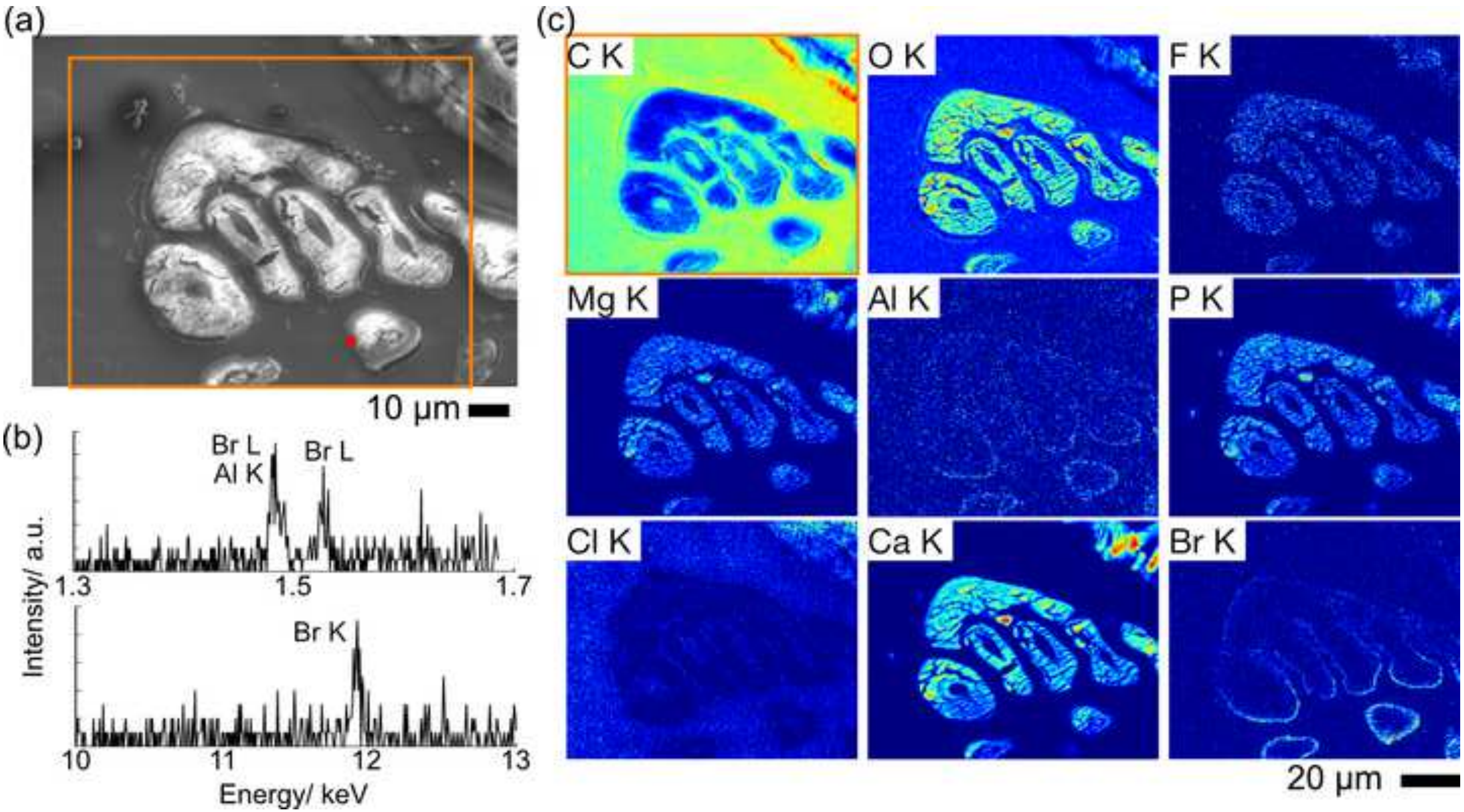

Fig 4

Click here to access/download;Figure;Fig4.tif

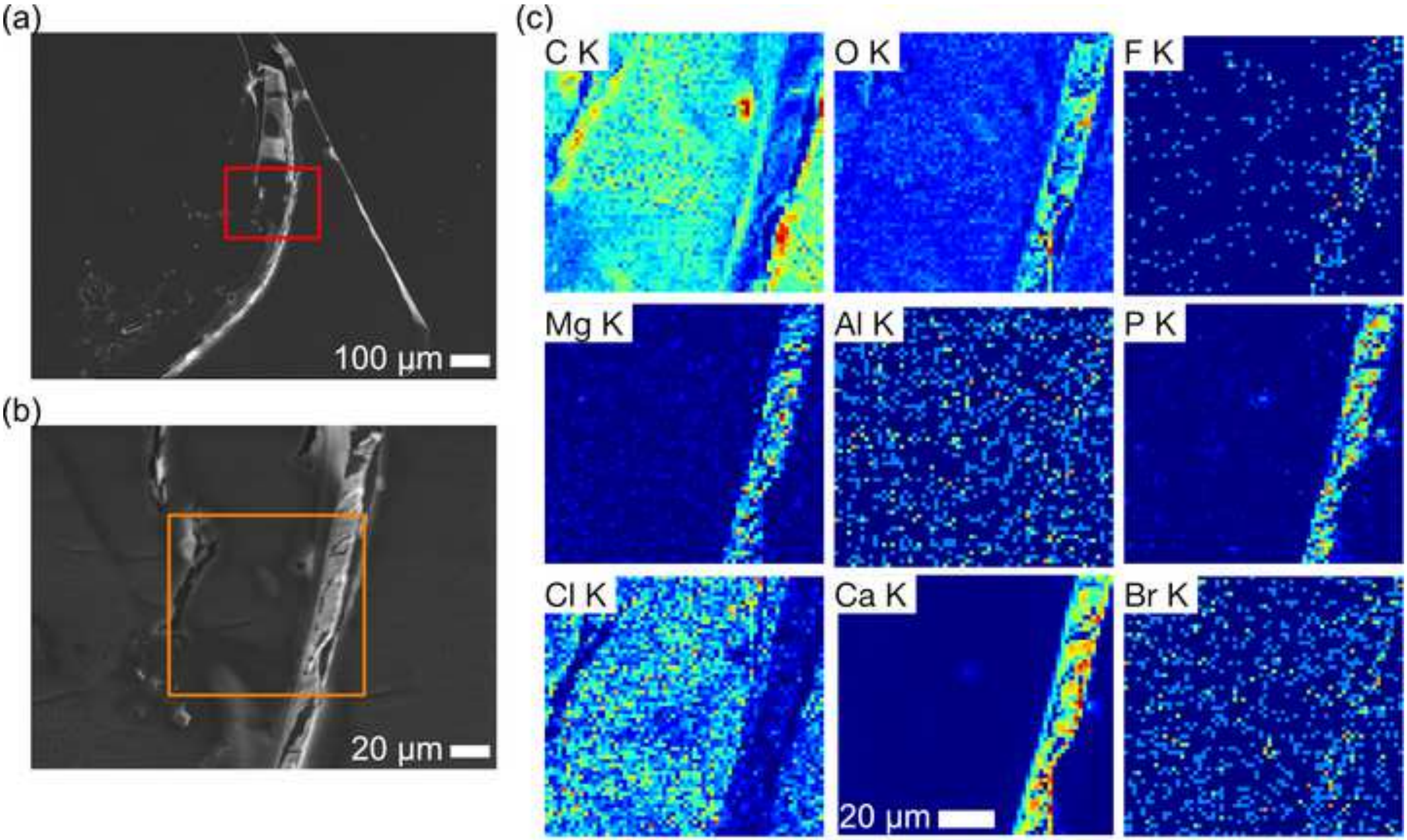

Fig 5
Click here to access/download;Figure;Fig5.tif

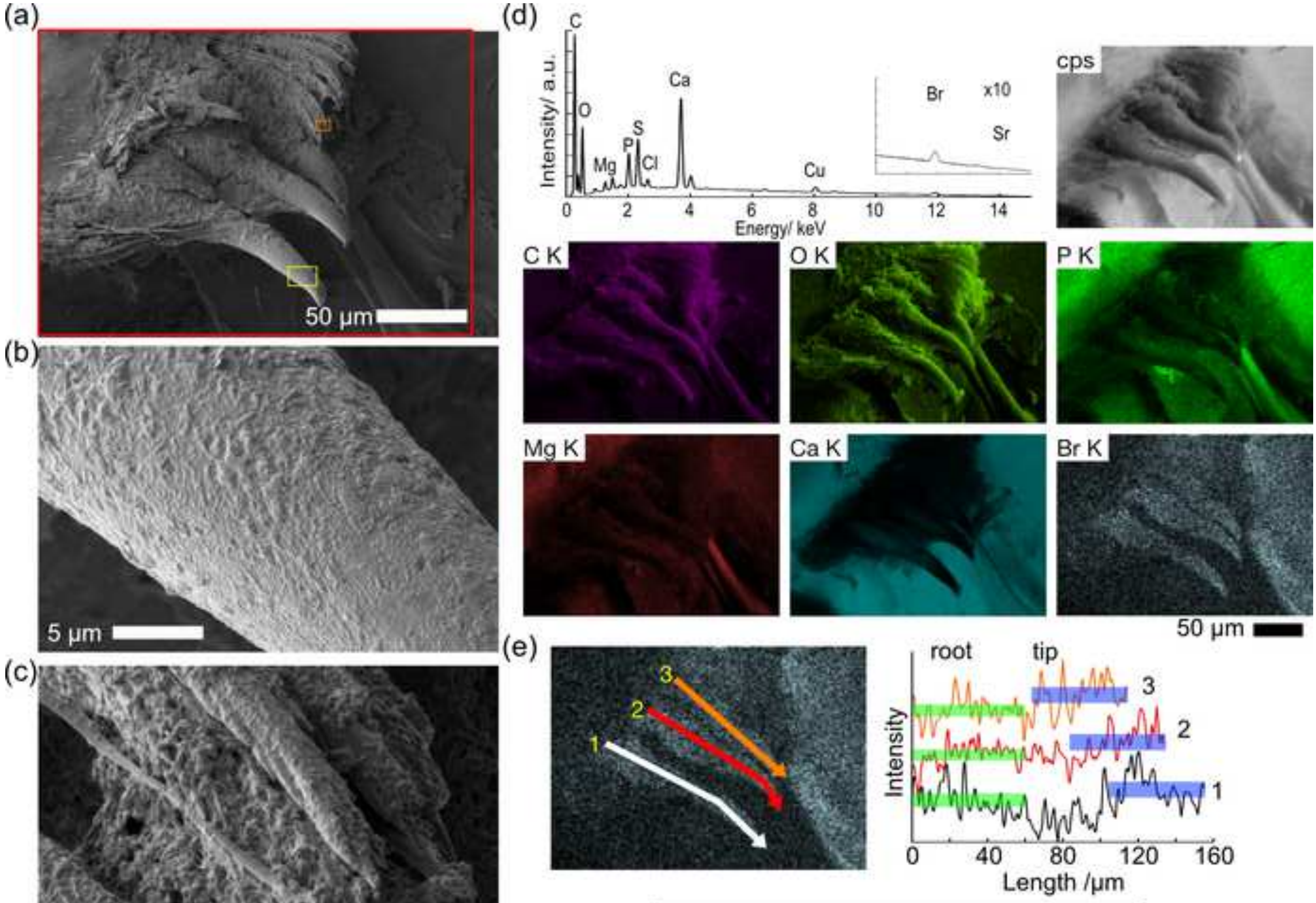

	1	2	3
root 60 μm	12.9±2.6	12.5±2.1	12.0±2.2
tip 50 μm	14.9±2.9	14.8±3.1	14.8±3.0

Fig 6

Click here to access/download;Figure;Fig6.tif

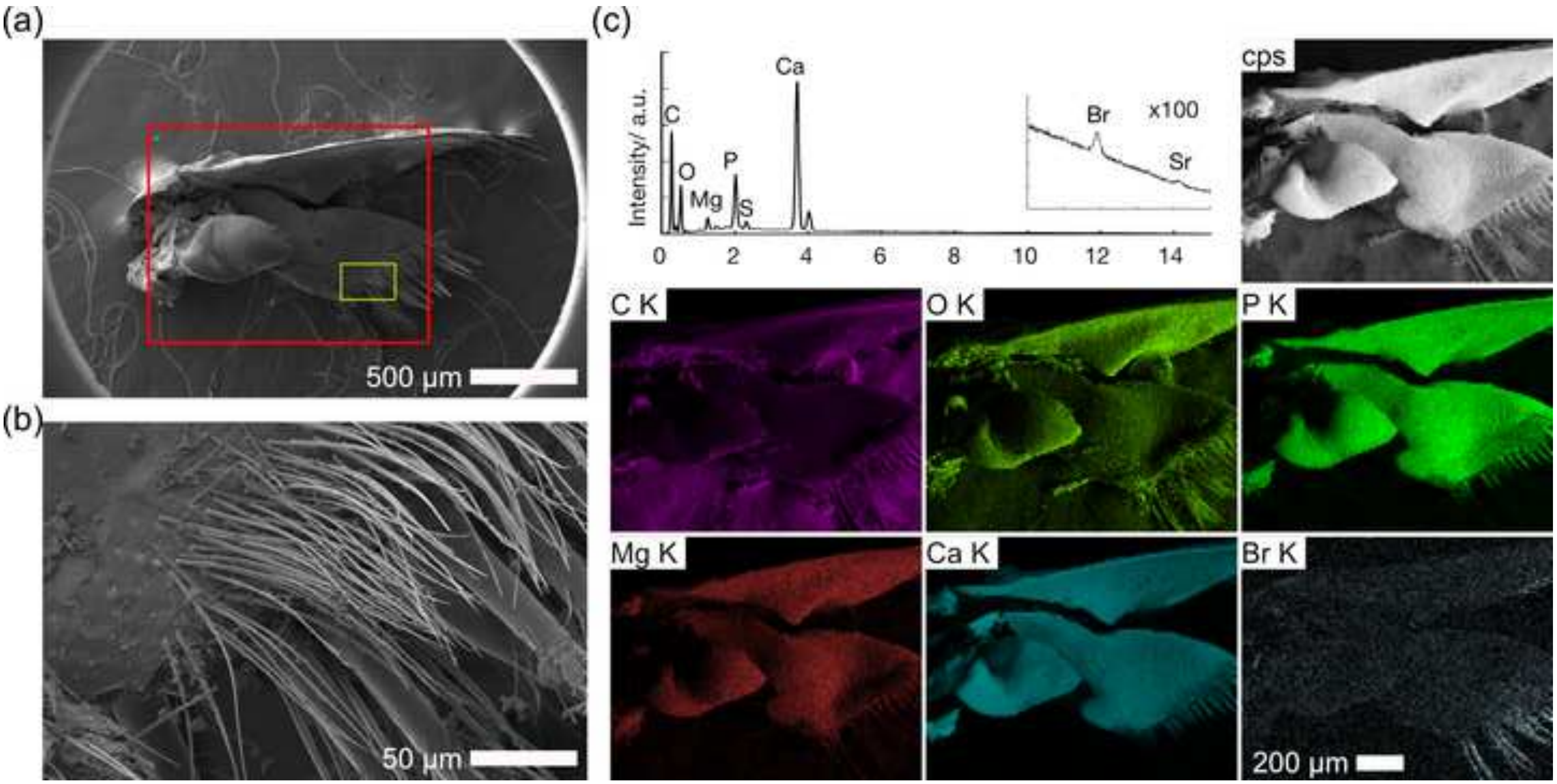

Fig 7

Click here to access/download;Figure;Fig7.tif

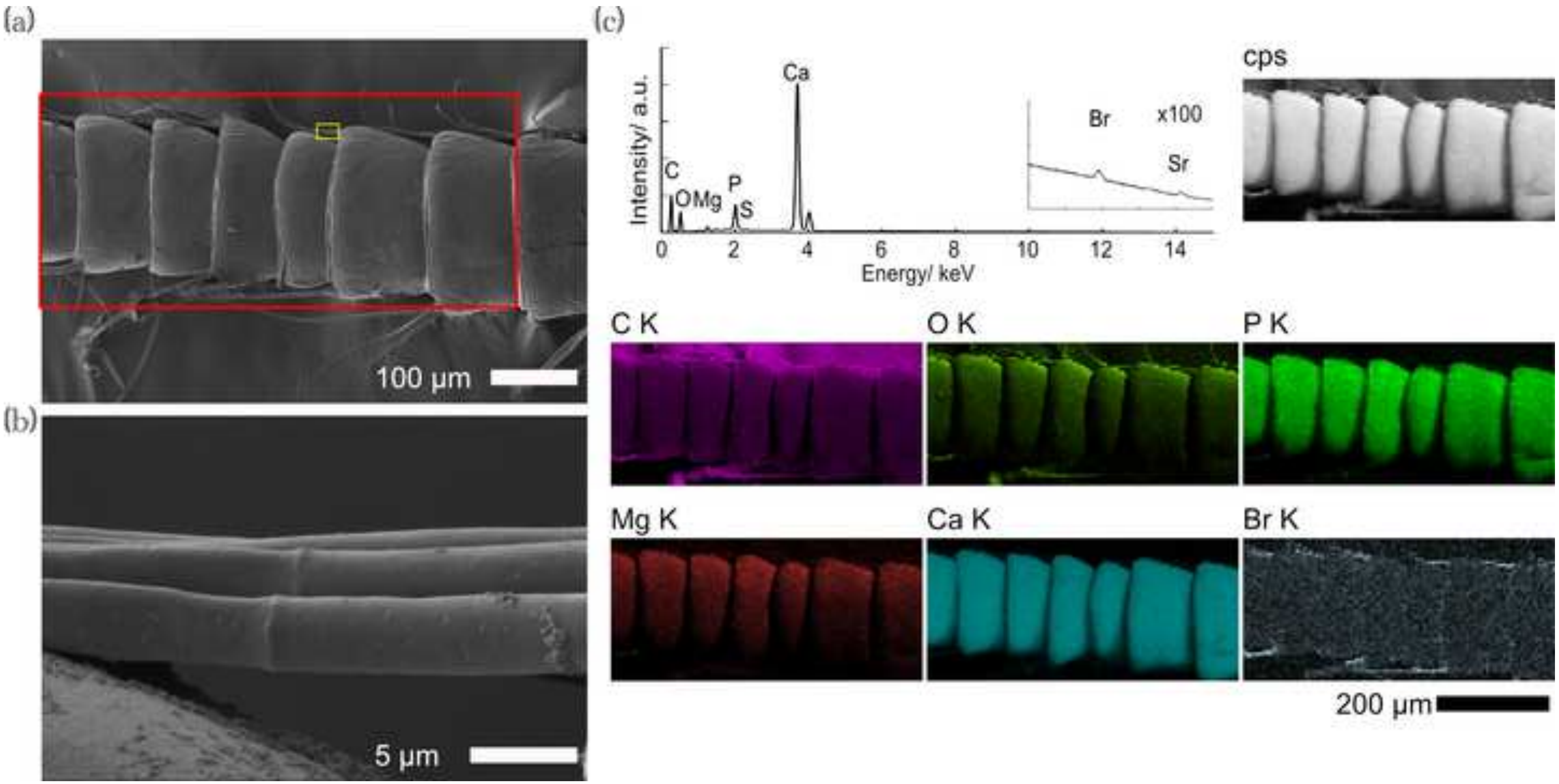

Fig 8

Click here to access/download;Figure;Fig8.tif

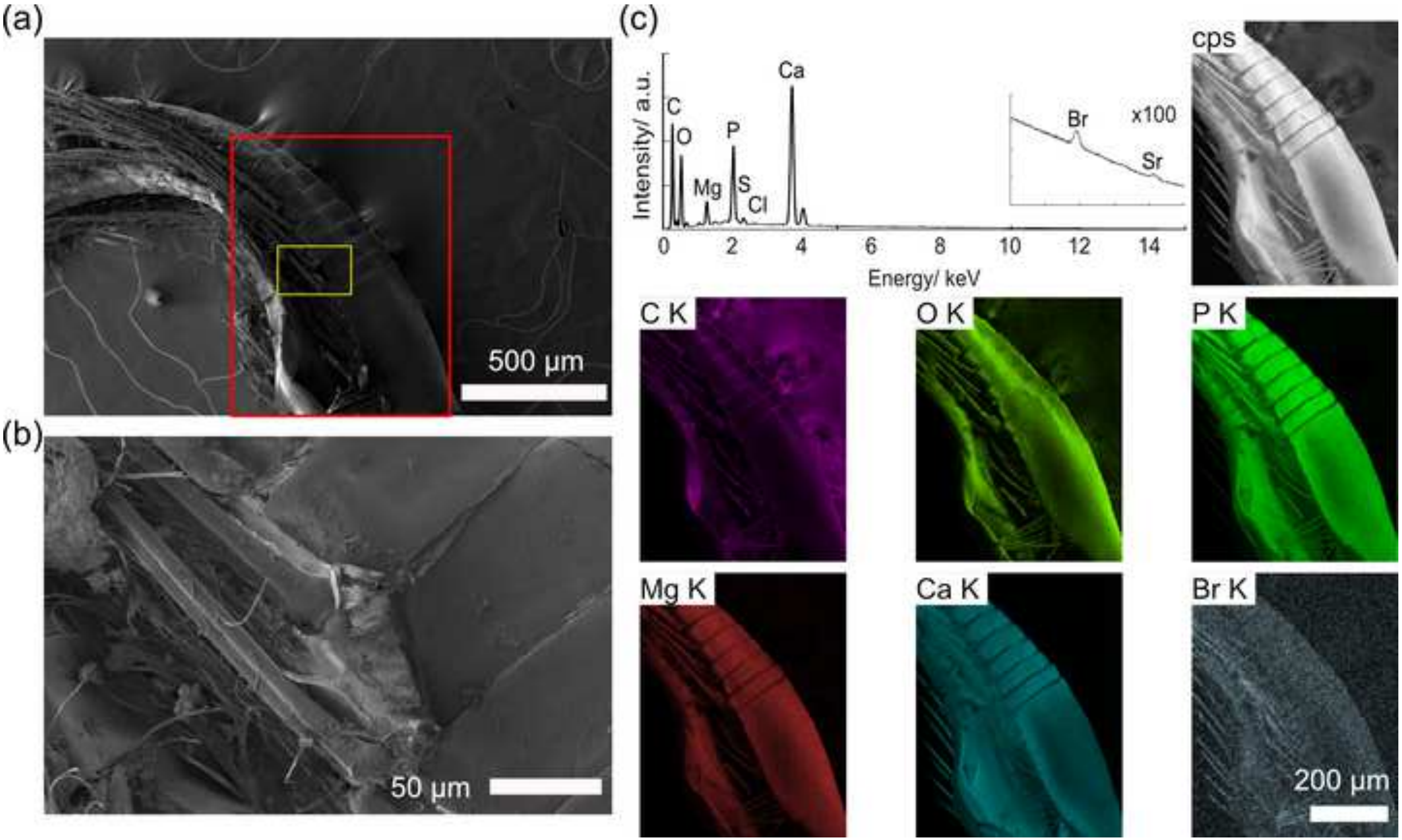

Fig 9

Click here to access/download;Figure;Fig9.tif

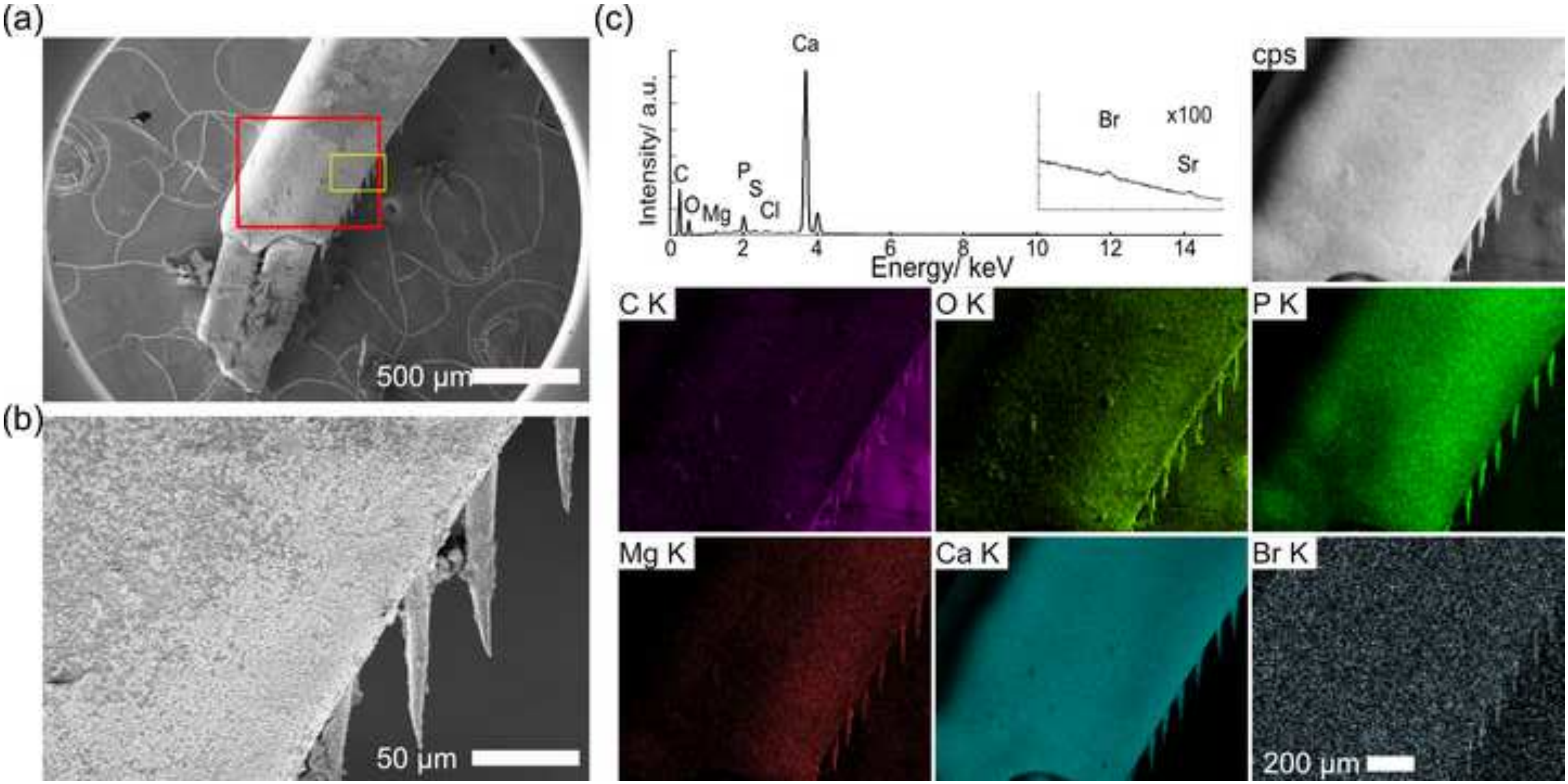

Fig 10

Click here to access/download;Figure;Fig10.tif

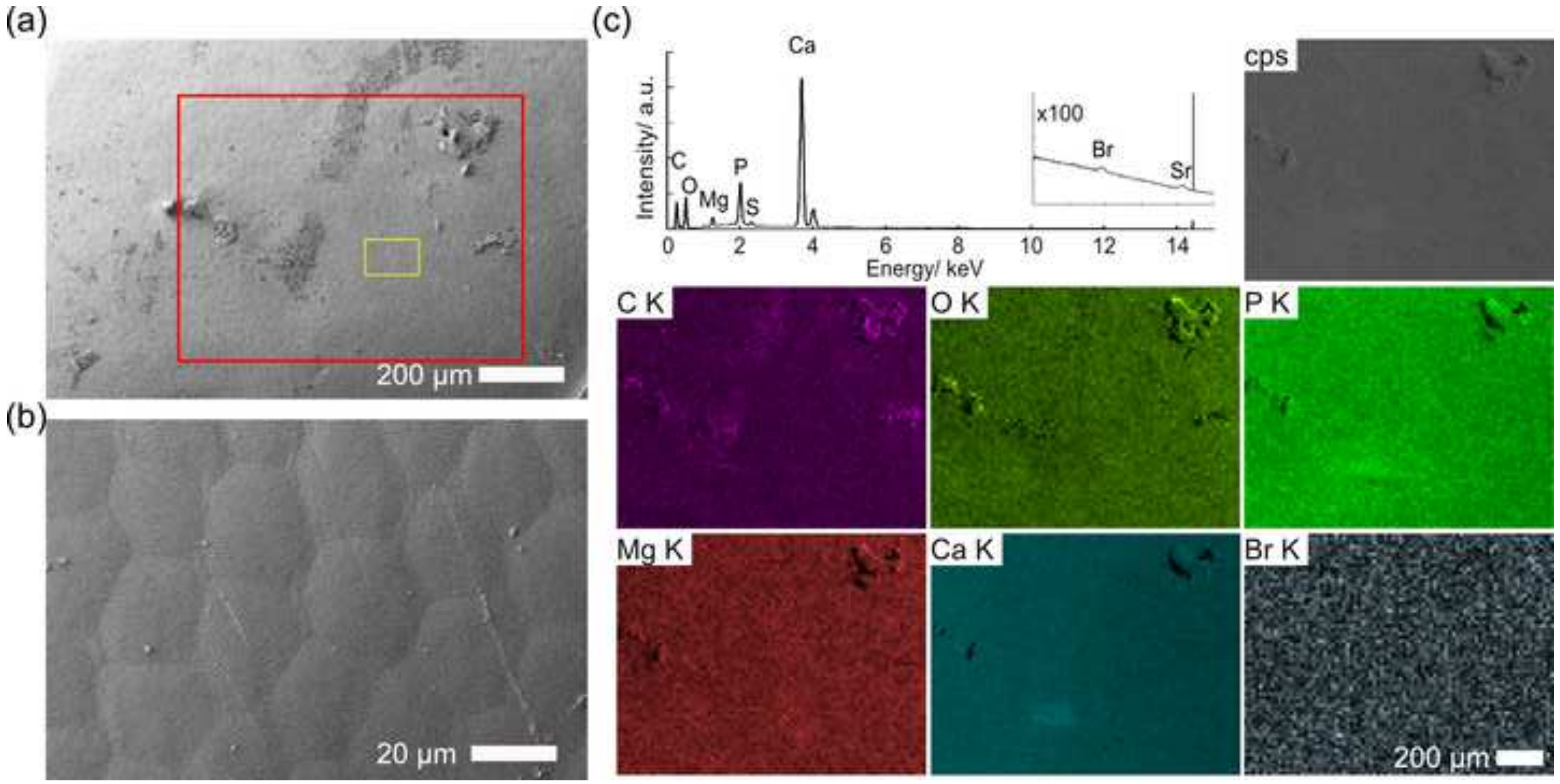

S1 Fig

Click here to access/download
Supporting Information
S1_Fig.tif

S2 Fig

Click here to access/download
Supporting Information
S2_Fig.tif

S3 Fig

Click here to access/download

Supporting Information

S3_Fig.tif

S4 Fig

Click here to access/download
Supporting Information
S4_Fig.tif

Click here to access/download
Supporting Information
S5_Fig.tif

S6 Fig

Click here to access/download
Supporting Information
S6_Fig.tif

S7 Fig

Click here to access/download
Supporting Information
S7_Fig.tif

S8 Fig

Click here to access/download

Supporting Information

S8_Fig.tif

S9 Fig

Click here to access/download
Supporting Information
S9_Fig.tif

S10 Fig

Click here to access/download
Supporting Information
S10_Fig.tif

S11 Fig

Click here to access/download
Supporting Information
S11_Fig.tif

S12 Fig

Click here to access/download
Supporting Information
S12_Fig.tif

S1 Table

Click here to access/download
Supporting Information
S1_Table.pdf

S2 Table

Click here to access/download
Supporting Information
S2_Table.pdf

S3 Table

Click here to access/download
Supporting Information
S3_Table.pdf

Unusual bromine enrichment in the gastric mill and setae of the hadal amphipod *Hirondellea gigas*

Satoshi Okada[1*], Chong Chen[1], Hiromi Kayama Watanabe[1], Noriyuki Isobe[2], Ken Takai[1]

[1] Institute for Extra-cutting-edge Science and Technology Avant-garde Research (X-STAR), Japan Agency for Marine-Earth Science and Technology (JAMSTEC), 2-15 Natsushima-cho, Yokosuka, Kanagawa 237-0061, Japan

[2] Biogeochemistry Research Center, Research Institute for Marine Resources Utilization (MRU), Japan Agency for Marine-Earth Science and Technology (JAMSTEC), 2-15 Natsushima-cho, Yokosuka, Kanagawa 237-0061, Japan

* Corresponding author

E-mail: okadasa@jamstec.go.jp

Style Definition: Normal: Left, Right: 0", Line spacing: single, Tab stops: Not at -1.08"

Style Definition: Heading 1: Font: 18 pt, Left, Right: 0", Line spacing: single, Tab stops: Not at -1.08"

Style Definition: Heading 2: Font: 16 pt, Left, Right: 0", Line spacing: single, Tab stops: Not at -1.08"

Formatted: Numbering: Continuous

Formatted: Not Superscript/ Subscript

Formatted: Not Superscript/ Subscript

Formatted: Normal, Position: Horizontal: Left, Relative to: Column, Vertical: In line, Relative to:

Abstract ~~(abstract 150-200 words)~~

The hadal amphipod *Hirondellea gigas* is an emblematic animal of the Pacific trenches, and has a number of special adaptations to thrive in this 'extreme' environment, which includes including the deepest part of the Earth's ocean. One such adaptation that has been suggested is the presence of an 'aluminum gel shield' on the surface of its body in order to prevent the dissolution of calcitic exoskeleton below the carbonate compensation depth. However, this has not been investigated under experimental conditions that sufficiently prevent~~s~~ aluminum artefacts, and the possibility of other elements with similar characteristic X-ray energy as aluminum (such as bromine) has not been considered. Here, we show with new electron microscopy data ~~carried~~ gathered under optimized conditions to minimize aluminum artefacts that *H. gigas* actually does not have an aluminum shield – instead many parts of its body are enriched in bromine, particularly gastric ossicles and setae. Results from elemental analyses pointed to the use of calcite partially substituted with magnesium by *H. gigas* in its exoskeleton, in order to suppress dissolution. Our results exemplify the necessity of careful sample preparation and analysis of the signals in energy-dispersive X-ray spectroscopic ~~EDS~~ analysis, and the importance ~~to~~ analy~~ze~~ses at different electron energies ~~in microscopic 'elemental anatomy'~~.

~~Main text~~Introduction

Many animal groups have evolved hard parts for skeletal support (e.g. vertebrate bones), protection (e.g. molluscan shells), and feeding (e.g. mouth parts and teeth), among other functions – and often for multiple purposes. These hard parts are composed of either inorganic minerals, such as calcite, aragonite, and hydroxyapatite, or organic polymers, such as chitin, or a mixture of both. Inorganic biomineralized parts exposed to the outside world are often coated with organic matrices, such as molluscan shells covered by a layer of periostracum made out of a combination of chitin and proteins.[1]~~1~~ In some cases such as the marine polychaete worm *Nereis virens*, heavy transition metals and halogens are utilized to coat the mineralized tissues.[2,3]~~2,3~~ These external layers often play roles in the secretion of the biominerals, and they can also reinforce the structure and prevent the deterioration of the underlying mineral layer. For example, bivalves living deeper than the carbonate compensation depth (CCD; typically ~4500 m), where calcium carbonate dissolves quickly, use their periostracum to buffer against the natural shell dissolution.[4]~~4~~

Crustaceans are among the most successful invertebrates in marine environments and use hard parts for both protection in the form of calcified exoskeletons and feeding in the form of gastric mills. Their exoskeletons are mainly composed of chitinous

Formatted: Font color: Auto

Formatted: Font: Helvetica, Font color: Black

Formatted: Font: Helvetica, Font color: Black

Formatted: Font: Helvetica, Font color: Black

Formatted: Normal, Position: Horizontal: Left, Relative to: Column, Vertical: In line, Relative to:

biopolymers hardened through sclerotization, the cross-linking of protein components.[5,6] To achieve further mechanical strength and/or adaptive functionality, the exoskeletons are often additionally impregnated with other materials. The most widespread example is amorphous calcium carbonate and calcium phosphate.[5–7] Though most crustaceans with calcified exoskeleton predominantly use calcium carbonate, some such as barnacles in the genus *Ibla* chiefly utilize calcium phosphate.[8,9] Phosphate being more common in mandibles.[10] Calcium phosphate is often used to strengthen the mandible, such as in the crayfish which forms fluoroapatite as a crown on its mandible.[11,12] Some crustaceans such as woodlices, use crystalline magnesium calcite where a part of calcium is substituted with magnesium.[13] Furthermore, a number of crustaceans exhibit further coatings outside the exoskeleton. For examples barnacles where the hardened plates are overlaid with cuticular and epicuticular layers,[8] deep-sea ostracods which cover the mineralized layer with epidermis to prevent mineral dissolution even below the CCD,[14,15] and crabs such as *Pachygrapsus crassipes* possesses bromine-enriched coating on their claw tips that increases the fracture-resistance.[16,17] ~~However, coatings of organic matrices in crustacean hard parts are not known with the sole exception of barnacle cuticular and epicuticular layers covering the plates.[5]~~

The hadal amphipod *Hirondellea gigas* is one of the most numerically dominant crustacean and ~~megafauna~~ macrofauna at the deepest ~~parts~~ areas of the ocean, including the Challenger Deep in the Mariana Trench (10,994 m).[18]~~[6]~~ As special adaptations to ~~the~~ this extreme hadal environment, this species possesses unique cellulase and is alleged to digest polysaccharides from sunken plant matter using a unique cellulase~~,~~.[18,19]~~[6,7]~~ ~~and p~~Perhaps most strikingly, it is said to secrete aluminum to the outside its exoskeleton to prevent the dissolution of calcium-based minerals, dubbed the 'aluminum shield'.[20]~~[8]~~ Based on the~~The aluminum shield was expected to be in a form of gel, supported by~~ combination of energy-dispersive X-ray analysis (EDS) under scanning electron microscopy (SEM), and aluminum extraction experiments from the sediment of Challenger Deep, ~~.~~ Kobayashi et al.[20] expected that the 'aluminum shield' of *H. gigas* would be in gel form. However, their results ~~was~~ were inconclusive and ambiguous due to the localization pattern of aluminum, where the signal intensity of aluminum was especially high only at the edge of the samples close to the aluminum stubs most prone to artefacts. Furthermore, samples were not covered with conductive coatings were not performed for imaging, despite observations being undertaken in ~~an~~ the aluminum-rich environment that is the SEM chamber. In addition, EDS spectra under a scanning transmission electron microscope (STEM) of the exoskeleton did not provide enough information to support the existence of aluminum and its detailed nanostructure.[20]

Formatted: Font color: Black
Formatted: Font color: Black
Formatted: Font color: Black
Formatted: Font color: Black
Formatted: Font color: Black
Formatted: Font color: Black
Formatted: Font color: Black
Formatted: Font color: Black
Formatted: Font color: Black
Formatted: Font color: Black
Formatted: Font color: Black
Formatted: Font: Helvetica, Font color: Black
Formatted: Font color: Black
Formatted: Font color: Black
Formatted: Normal, Position: Horizontal: Left, Relative to: Column, Vertical: In line, Relative to:

In order to confirm the presence of the 'aluminum shield' of *H. gigas* and to further substantiate its distribution and potential function, we designed a more careful experimental design to exclude aluminum artefacts.

There are two possibilities where aluminum artefacts may be observed: 1) backscattered electrons (BSE) from the observed specimen hitting the chamber made of aluminum alloy to generate secondary electrons (called SE3) as well as aluminum X-ray,[21][9]) overlapping of other elements that has have similar characteristic X-ray energy signatures to that of aluminum (Al K-line, 1.486 keV) and which are inseparable using conventional EDS whose energy resolution being ~130 eV. These elements include bromine (Br L-line, 1.480 keV), thulium (Tm M-line, 1.462 keV), and ytterbium (Yb M-line, 1.521 keV),[22][10] the latter two being is unused in animals.[23,24][11,12] Here, we report our findings from detailed electron microscopy observations of *H. gigas*, revealingOur detailed analysis revealed the absence of aluminum on the exoskeleton and instead the presence accumulation of bromine on hard tissues as the sclerotized spines of its gastric mill components, a rare example of in *H. gigas*. We serendipitously also discovered a unusual local enrichment of bromine on the surface of the sclerotized spines of its gastric mill components, including clear accumulation on the spines. Although bromine is a common element in seawater,[13] it is rarely preferentially secreted bromineed in the hard parts of animals, except in some cases where bromine with transition metals are utilized to harden the hard parts.[2,3,14] Here, we report our findings from electron microscopic observations of *H. gigas*, with a focus on its bromine accumulation.

Formatted: Font color: Black

Formatted: Font color: Black

Formatted: Font: Helvetica, Font color: Black

MethodsMaterials & Methods

Materials

All the reagents were purchased either from Nacalai tesque Inc., Fuji Film Wako Pure Chemical Co., or Kanto Chemical Co., Inc. and used as received. Deionized water was supplied from Merck Milli-Q Integral 5.

Amphipod samples

Hirondellea gigas individuals were collected using a bait trap attached to a Free Fall Camera System at Izu-Ogasawara Trench near the Boso Triple Junction (34°20.4296′N 142°0.6006′E, Fig 1a), depth 9218 m on September 4, 2019, on R/V Yokosuka cruise YK19-11. Over 200 individuals (Fig 1b) were collected, of which three individualsThe samples were used for this study. The amphipods were immediately immersed in slush nitrogen (ca. –210 °C) for more than a minute to rapidly freeze the exoskeleton and stored at –80 °C during the cruise, and then transferred to liquid nitrogen storage (actual storage temperature <–160 °C) after cruise.

Formatted: Normal, Position: Horizontal: Left, Relative to: Column, Vertical: In line, Relative to:

Fig 1. Sampling of *Hirondellea gigas*. a) Sampling location in a red dot. b) *H. gigas* collected in the bait trap. More than 200 individuals are collected. c) A micrographic montage of *H. gigas* under stereomicroscope.

Electron microscope instruments

SEM and scanning transmission electron microscopic (STEM) imaging were performed on a Helios G4 UX (Thermo Fisher Scientific), an Octane Elite Super energy dispersive X-ray analysis (EDS) detector (Octane Elite Super, AMETEK) and a cryogenic stage with preparation chamber (PP3010T, Quorum). Transmission electron microscopy (TEM) was performed on a Tecnai G2 20 (Thermo Fisher Scientific, operated at 200 kV) equipped with a bottom-mounted 2k ~~×x~~ 2k Eagle Charge-Coupled Device camera (Eagle, Thermo Fisher Scientific) and RTEM-S 61700NE an EDS detector (RTEM-S 61700NE, AMETEK). Electron probe microanalysis (EPMA) was performed on a JXA-8500 (JEOL Ltd.) equipped with five wave~~-~~length dispersive X-ray detectors (WDS, XM-86030 and XM-86010, JEOL Ltd.).

Sample preparation and semi-thin section imaging

~~The f~~Frozen amphipods ~~was~~ were dehydrated in acetone at –80 °C for three days~~,~~ and gradually warmed to room temperature. The dehydrated amphipod was ~~replaced with~~immersed into QY-1 (Ohkenshoji Inc.), QY-1/epoxy mixture, then with an epoxy resin (TAAB), trimmed into pieces using laser blades, and embedded in the epoxy resin following the Luft's method. [25]~~[15]~~ Transverse sections of the head part were~~was~~ taken from ~~the~~ anterior to~~wards the~~ posterior into 1 μm-thick semi-thin sections using an ultramicrotome (Ultracut S or EM UC7, Leica) with a diamond knife (45°, Diatome).~~, the s~~Sections were collected on a glass slide covered with Kapton tape (Nitto Denko Co., P-224, 64 μm thickness) using a nickel loop. The slide was coated with carbon for conductivity using CADE (Meiwaforsis Inc.). Semi-thin sections of pereonite were prepared in the same manner.

Semi-thin sections were subjected to SEM/EDS analysis operated at 1 kV for morphological imaging~~,~~ and at 10 kV for elemental analysis and mapping. Chlorine was observed uniformly in the epoxy resin as its contaminant, and carbon coating ~~were~~ was performed for conductivity on ~~carbon~~the polyimide-based substrate~~, and~~. However, the localization of these two elements ~~were~~ was not discussed in detail.

Elemental analysis at high energy resolution was performed on an EPMA operated at 20 kV and beam current of 12 nA. The elemental colocalization in the EPMA

Formatted: Font: Bold

Formatted: Font: Helvetica, Font color: Black

Formatted: Normal, Position: Horizontal: Left, Relative to: Column, Vertical: In line, Relative to:

map was quantified using cross-~~-~~correlation image analysis.[26]~~[16]~~ The cross-correlation factor γ between two elemental maps *a* and *b* was calculated as

$$\gamma(a,b) = \frac{\sum_{ij}\{[I_a(r_{ij}) - \bar{I}_a] \cdot [I_b(r_{ij}) - \bar{I}_b]\}}{\sqrt{\sum_{ij}[I_a(r_{ij}) - \bar{I}_a]^2} \cdot \sqrt{\sum_{ij}[I_b(r_{ij}) - \bar{I}_b]^2}} \quad (1)$$

where $I_x(r_{ij})$ denotes the X-ray intensity of map x at the pixel position (~~i~~I, j), and $\bar{I}_x$ is the averaged intensity of map x over the image. The cross-correlation calculation and EPMA elemental mapping were implemented in Python 3.9.1.

Microscopic observation and image analysis of a dissected amphipod

Another frozen individual of *H. gigas* (total length 26.0 mm, telson length 5.3 mm) was defrosted and dissected for three-dimensional structural investigation under a stereomicroscope (SZX7, Olympus) and photos were taken using an Olympus E-M5 Mark III DSLR camera (Fig 1c). The amphipod was dissected into different parts, with observations focusing on: mandible, maxilla, maxilliped, antennae, gastric mill (pterocardiac ossicles in the cardiac stomach), pereonites, pleopod, uropod, and coxal plates. Dissected samples were placed within Eppendorf tubes (0.2 mL), dehydrated by an increasing series of ethanol (50, 60, 70, 70, 70, 80, 90, 99, 100, 100, 100%, with more than 10 minutes ~~for~~ at each step), then substituted with *tert*-butanol, frozen at −20 °C, and dried under vacuum (<1 ×~~x~~ 10^{-1} Pa) to obtain dried specimens, which were then mounted on a Kapton tape on a glass slide using a double-sided carbon adhesive tape. The mounted samples were coated with carbon for conductivity, and analyzed in SEM/EDS operated at 1 kV for morphological imaging and 10 or 20 kV for EDS analyses.

~~Precautions to avoiding artificial aluminum signals~~

~~To observe the potential aluminum layer in the exoskeleton of *H. gigas*, several precautions were taken to eliminate potential artificial X-ray signals from aluminum sources, which is a very common element and contaminant in electron microscopy.[11] A glass slide covered with carbon-coated Kapton tape was selected as an aluminum-free substrate for SEM imaging. The use of Kapton/carbon reduces emission of backscattered electron (BSE) from the substrate, which may generate secondary electrons (SE3)~~

Formatted: Font color: Black

Formatted: Heading 2

Formatted: Normal, Position: Horizontal: Left, Relative to: Column, Vertical: In line, Relative to:

and X-ray by hitting the SEM chamber made of aluminum alloy.[9] The amphipod tissue was mounted as carbon-coated flat semi-thin sections (unlike the bulk specimen in [[8]]) due to three benefits. Firstly, the spatial resolution of semi-thin section is higher than that of using a bulk specimen where the irradiated electron beam would scatter within the sample and generates X-ray from the interior and surrounding positions of the irradiated position, resulting in decreased spatial resolution and make it undecisive whether the signal actually originated directly from the observed surface – the use of semi-thin sections can avoid this issue. Secondly, the charge-up of the thin section is minimized, as primary electrons injected to the thin section are not retained in the sample but instead penetrate through to the underlying layer, where they are neutralized by conductive coatings.[17] In the case of bulk samples with insulative interior, as is in such cases as observation of biological materials, accumulated charges within the sample bends the electron trajectory, resulting in decreased spatial resolution. Finally, using semi-thin sections ensure the electrons and X-rays are emitted homogeneously from a flat surface, which is desirable for quantitative analysis. Surface roughness causes abnormal contrast such as edge effect to brighten edges, and shading of X-rays to decrease signal intensities of low-energy X-ray.[18] Without these precautions, total X-ray counts from each position does not necessarily reflect the sample's true elemental composition. We used the counts per pixel (cps) images, showing total X-ray counts from each pixel, as the criteria to distinguish whether the signal originated from the sample or from the background (bremsstrahlung).

Formatted: Normal, Position: Horizontal: Left, Relative to: Column, Vertical: In line, Relative to:

~~The condition to observe SEM/EDS maps were set either to 10 kV or to 30 kV. The former condition was used to enhance spatial resolution and signal intensities of light elements, and the latter to observe high-energy characteristic X-rays from possible heavy elements.~~

X-ray measurement

Dried pereonites were cut into flat pieces and mounted on an X-ray diffractometer (X′Pert PRO, ~~(~~PANalytical) with ~~with~~ a Cu radiation source (Kα = 1.5418 ~~A~~Å) at 45 kV and 40 mA. The diffractogram from 10° to 60°was acquired at 0.0167° steps at the scan speed of 0.07°/s. The 16 spectra were integrated, and the mineral assignment was performed on the software (HighScore 3.0.5, ~~(~~PANalytical).

Results

Elemental distribution and composition of *H. gigas*

To observe the potential aluminum layer in the exoskeleton ~~Following the precautional methods for the EDS measurement to avoid artificial aluminum signals, thin sections~~ of *Hirondellea gigas*, the elemental composition was investigated by ~~were subjected to~~ SEM/EDS of thin sections to avoid artificial aluminum signals caused by the substrate and non-flat surfaces ~~analyses~~ (~~Fig 1~~Figs 2, ~~, Fig S~~S1 Fig~~1~~). EDS maps of the pereonite and telson showed no evidence of aluminum, while strong signals of carbon, oxygen, phosphorous, and calcium as well as a weak signal of sulfur were observed in the area where bright contrast was observed in the SEM images (~~Fig 1~~Figs 2a, S1a Fig). This result agrees with the previous observation[20]~~[8]~~ that the exoskeleton is composed of calcium carbonate and proteinaceous organic materials. Other elements, such as sodium, aluminum, and silicon were within the background levels of the continuous ~~bremsstrahlung~~ X-ray (bremsstrahlung), ~~.~~ while chlorine was from the epoxy resin. The EDS maps showed uniform existence of these elements, indicating the compositional homogeneity of the exoskeleton. Soft parts within the exoskeleton were composed of organic materials, and the signal of chlorine corresponds to the epoxy resin. The intensities of the aluminum-like signal (as aluminum overlaps with that of bromine, thulium, and ytterbium) was similar to that of the background signals, indicating that this signal was below the detection limit of the imaging condition used here.

Formatted: Font: Palatino Linotype

Formatted: Font: Not Italic

Formatted: Font: Helvetica, Font color: Black

Formatted: Normal, Position: Horizontal: Left, Relative to: Column, Vertical: In line, Relative to:

Fig 2. EDS maps of semi-thin sections of *H. gigas*. a) transverse section through a subsample of pereonite, b) transverse section of the cephalic region showing the gastric mill, c) magnified images of the gastric mill, the red rectangle area in (b). Images from the left are SEM images at (a,b) 1 kV and (c) 10 kV, total X-ray counts (grayscale), phosphorous (green), calcium (blue), and aluminum-like signals around 1.48 keV (silver). EDS spectra were acquired at 10 kV, thus the K-line of bromine was not observed.

To confirm that the mineral components of the pereonites of *H. gigas* is are the same as previously observed (i.e. mainly calcitic),[20][8] XRD measurements was were performed using the dissected dried pereonite. Nine among of ten peaks were assigned to that of calcite (Fig. S2S2 Fig). The residual peaks at 19.7° and 26.7° were explained by α-chitin, which is ubiquitous in crustacean exoskeletons.[27,28][19,20] This result combined with EDS analysis confirmed that the exoskeleton of *H. gigas* is mainly made of calcite and organic matrices, including phosphorous.

The elemental composition of the semi-thin sections through the gastric mill was different from that of pereonite (Fig. 1Figs 2b and,2c, S1b and S1c Figs). The tissues surrounding the gastric mill showed similar elemental content to that of the pereonite. On the other hand, a strikingly different composition was found on the gastric mill itself (Fig. 1Fig 2c)[29][21] which appeared to form a bilayered structure with an inner layer composed of carbon, nitrogen, oxygen, sodium, magnesium, and phosphorous, while the outer layer was composed of carbon, nitrogen, oxygen, sodium, calcium, and aluminum-like signal at 1.48 keV (Fig 1). Magnified EDS maps showed the thickness of the outer layer to be ~1 µm.

EPMA analysis was also performed using similar sections of *H. gigas* (Fig 3a)to correctly assign the X-ray signals at 1.48 keV (Fig. 2). Qualitative WDS spectrum of the outer layer of the gastric mill showed two peaks around 1.48 keV: 1.481 and 1.527 keV (Fig 3b top). Aluminum and bromine are the two possible candidates of for these peaks: L-line of bromine appears at 1.293 and 1.480 kV while K-line of aluminum appears at 1.486 keV[22][10]. The intensity around 1.48 keV can therefore be explained without aluminum being present, indicating that the aluminum-like signals we have observed could in fact be bromine (S3 Fig). The presence of bromine was further supported by aAnother signal at 11.92 keV was also observed, corresponding to the K-line of bromine (Fig 3b bottom), confirming that the aluminum-like signals we have observed is in fact bromine. The intensity around 1.48 keV can thus be explained without aluminum. EPMA maps showed directional bromine localization toward the side exposed to seawater, similar to that observed in the EDS maps.

Fig 3. X-ray spectra and elemental maps of a semi-thin section of the gastric mill in *Hirondellea gigas*. a) SEM image of the mapped area. b) Point analysis at the red point in

Formatted: Font: Bold

Formatted: Font: Helvetica, Font color: Black

Formatted: Font color: Black

Formatted: Font: Helvetica, Font color: Black

Formatted: Font: Helvetica, Font color: Black

Formatted: Font: Bold

Formatted: Normal, Position: Horizontal: Left, Relative to: Column, Vertical: In line, Relative to:

(a). c) WDS elemental maps of the orange rectangle in (a). Note that Al K contrast is significantly weaker than that of Br K.

The elemental colocalization was further confirmed by calculating~~ed~~ ~~by~~ cross-~~-~~correlation ~~analysis~~ factor of the whole image (~~Table~~ S1 Table).[26]~~[16]~~ A h~~H~~igh ~~positive cross correlation values are obtained when two elements have similar relative intensity in any position, i.e. colocalized, and becomes zero when two random noise images were correlated. Note that correlation with carbon is not reliable, as the surrounding resin and the Kapton tape below the sample contain large amount of carbon which overshadows the carbon in the cross-sectioned tissues.~~ High correlation was observed between Ca-O-F-Mg-P, with correlation values ranging from 0.29 to 0.70. On the other hand, the correlation values of bromine against calcium-colocalizing elements ~~was~~ were less than 0.1, indicating that the bromine layer is separated from the calcium-based minerals. Quantitative X-ray analysis showed the absence of calcium on the bromine-rich outer layer (~~Fig. S~~S3c Fig~~3~~). The l~~L~~ow concentration of bromine and the coexistence of fluorine and magnesium were also supported by the quantitative analysis (~~Fig. S~~S3 Fig~~3~~).

Similarly, EPMA maps of the pereonite were taken to explore elements of the exoskeleton (~~Fig. 3~~Fig 4, ~~Table~~ S2 Table). Cross-~~-~~correlation values indicated the high colocalization of Ca-O-Mg-P ~~by the~~with correlation values ranging from 0.15 to 0.70. The correlation of calcium-colocalizing minerals ~~were~~ was small due to low X-ray counts. The c~~C~~orrelation of bromine to calcium-colocalizing minerals ~~were~~ was less than 0.1 except for oxygen, denying strong bromine colocalization. Quantitative analysis clearly showed the absence of either bromine or aluminum (~~Fig. S~~S3b and S3c Figs~~3~~).

Fig 4. SEM images and elemental maps of a semi-thin section of the pereonite in *Hirondellea gigas*. a) Low-magnification SEM image. b) High-magnification SEM image of the red rectangle in (a). c) WDS elemental maps of the orange rectangle in (b).

EDS investigation of the dissected specimen

To further explore the localization of bromine in *Hirondellea gigas*, a dissected individual was freeze-dried and observed under SEM/EDS. EDS maps showed that the hard tissues in the *H. gigas* ~~is~~ are mainly composed of carbon, nitrogen, oxygen, phosphorous, and calcium as major elements, and fluorine, sodium, magnesium, chlorine, and bromine as minor elements.

Formatted: Font color: Black

Formatted: Font: Bold

Formatted: Heading 2

Formatted: Normal, Position: Horizontal: Left, Relative to: Column, Vertical: In line, Relative to:

Since bromine accumulation was detected on the sclerotized spines of t~~T~~he gastric mill by EPMA, we first examined the gastric mill. The gastric mill exhibited ~~exhibited~~ several large, sclerotized spines and numerous fine filter setae, the latter not well observed in thin sections (~~Fig. 4~~Fig 5, ~~Fig. S4~~S4a and S4b Figs). The dimensions of the spines ~~was~~ were found to be typically 100 μm in length and 15–25 μm in diameter (~~Fig. 4~~Figs 5a and~~,~~ 5b, S4c and S4e Figs). Fine filter setae around the sclerotized spines were simple and covered by adsorbents, presumably mucus (~~Fig. 4~~Figs 5c, S4d and S4f Figs). Elemental maps of Br K indicated an accumulation of bromine on the spines (~~Fig. 4~~Fig 5d, ~~Table~~ S3 Table). Line profile analysis of the Br K line showed that the bromine intensity was 12–13 at the root and 15 at the tip, indicating preferential bromine accumulation at the tip (~~Fig. 4~~Fig 5e). The presence of weak calcium signals, combined with EPMA results ~~above~~Fig 3), point to a bromine-rich and calcium-poor organic layer coating a calcium-rich inorganic layer. Although the intensity was lower, bromine accumulation was also observed on the filter setae (S4h Fig). The pterocardiac ossicle itself at the base of the spines ~~are~~ is made of carbon, oxygen, magnesium, phosphorous, and calcium, while bromine was not observed ~~with~~ in significant concentrations (S4h Fig).

Fig 5. SEM/EDS images of the gastric mill of *Hirondellea gigas*. a) low-magnification SEM image, b) magnified image of the spine, yellow rectangle in part (a), c) magnified image of the filter setae, orange rectangle in part (a), d) EDS elemental mapping and sum spectrum of the red rectangle in part (a). e) Line profile of the Br K intensity in EDS map along with the longitudinal axis of the ossicle spines. Profiles were shifted to the intensity direction for visibility, and average ± standard deviation of the intensity of 60 μm from root and 50 μm from the tip are shown in green and blue bars, respectively. The line profile was measured with the line width of 10 μm.

Because fine setae on the gastric mill also accumulated bromine, mouth parts around the gastric mill with fine setae, namely maxilla 2, maxilliped, and mandible, were analyzed by SEM/EDS (Fig 6, S5–S7 Figs). Bromine accumulation was also observed on the setae of these mouth parts, specifically the robust setae ~~(Fig. 5, Fig. S5–S7)~~. ~~The clearest~~Bromine accumulation was clearest~~seen~~ on maxilla 2 (~~Fig. 5~~Figs 6a and 6b~~, Fig. S~~ S5 Fig~~5~~). The maxilla itself was mainly composed of calcium carbonate together with magnesium and phosphorous, and the bristles were found on the surface, where they are exposed to seawater (Fig 6c, S5e and S5f Figs). Three types of setae were present on ~~the~~ maxilla 2 (~~Fig. 5~~Figs 6a~~,~~ and 6b). One was simple robust setae with a diameter of 10–20 μm and a length of 150–350 μm, occurring on the distal part of ~~the~~ maxilla 2 (S5b right Fig); a second type of robust setae was similar in size but plumose with many fine setae (diameter of 0.6–2.5 μm and length of 35–50 μm, Fig S5b bottom); a third type was fine,

Formatted: Font: Bold

Formatted: Highlight

Formatted: Normal, Position: Horizontal: Left, Relative to: Column, Vertical: In line, Relative to:

setulose setae 0.5–1.5 µm in diameter with a length of 50–150 µm (S5b top, S6c, and S6d Figs). EDS maps showed that bromine ~~is~~ was accumulated on both types of robust setae with carbon, nitrogen, oxygen, and sulfur, but not on the finer, setulose setae which ~~was~~ were rich in phosphorous and calcium (~~Fig. 5~~Fig 6c, ~~Table~~ S3 Table). Similarly, selective bromine accumulation on the robust setae was found in both the maxilliped and the mandible (~~Fig. S~~S6~~6–~~ and S7 Figs~~7~~, ~~Table~~ S3 Table). Since the tissues were not washed excessively in order to prevent damage to potentially fragile setae, sodium chloride particles were observed between the setae of maxilliped, and bromine was not observed on the particles (S6c and S6e Figs).

Fig 6. SEM/EDS images of maxilla 2 of *Hirondellea gigas*. a) low-magnification SEM image, b) magnified image of the yellow rectangle in part (a), c) EDS elemental mapping and sum spectrum of the red rectangle in part (a).

Formatted: Font: Bold

These accumulations of bromine raised the possibility that bromine is widely present on setae across the whole amphipod, and we therefore proceeded to investigate other parts of the body where setae are present, including antenna, pleopods, and uropods. Antenna, fine parts most close to the mouth, ~~Microscopic observation of the antenna~~ (~~Fig. 6~~Fig 7, ~~Fig. S8~~ S8a Fig) also ~~revealed~~ contained the presence of bromine specifically on the setae extending from the distal parts of each article. These setae were simple and fine, 50–80 µm in length and 1–3 µm in diameter (~~Fig. 6~~Figs 7a~~,~~ and 7~~-~~b, S8b and S8d Figs). Setae on the distal end of article 1 of the antennae are, however, barbed and have short barbs projecting out laterally (S8c and S8e Figs). Elemental maps and point analysis revealed bromine accumulation on the setae, free of magnesium, phosphorous, and calcium that were found on the articles themselves (~~Fig. 6~~Fig 7c, ~~Table~~ S3 Table). The setae were characterized by high carbon, nitrogen, oxygen, and sulfur contents, while magnesium and phosphorous were less significant.

Fig 7. SEM/EDS images of an antenna of *Hirondellea gigas*. a) low-magnification SEM image, b) magnified image of the yellow rectangle in part (a), c) EDS elemental mapping and sum spectrum of the red rectangle in part (a).

Formatted: Font: Bold

Microscopic observations of pleopods ~~Observations of the pleopods~~ revealed bromine accumulation on robust setae as well as the outer surface of the pleopods themselves (~~Fig. 7~~Fig 8, ~~Fig. S9~~ S9 and S~~S~~10 Figs). Two types of setae were present, with the robust setae being simple and approximately 4-5 µm in diameter, and elongate fine setae being approximately 0.3-1.2 µm in diameter and about 3 mm in length (~~Fig. 7~~Figs 8a~~,~~ and 8~~-~~b, S9b and S9c Figs). Bromine was found to be accumulated only on the robust setae

Formatted: Normal, Position: Horizontal: Left, Relative to: Column, Vertical: In line, Relative to:

(Fig 8c, S9d Fig). Unlike other parts of the body, some bromine accumulation was also detected on the surface of the pleopods (Fig. 7Figs 8c, – S10 Fig, Table S3 Table).

Fig 8. SEM/EDS images of a pleopod of *Hirondellea gigas*. a) low-magnification SEM image, b) magnified image of the yellow rectangle in part (a), c) EDS elemental mapping and sum spectrum of the red rectangle in part (a).

Formatted: Font: Bold

Uropods possess short distolateral robust setae (Fig 9, S11a Fig), andOn the uropods, we also found bromine accumulating on the distolateral robust setae, which were approximately 10–15 μm in diameter and 80–100 μm in length (Fig. 8Fig 9b, Fig. S11S11b and S11c Figs). The overall surface of the uropods including the setae lacked bromine accumulation, unlike in the pleopods (Fig. 8Fig 9c, S11d Figd, Table S3 Table).

Fig 9. SEM images and EDS maps of dissected dry uropod of *Hirondellea gigas*. a) Low-magnification image. b) High-magnification image of the yellow square in (a). c) Sum spectrum and EDS maps of the red square in (a). The sum spectra at 10–15 kV were magnified and displayed in the inset above with the same energy scale for clarity.

Formatted: Font: Bold

Since EPMA investigation showed an absence of bromine on the pereonite, suggesting that bromine could be absent on the surface of the dorsal armature of *H. gigas*. We therefore performed SEM/EDS analysis on the coxal plates and pereonitesThe dorsal external armature of *H. gigas*, on the other hand, did not exhibit any specific bromine accumulation, from our observations of the coxal plates (Fig. Fig 10, 7, Fig. S12) and the pereonites (Fig. S13S12a Fig). Both coxal plates and pereonites were composed of hexagonal elements approximately 30 μm in size, which were more clearly visible in on the coxal plates (Fig. Fig 107b, S12c Fig). High-magnification images of the dorsal armature revealed numerous bead-like structures arranged in nearly parallel lines transversing the body, with the typical spacing between the lines being 669 ± 184 nm (N = 36, S12d Fig). Elemental mapping of showed a lack ofdid not exhibit any specific localization of bromine related to the surface structure in these parts of the exoskeleton, as was also indicated in area analyses (Fig. 7Fig 8c, , S12e Fig, Table S3 Table); though, while the sum spectra showed indicated small amountsmall amounts of bromine.

Fig 10. SEM/EDS images of a coxal plate of *Hirondellea gigas*. a) low-magnification SEM image, b) magnified image of the yellow rectangle in part (a), c) EDS elemental mapping and sum spectrum of the red rectangle in part (a).

Formatted: Font: Bold

Formatted: Normal, Position: Horizontal: Left, Relative to: Column, Vertical: In line, Relative to:

Discussion

Elemental analysis using electron microscopy

We took several precautions during sample preparation to avoid artificial X-ray signals from aluminum outside the sample, which is a very common element and contaminant in electron microscopy.[23] The use of Kapton/carbon reduces the emission of BSE from the substrate, preventing secondary electrons from the SEM chamber (SE3) as well as X-rays from the chamber made of aluminum alloy.[21] The amphipod tissue was mounted as carbon-coated flat semi-thin sections (unlike the bulk specimen in ref. [20]) due to three benefits in X-ray analysis. Firstly, the spatial resolution of a semi-thin section is higher than that of a bulk specimen because X-ray generation distant from the irradiated position by electrons scattered inside the sample is suppressed. Secondly, the charge-up of the thin section is minimized, as primary electrons irradiated to the thin section are not retained in the sample, but instead penetrate through to the underlying layer, where they are neutralized by the conductive coatings.[30] In the case of bulk samples with an insulative interior, as is the case with biological materials, accumulated charges bend the electron trajectory to decrease spatial resolution. Finally, using semi-thin sections ensure the electrons and X-rays are emitted homogeneously from a flat surface, which is desirable for quantitative analysis. Surface roughness causes shading of X-rays to decrease signal intensities of low-energy X-rays.[31] Without these precautions, total X-ray counts from each position do not necessarily reflect the sample's true elemental composition. We used the counts per second (cps) images, corresponding to total X-ray counts from each pixel, as our criteria to distinguish whether the signal originated from the sample or bremsstrahlung.

After performing the analyses with these precautions, ~~O~~our results showed that what was previously thought to be aluminum signals from *Hirondellea gigas*[20][8] was ~~actually~~ most likely bromine. This misassignment of elements can be traced back to issues with the sample preparation and selection of electron microscopic imaging conditions in Kobayashi *et al*.[20][8] Although high-resolution imaging can be performed without any coatings by using low-landing voltage techniques under low beam current,[32,33][22,23] EDS measurements typically require doubled voltage of maximum characteristic X-ray energy of ~~target~~ analyzing elements. In this situation,~~,~~ ~~and~~ a large beam current is required for X-ray emission sufficient for elemental mapping. The spectrum acquisition condition is prone to cause charge-up, as electrons can penetrate deep into the insulative organic specimen. In addition, internalized high-energy electrons can escape from the side of curved surfaces, resulting in enhanced counts of low-angle ~~backscattered electrons~~BSE – thus the total intensity of X-rays from the SEM chamber and substrate at the sample edge,

Formatted: Font color: Black

Formatted: Font color: Black

Formatted: Font color: Black

Formatted: Font color: Black

Formatted: Font color: Black

Formatted: Font color: Black

Formatted: Font color: Black

Formatted: Font color: Black

Formatted: Normal, Position: Horizontal: Left, Relative to: Column, Vertical: In line, Relative to:

which corresponds to the previous results where aluminum signals were mostly detected on~~in~~ the sample edges (~~fig. 1~~Fig 1 in ref. [20]~~8~~). The use of aluminum stubs and aluminum-reinforced double-sided carbon tape, as in ref. [20]~~8~~, is not recommended for aluminum detection for these reasons. Silicon is also an element to be ~~cautious~~ used with caution~~about~~, because the EDS detector window is made of silicon, which can be excited either by ~~backscattered electrons~~ BSE ~~and~~ or incoming high-energy X-rays, the latter of which is known as the escape peak.

One criterion to distinguish whether the EDS signals in map images truly originate~~s~~ from elemental localization or are artefacts, is to compare the image with total X-ray counts at each pixel, or counts per second~~s~~ (cps) images. Uncorrected X-ray maps ~~plot~~ display X-ray intensities of a given X-ray energy range at each position, including bremsstrahlung X-rays that~~, which~~ ~~is~~are in correlation with~~ed to~~ the total emitted X-rays. When the relative intensities of X-ray maps and the cps map of the area concerned are similar in ratio, the element localization is in doubt. Note that conventional X-ray corrections, such as ZAF correction using atom number (Z), X-ray absorption (A) and fluorescence excitation (F) ~~is~~ are not ~~always~~ necessarily accurate ~~suitable~~ for EDS analysis of non-flat specimens. Increasing the beam energy is another method to distinguish among elements. In the particular case of the aluminum K-line (1.486 keV), the bromine L-line (1.480 keV) and M-lines of two lanthanides absent in animals (thulium and ytterbium)[24]~~12~~ can overlap with it at the energy resolution of EDS (typically ~130 eV). By using high-energy electron beams, the bromine K-line (11.92 keV) can be detected without overlapping with other elements. High-energy X-rays have ~~less~~ lower intensities than low-energy ones, and the use of high-sensitivity detectors is desired. The use of semi-thin sections, comparison of X-ray maps and cps maps, irradiation of high-energy electron beam, and the use of the high-sensitivity detector in our analytical methods ensured that we ~~can~~ were likely to avoid such ~~pitfalls~~ artifact pitfalls~~of artifacts~~.

In our measurements, sea salt sometimes appeared on the surface, which is an artefact ~~during~~ of the drying process,~~;~~ because we did not wash the tissues thoroughly to prevent removal of fragile parts. However, sea salt only appeared as granules, not as surface coatings, judging from the amount of Na and Cl being far lower than that of Br on fine setae (S4–S8 Figs). This indicates that ~~the sea~~ salt ~~does~~ did not affect the amount of bromine because the molar amount of bromine is less than 1% of that of sodium, the main content of the sea salt.

Origin of bromine and its biological implication

Bromine is the eighth most abundant element~~s~~ in the ocean after chlorine, sodium, magnesium, sulfur, calcium, potassium, and carbon.[34]~~13~~ Although the explicit amount of bromine in the Izu-Ogasawara Trench ~~is~~ has not been reported,[35]~~24~~ the relative amount

Formatted: Font color: Black

Formatted: Font color: Black

Formatted: Font: Helvetica, Font color: Black

Formatted: Font: Helvetica, Font color: Black

Formatted: Font: Helvetica, Font color: Black

Formatted: Normal, Position: Horizontal: Left, Relative to: Column, Vertical: In line, Relative to:

of bromine is expected to be similar to that of standard seawater. The accumulation of bromine could be a result of 1) solidification and concentration from sea salt during sample preparation, 2) exogenous adhesion on the amphipod's exoskeleton while the amphipod was still alive, or 3) active accumulation and secretion by the amphipod itself. We observed solidified sea salt on the setae of maxilliped but these were without bromine coexistence (S6c–S6e Figs), rejecting the bromine signals being results of artefacts from sample preparation. Exogenous adhesion is also considered to be unlikely since chlorine, which has similar chemical properties as a water-dissolved anion and being more abundant in seawater than bromine, was absent on the setae. ~~A possibility is the simple adhesion of bromine, but the absence of chemically similar chlorine denies this possibility.~~ Rather, it is more plausible to consider that bromine was secreted by the amphipod, most preferentially to the gastric mill but also to the robust setae on various parts of the body, as well as the surface of the pleopods.

Accumulation of bromine has been also found on other marine organisms~~,~~~~.~~ and bromine-rich aromatic small molecules have been isolated~~In terms of small molecules extracted~~ from marine organisms~~,~~. For examples, the marine snail *Bolinus bandaris*~~–~~ (a~~has been~~ historically important ~~as the~~ source of Tyrian purple) secretes ~~whose structural isomers have~~ 6-bromo-3-indolinone ~~structure~~derivatives,[36]~~[25]~~ the tripeptide barettin from the marine sponge *Geodia barretti* has dehydrogenated 6-bromotryptophan,[37]~~[26]~~ and marine bacteria such as *Pseudomonas bromoutilis* produces ~~the~~ pentabrominated phenylpyrrole~~ is found~~, ~~as~~ which is an antibiotic compound ~~from marine bacteria~~.[38,39]~~[27,28]~~ Bromine is attached on aromatic rings in all cases. There are also ~~cases~~ examples of bromine ~~in~~ being accumulated on the hard parts of marine organisms whose molecular structure has not been identified, such as the fish otolith ~~which~~ containing bromine and chlorine colocalized as minor elements.[40]~~[29]~~ Such cases of bromine in hard parts are also known from at least two cases where the hard parts have predation or feeding-related functions. In the crab *Pachygrapsus crassipes*, bromine is enriched on the tips of its calcified claws, and mechanical tests have shown that this makes the tips less hard ~~but~~, yet more fracture-resistant.[16,17]~~[14,30]~~ Another case is the zinc-based jaws of the marine polychaete worm *Nereis virens* that also contain chlorine, manganese, and iodine; brominated amino acids, such as histidine, might bind to metals strongly to prevent dissolution. [2,3]~~[2,3]~~

The hadal amphipod *H. gigas* is unique among these animals in that the bromine does not coexist with other halogens and it is poor in other transition metals within the 1-um thick organic coatings containing bromine. In *H. gigas*, bromine is expected to reinforce the gastric mill to crush food ~~like~~ in the same manner as reinforcement occurs in the jaws of other animals~~' jaws, considering its biological role in crushing its food~~. The localization of bromine on the sclerotized spines and the lack of it in the calcified ossicles

Formatted: Font: Helvetica, Font color: Black
Formatted: Font: Helvetica, Font color: Black
Formatted: Font color: Black
Formatted: Font: Helvetica, Font color: Black
Formatted: Font color: Black
Formatted: Font: Helvetica, Font color: Black
Formatted: Normal, Position: Horizontal: Left, Relative to: Column, Vertical: In line, Relative to:

itself is likely beneficial in that bromine renders the spines less susceptible to breaking, in order to maintain its sharpness and function in food sorting. The ossicles are used for actual crushing, and as such they areit is beneficial for them to be calcified to increase the hardness. The same has been suggested for the function of the forceps-like, bromine-enriched, crab claw tips versus the rest of the claw used for crushing, which is calcified and lacks bromine.[16][14] The form of bromine in the gastric mill and setae remains unclear. The coexisting nitrogen and sulfur with bromine indicate the presence of amino acids, which may imply that the bromine is incorporated in aromatic amino acids, such as phenylalanine and tryptophan, in which case the bromine is most likely secreted by *H. gigas*. The function of bromine on the setae of *H. gigas* remains unclear at this point, although it may be secreted in a similar manner to the spines in the gastric ossicles.

Formatted: Font color: Black

Using calcite as the exoskeleton in depths deeper than the CCD (typically <5000 m) typically results in dissolution, and how *H. gigas* can retain its calcite armature requires a reconsideration now that it has become clear that it does not possess an 'aluminum shield' like previously suggested.[20][8] EPMA showed the existence of magnesium in the calcite layer with the Mg/Ca ratio being around 2–20% (Fig. S3S2 Fig). The absence of chlorine in the dried specimen (S4–S12 Fig) indicates that the magnesium is not from the remaining seawater salt, but is within the exoskeleton. Water solubility of calcite decreases by partial slight substitution of calcium with magnesium, i.e. $(Ca,Mg)CO_3$,[41,42][31,32] and the substitution would partially prevent carbonate dissolution below the CCD. Phosphorous may be present as calcium phosphate, contributing to the stabilization of calcium carbonate mineralsThough phosphorous was also present in considerable amount, its role is ambiguous as XRD did not provide the evidence of calcium phosphate minerals..[5,6,10]–

Formatted: Font color: Black

Formatted: Font: Helvetica, Font color: Black

Formatted: Font color: Black

Conclusion

In summary, we revealed the presence of bromine and magnesium in the hadal amphipod *Hirondellea gigas*, and showed that it does not actually possess an 'aluminum shield' as previously thought, through careful prevention of aluminum artefacts. Instead of the 'aluminum gel' shielding, the use of magnesium-substituted calcite may be the key for the amphipod to survive without demineralization below the CCD. A Hhigh concentrations of bromine was were found on the sclerotized spines in the gastric mill, forming a 1 μm-thick coating layer on mineralized tissue with a relatively high concentration of nitrogen. The bromine-coating on the gastric mill spines is speculated to have the function of maintaining a sharp tip for food processing, similar to what has been suggested for crab claw tips. Our study serves as a reminder of the importance and necessity of appropriate sample preparation and careful analysis of the signals in EDS

Formatted: Normal, Position: Horizontal: Left, Relative to: Column, Vertical: In line, Relative to:

~~analysis~~analyses, as well as the importance ~~to~~ of analyzing samples~~e~~ at different electron energies when performing microscopic studies of 'elemental anatomy'.

Formatted: Heading 1

Acknowledgements

The authors thank the Captain and crew of R/V *Yokosuka* during expeditions YK19-11 (cruise PI: Akinori Yabuki, JAMSTEC) for their great support of the scientific activity. All scientists on-board the expeditions are gratefully acknowledged for their tireless work, especially Heather Ritchie Parker who assisted with the preservation of the amphipod specimens. We also thank Kana Nagashima (JAMSTEC) for her kind offer for XRD analysis. ~~We thank JSPS for financial support with a [Grant-in-Aid for Young Scientists Grant Number JP19K15379] to S.O. and a [Grant-in-Aid for Scientific Research Grant Number 18K06401] to C.C. and H.K.W.~~

~~Author contributions~~

Formatted: Normal

~~S.O., C.C., and K.T. designed the experiments. N.I. collected and preserved the amphipod on-board the research vessel. S.O. performed sample preparation and electron microscopic observation. S.O. and N.I. performed XRD analysis. S.O., H.K.W., and C.C. dissected the amphipod. All authors contributed to the writing of this manuscript and approved its submission and publication.~~

~~Conflict of interest~~

Formatted: Normal

~~The authors declare no competing financial interest.~~

~~Supporting Information~~

Formatted: Normal

~~Additional figures of SEM images, EDS maps, EDS spectra, XRD data, and tables of cross-correlation data in EPMA, and elemental analyses based on EPMA and EDS.~~

Formatted: Heading 1

~~Figure Legends~~

Formatted: Heading 1

~~Figure 1. EDS maps of semi-thin sections of *H. gigas*. (a) transverse section through a subsample of pereonite, (b) transverse section of the cephalic region showing the gastric mill, (c) magnified images of the gastric mill, the red rectangle area in (b). Images from the left are SEM~~

Formatted: Normal, Position: Horizontal: Left, Relative to: Column, Vertical: In line, Relative to:

images at (a,b) 1 kV and (c) 10 kV, total X-ray counts (grayscale), phosphorous (green), calcium (blue), and aluminum-like signals around 1.48 keV (silver). EDS spectra were acquired at 10 kV, thus the K-line of bromine was not observed.

Figure 2. X-ray spectra and elemental maps of a semi-thin section of the gastric mill in *Hirondellea gigas*. (a) SEM image of the mapped area. (b) Point analysis at the red point in (a). (c) WDS elemental maps of the orange rectangle in (a). Note that Al K contrast is significantly weaker than that of Br K.

Figure 3. SEM images and elemental maps of a semi-thin section of the pereonite in *Hirondellea gigas*. (a) Low-magnification SEM image. (b) High-magnification SEM image of the red rectangle in (a). (c) WDS elemental maps of the orange rectangle in (b).

Figure 4. SEM/EDS images of the gastric mill of *Hirondellea gigas*. A) low-magnification SEM image, b) magnified image of the spine, yellow rectangle in part (a),

Formatted: Normal, Position: Horizontal: Left, Relative to: Column, Vertical: In line, Relative to:

c) magnified image of the filter setae, orange rectangle in part (a), d) EDS elemental mapping and sum spectrum of the red rectangle in part (a). e) Line profile of the Br K intensity in EDS map along with the longitudinal axis of the ossicle spines. The line profile was measured with the line width of 10 μm.

Figure 5. SEM/EDS images of maxilla 2 of *Hirondellea gigas*. A) low-magnification SEM image, b) magnified image of the yellow rectangle in part (a), c) EDS elemental mapping and sum spectrum of the red rectangle in part (a).

Figure 6. SEM/EDS images of an antenna of *Hirondellea gigas*. A) low-magnification SEM image, b) magnified image of the yellow rectangle in part (a), c) EDS elemental mapping and sum spectrum of the red rectangle in part (a).

Figure 7. SEM/EDS images of a pleopod of *Hirondellea gigas*. A) low-magnification SEM image, b) magnified image of the yellow rectangle in part (a), c) EDS elemental mapping and sum spectrum of the red rectangle in part (a).

Formatted: Normal, Position: Horizontal: Left, Relative to: Column, Vertical: In line, Relative to:

Figure 8. SEM images and EDS maps of dissected dry uropod of *Hirondellea gigas*. (a) Low-magnification image. (b) High-magnification image of the yellow square in (a). (c) Sum spectrum and EDS maps of the red square in (a). The sum spectra at 10–15 kV were magnified and displayed in the inset above with the same energy scale for clarity.

Figure 9. SEM/EDS images of a coxal plate of *Hirondellea gigas*. A) low-magnification SEM image, b) magnified image of the yellow rectangle in part (a), c) EDS elemental mapping and sum spectrum of the red rectangle in part (a).

References

1. Meenakshi VR, Hare PE, Watabe N, Wilbur KM. The chemical composition of the periostracum of the molluscan shell. Comp Biochem Physiol. 1969;29: 611–620.

2. Broomell CC, Zok FW, Waite JH. Role of transition metals in sclerotization of biological tissue. Acta Biomater. 2008;4: 2045–2051.

3. Broomell CC, Mattoni MA, Zok FW, Waite JH. Critical role of zinc in hardening of Nereis jaws. J Exp Biol. 2006;209: 3219–3225.

Formatted: Font color: Black

Formatted: Normal, Position: Horizontal: Left, Relative to: Column, Vertical: In line, Relative to:

4. Brandao SN, Hoppema M, Kamenev GM, Karanovic I, Riehl T, Tanaka H, et al. Review of Ostracoda (Crustacea) living below the Carbonate Compensation Depth and the deepest record of a calcified ostracod. Progress Oceanogr. 2019;178. doi:10.1016/j.pocean.2019.102144

5. Luquet G. Biomineralizations: insights and prospects from crustaceans. Zookeys. 2012;176: 103–121.

6. Bentov S, Abehsera S, Sagi A. The Mineralized Exoskeletons of Crustaceans. In: Cohen E, Moussian B, editors. Extracellular Composite Matrices in Arthropods. Cham: Springer; 2016. pp. 137–163.

7. Becker A, Ziegler A, Epple M. The mineral phase in the cuticles of two species of Crustacea consists of magnesium calcite, amorphous calcium carbonate, and amorphous calcium phosphate. Dalton Trans. 2005;0: 1814–1820.

8. Bourget E. Barnacle shells: Composition, structure and growth. In: Southward AJ, editor. Barnacle Biology. London: Taylor & Francis; 1987.

9. Lowenstam HA, Weiner S. Phosphatic shell plate of the barnacle Ibla (Cirripedia): A bone-likestructure. Proc Nat Acad Sci. 1992;89: 10573–10577.

10. Bentov S, Aflalo ED, Tynyakov J, Glazer L, Sagi A. Calcium phosphate mineralization is widely applied in crustacean mandibles. Sci Rep. 2016;6: 22118.

11. Tynyakov J, Bentov S, Abehsera S, Yehezkel G, Roth Z, Khalaila I, et al. A crayfish molar tooth protein with putative mineralized exoskeletal chitinous matrix properties. J Exp Biol. 2015;218: 3487–3498.

12. Bentov S, Zaslansky P, Al-Sawalmih A, Masic A, Fratzl P, Sagi A, et al. Enamel-like apatite crown covering amorphous mineral in a crayfish mandible. Nat Commun. 2012;3: 839.

13. Neues F, Hild S, Epple M, Marti O, Ziegler A. Amorphous and crystalline calcium carbonate distribution in the tergite cuticle of moulting Porcellio scaber (Isopoda, Crustacea). J Struct Biol. 2011;175: 10–20.

14. Keyser D, Walter R. Calcification in ostracodes. Revista Española de Micropaleontología. 2004;36: 1–11.

15. Brandão SN, Hoppema M, Kamenev GM, Karanovic I, Riehl T, Tanaka H, et al. Review of Ostracoda (Crustacea) living below the Carbonate Compensation Depth and the deepest record of a calcified ostracod. Prog Oceanogr. 2019;178: 102144.

Formatted: Normal, Position: Horizontal: Left, Relative to: Column, Vertical: In line, Relative to:

16. Schofield RMS, Bailey J, Coon JJ, Devaraj A, Garrett RW, Goggans MS, et al. The homogenous alternative to biomineralization: Zn- and Mn-rich materials enable sharp organismal “tools” that reduce force requirements. Sci Rep. 2021;11: 17481.

17. Schofield RMS, Niedbala JC, Nesson MH, Tao Y, Shokes JE, Scott RA, et al. Br-rich tips of calcified crab claws are less hard but more fracture resistant: A comparison of mineralized and heavy-element biological materials. J Struct Biol. 2009;166: 272–287.

18. Kobayashi H, Hatada Y, Tsubouchi T, Nagahama T, Takami H. The Hadal Amphipod Hirondellea gigas Possessing a Unique Cellulase for Digesting Wooden Debris Buried in the Deepest Seafloor. PLoS ONE. 2012;7: e42727.

19. Kobayashi H, Nagahama T, Arai W, Sasagawa Y, Umeda M, Hayashi T, et al. Polysaccharide hydrolase of the hadal zone amphipods Hirondellea gigas. Biosci Biotechnology Biochem. 2018;82: 1123–1133.

20. Kobayashi H, Shimoshige H, Nakajima Y, Arai W, Takami H. An aluminum shield enables the amphipod Hirondellea gigas to inhabit deep-sea environments. PLoS ONE. 2019;14: e0206710.

21. Suri A, Pratt A, Tear S, Walker C, Kincal C, Kamber U, et al. Analysis and detection of low-energy electrons in scanning electron microscopes using a Bessel box electron energy analyser. J Elec Spec Rel Phen. 2020;241: 146823.

22. Bearden JA. X-ray wavelengths and x-ray atomic energy levels. Rev Mod Phys. 1967;39: 78–124.

23. Newbury DE. Mistakes encountered during automatic peak identification in low beam energy X-ray microanalysis. Scanning. 2007;29: 137–151. doi:10.1002/sca.20009

24. Cotruvo JA. The Chemistry of Lanthanides in Biology: Recent Discoveries, Emerging Principles, and Technological Applications. ACS Cent Sci. 2019;5: 1496–1506.

25. Luft JH. Improvements in epoxy resin embedding methods. J Biophys Biochem Cytol. 1961;9: 409–414.

26. Du K, Hochmeister K von, Phillipp F. Quantitative comparison of image contrast and pattern between experimental and simulated high-resolution transmission electron micrographs. Ultramicrosc. 2007;107: 281–292.

27. Cauchie H-M. Chitin production by arthropods in the hydrosphere. Hydrobiologia. 2002;470: 63–95.

Formatted: Normal, Position: Horizontal: Left, Relative to: Column, Vertical: In line, Relative to:

28. Sugawara A, Nishimura T, Yamamoto Y, Inoue H, Nagasawa H, Kato T. Self-Organization of Oriented Calcium Carbon- ate/Polymer Composites: Effects of a Matrix Peptide Isolated from the Exoskeleton of a Crayfish. Angew Chem Int Ed. 2006;45: 2876–2879.

29. Becker C, Dick JTA, Cunningham EM, Schmitt C, Sigwart JD. The crustacean cuticle does not record chronological age: New evidence from the gastric mill ossicles. Arthropod Structure and Development. 2018;5: 498–512.

30. Joy DC, Pawley JB. High-resolution scanning electron microscopy. Ultramicrosc. 1992;47: 80–100.

31. Postek MT, Vladár AE. Does Your SEM Really Tell the Truth?–How Would You Know? Part 4: Charging and its Mitigation. Proc SPIE Int Soc Opt Eng. 2015;9636: 963605.

32. Harano K, Okada S, Furukawa S, Tanaka H, Nakamura E. Formation of a polycrystalline film of donor material on PEDOT:PSS buffer induced by crystal nucleation. J Polym Sci Part B: Polym Phys. 2014;52: 833–841.

33. Gorgoll RM, Harano K, Nakamura E. Nanoscale Control of Polymer Assembly on a Synthetic Catalyst-Bilayer System. J Am Chem Soc. 2016;138: 9675–9681.

34. Millero FJ, Feistel R, Wright DG, McDougall TJ. The composition of Standard Seawater and the definition of the Reference-Composition Salinity Scale. Deep Sea Res Part Oceanogr Res Pap. 2008;55: 50–72.

35. Obata H, Nishioka J, Kim T, Norisuye K, Takeda S, Wakuta Y, et al. Dissolved iron and zinc in Sagami Bay and the Izu-Ogasawara Trench. J Oceanogr. 2017;73: 333–344.

36. Cooksey C. Tyrian purple: the first four thousand years. Sci Prog. 2013;96: 171–186.

37. Sölter S, Dieckmann R, Blumenberg M, Francke W. Barettin, revisited? Tetrahedron Lett. 2002;43: 3385–3386.

38. Lovell FM. The structure of a bromine-rich marine antibiotic. J Am Chem Soc. 1966;88: 4510–4511.

39. Burkholder PR, Pfister RM, Leitz FH. Production of a pyrrole antibiotic by a marine bacterium. Appl Microbiol. 1966;14: 649–653.

40. Arai N, Sakamoto W, Maeda K. Analysis of Trace Elements in Otoliths of Red Sea Bream Pagrus major. Fish sci. 1995;61: 43–47.

Formatted: Normal, Position: Horizontal: Left, Relative to: Column, Vertical: In line, Relative to:

41. Mucci A, Morse JW. The solubility of calcite in seawater solutions of various magnesium concentration, It = 0.697 m at 25 ° C and one atmosphere total pressure. Geochim Cosmochim Acta. 1984;48: 815–822.

42. Morse JW, Mucci A, Walter LM, Kaminsky MS. Magnesium interaction with the surface of calcite in seawater. Science. 1979;205: 904–905.

1. Meenakshi, V. R., Hare, P. E., Watabe, N. & Wilbur, K. M. The chemical composition of the periostracum of the molluscan shell. *Comp Biochem Physiol* 29, 611–620 (1969).

2. Broomell, C. C., Zok, F. W. & Waite, J. H. Role of transition metals in sclerotization of biological tissue. *Acta Biomater* 4, 2045–2051 (2008).

3. Broomell, C. C., Mattoni, M. A., Zok, F. W. & Waite, J. H. Critical role of zinc in hardening of Nereis jaws. *J. Exp. Biol.* 209, 3219–3225 (2006).

4. Brandao, S. N. *et al.* Review of Ostracoda (Crustacea) living below the Carbonate Compensation Depth and the deepest record of a calcified ostracod. *Progress Oceanogr.* 178, (2019).

5. Bourget, E. Barnacle shells: Composition, structure and growth. in *Barnacle Biology* (ed. Southward, A. J.) (Taylor & Francis, 1987). doi:10.1201/9781315138053.

6. Kobayashi, H., Hatada, Y., Tsubouchi, T., Nagahama, T. & Takami, H. The Hadal Amphipod Hirondellea gigas Possessing a Unique Cellulase for Digesting Wooden Debris Buried in the Deepest Seafloor. *PLoS ONE* 7, e42727–8 (2012).

7. Kobayashi, H. *et al.* Polysaccharide hydrolase of the hadal zone amphipods Hirondellea gigas. *Biosci. Biotechnol. Biochem.* 1–11 (2018) doi:10.1080/09168451.2018.1459178.

8. Kobayashi, H., Shimoshige, H., Nakajima, Y., Arai, W. & Takami, H. An aluminum shield enables the amphipod Hirondellea gigas to inhabit deep-sea environments. *PLoS ONE* 14, e0206710 (2019).

9. Suri, A. *et al.* Analysis and detection of low-energy electrons in scanning electron microscopes using a Bessel box electron energy analyser. *J. Elec. Spec. Rel. Phen.* 241, 146823 (2020).

Formatted: csl-entry

Formatted: Normal, Position: Horizontal: Left, Relative to: Column, Vertical: In line, Relative to:

10. Bearden, J. A. X-ray wavelengths and x-ray atomic energy levels. *Rev. Mod. Phys.* 39, 78–124 (1967).

11. Newbury, D. E. Mistakes encountered during automatic peak identification in low beam energy X-ray microanalysis. *Scanning* 29, 137–151 (2007).

12. Cotruvo, J. A. The Chemistry of Lanthanides in Biology: Recent Discoveries, Emerging Principles, and Technological Applications. *ACS Cent Sci* 5, 1496–1506 (2019).

13. Millero, F. J., Feistel, R., Wright, D. G. & McDougall, T. J. The composition of Standard Seawater and the definition of the Reference-Composition Salinity Scale. *Deep Sea Res Part Oceanogr Res Pap* 55, 50–72 (2008).

14. Schofield, R. M. S. *et al.* The homogenous alternative to biomineralization: Zn- and Mn-rich materials enable sharp organismal “tools” that reduce force requirements. *Sci. Rep.* 11, 1–23 (2021).

15. Luft, J. H. Improvements in epoxy resin embedding methods. *J. Biophys. Biochem. Cytol.* 9, 409–414 (1961).

16. Du, K., Hochmeister, K. von & Phillipp, F. Quantitative comparison of image contrast and pattern between experimental and simulated high-resolution transmission electron micrographs. *Ultramicrosc.* 107, 281–292 (2007).

17. Joy, D. C. & Pawley, J. B. High-resolution scanning electron microscopy. *Ultramicrosc.* 47, 80–100 (1992).

18. Postek, M. T. & Vladár, A. E. Does Your SEM Really Tell the Truth?–How Would You Know? Part 4: Charging and its Mitigation. *Proc SPIE Int Soc Opt Eng* 9636, 963605 (2015).

19. Cauchie, H.-M. Chitin production by arthropods in the hydrosphere. *Hydrobiologia* 470, 63–95 (2002).

20. Sugawara, A. *et al.* Self-Organization of Oriented Calcium Carbon- ate/Polymer Composites: Effects of a Matrix Peptide Isolated from the Exoskeleton of a Crayfish. *Angew. Chem. Int. Ed.* 45, 2876–2879 (2006).

21. Becker, C., Dick, J. T. A., Cunningham, E. M., Schmitt, C. & Sigwart, J. D. The crustacean cuticle does not record chronological age: New evidence from the gastric mill ossicles. *Arthropod Structure and Development* 1–15 (2021) doi:10.1016/j.asd.2018.07.002.

Formatted: Normal, Position: Horizontal: Left, Relative to: Column, Vertical: In line, Relative to:

22. Harano, K., Okada, S., Furukawa, S., Tanaka, H. & Nakamura, E. Formation of a polycrystalline film of donor material on PEDOT:PSS buffer induced by crystal nucleation. *J. Polym. Sci. Part B: Polym. Phys.* 52, 833–841 (2014).

23. Gorgoll, R. M., Harano, K. & Nakamura, E. Nanoscale Control of Polymer Assembly on a Synthetic Catalyst–Bilayer System. *J. Am. Chem. Soc.* 138, 9675–9681 (2016).

24. Obata, H. *et al.* Dissolved iron and zinc in Sagami Bay and the Izu–Ogasawara Trench. *J Oceanogr* 73, 333–344 (2017).

25. Cooksey, C. Tyrian purple: the first four thousand years. *Sci Prog* 96, 171–186 (2013).

26. Sölter, S., Dieckmann, R., Blumenberg, M. & Francke, W. Barettin, revisited? *Tetrahedron Lett* 43, 3385–3386 (2002).

27. Lovell, F. M. The structure of a bromine–rich marine antibiotic. *J. Am. Chem. Soc.* 88, 4510–4511 (1966).

28. Burkholder, P. R., Pfister, R. M. & Leitz, F. H. Production of a pyrrole antibiotic by a marine bacterium. *Appl Microbiol* 14, 649–653 (1966).

29. Arai, N., Sakamoto, W. & Maeda, K. Analysis of Trace Elements in Otoliths of Red Sea Bream Pagrus major. *Fisheries science* 61, 43–47 (1995).

30. Schofield, R. M. S. *et al.* Br–rich tips of calcified crab claws are less hard but more fracture resistant: A comparison of mineralized and heavy–element biological materials. *J Struct Biol* 166, 272–287 (2009).

31. Mucci, A. & Morse, J. W. The solubility of calcite in seawater solutions of various magnesium concentration, It = 0.697 m at 25 ° C and one atmosphere total pressure. *Geochim. Cosmochim. Acta* 48, 815–822 (1984).

32. Morse, J. W., Mucci, A., Walter, L. M. & Kaminsky, M. S. Magnesium interaction with the surface of calcite in seawater. *Science* 205, 904–905 (1979).

Supporting Information

S1 Fig. EDS maps of semi-thin sections of *H. gigas*. a) transverse section through a subsample of pereonite, b) transverse section of the cephalic region showing the gastric

Formatted: Normal, Position: Horizontal: Left, Relative to: Column, Vertical: In line, Relative to:

mill, c) magnified images of the gastric mill, the red rectangle area in (b). SEM images, total X-ray counts (cps), and elemental maps are shown, and the labels are denoted in the left top. 'Al' corresponds to the aluminum-like signal around 1.48 keV, that overlaps with bromine L-line, and thulium and ytterbium M-line. d–f) EDS sum spectra of the (d) pereonite, (e) cephalic region, and (f) gastric mill created from the EDS maps (a)–(c). SEM images in (a) were acquired at 1 kV, and other images and spectra were acquired at 10 kV.

S2 Fig. XRD spectrum of dried pereonite of *H. gigas*. Diffraction peak position in 2θ and corresponding crystal planes of calcite and α-chitin in parenthesis are shown.

S3 Fig. Quantitative point analysis of EPMA using semi-thin sections. a) SEM images of the gastric mill, b) SEM image of the pereonite, c) atomic ratio of the elements. K-line of the elements were used for the quantification. The large value of carbon and oxygen is ascribed to the conductive carbon coating and the underlying Kapton tape. Note that the amount of aluminum was <5% of that of bromine, and may contain some error because of the overlap of Al K-line with Br L-line, which is not completely separable under WDS condition (Fig. 2b). Nitrogen was also searched but not observed at any points (0.00), thus not shown.

S4 Fig. SEM images and EDS spectra of the gastric mill of *Hirondellea gigas*. a) low-magnification SEM image, b) magnified image of the red rectangle in (a), c) magnified image of the orange rectangle in b), d) magnified image of the blue rectangle in (b), e) magnified image of the yellow rectangle in (c), f) magnified image of the green rectangle in (d), g) EDS elemental mapping of the red rectangle in (a), h) EDS spectra of the yellow points in (a). Atom labels correspond to K-line except for Br*, which is L-line.

S5 Fig. SEM images and EDS spectra of the maxilla 2 of *Hirondellea gigas*. a) low-magnification SEM image, b) magnified image of the red rectangle in (a), c) magnified image of the orange rectangle in b), d) magnified image of the yellow rectangle in (c), e) EDS elemental mapping of the blue rectangle in (a), f) EDS spectra of the yellow points in (b). Atom labels correspond to K-line except for Br*, which is L-line.

S6 Fig. SEM images and EDS spectra of the maxilliped of *Hirondellea gigas*. a) low-magnification SEM image, b) magnified image of the red rectangle in (a), c) magnified image of the orange rectangle in b). Note that the solid between setae are unremoved sodium chloride, assigned based on EDS mapping in (e). d) EDS spectra of the yellow points in (b), e) EDS elemental mapping of the blue rectangle in (a). Atom labels correspond to K-line except for Br*, which is L-line.

Formatted: Normal, Position: Horizontal: Left, Relative to: Column, Vertical: In line, Relative to:

S7 Fig. SEM images and EDS spectra of the mandible of *Hirondellea gigas*. a) low-magnification SEM image, b) magnified image of the red rectangle in (a), c) magnified image of the orange rectangle in b), d) magnified image of the yellow rectangle in (c), e) EDS maps of the blue rectangle in (a), f) EDS spectra of the yellow points in (b). Atom labels correspond to K-line except for Br*, which is L-line.

S8 Fig. SEM images and EDS spectra of the antenna of *Hirondellea gigas*. a) low-magnification SEM image, b) magnified image of the red rectangle in (a), c) magnified image of setae from the article in the yellow rectangle in (b), d) magnified image of the orange rectangle in (a), e) magnified image of short barbs in the green rectangle in (c), f) EDS elemental mapping of the blue rectangle in (a), g) EDS spectra of the yellow points in (a). Atom labels correspond to K-line except for Br*, which is L-line.

S9 Fig. SEM images and EDS spectra of the pleopod of *Hirondellea gigas*. a) low-magnification SEM montage image, b) magnified image of the red rectangle near the red triangle in (a) showing the two types of setae, c) magnified image of the yellow rectangle in b), d) EDS spectra of the yellow points in (a). Atom labels correspond to K-line except for Br*, which is L-line.

S10 Fig. SEM/EDS mapping of the pleopod of *Hirondellea gigas*. a) low-magnification SEM montage image, which is a part of Fig. S8, b) EDS elemental mapping of the blue rectangle in (a).

S11 Fig. SEM images and EDS spectra of the uropod of *Hirondellea gigas*. a) low-magnification SEM image, b) magnified image of the red rectangle, left middle in (a), c) magnified image of the orange rectangle in b), d) EDS spectra of the yellow points in (b), e) EDS elemental mapping of the blue rectangle in (a). Atom labels correspond to K-line except for Br*, which is L-line.

S12 Fig. SEM images and EDS spectra of the pereonite of *Hirondellea gigas*. a) low-magnification SEM image, b) magnified image of the red rectangle, left middle in (a), c) magnified image of the orange rectangle in (b) showing the hexagonal elements, d) magnified image of (b) showing the bead-like structure, e) EDS sum spectra of (e). The surface was elementally homogeneous and the EDS maps are not shown. Atom labels correspond to K-line except for Br*, which is L-line.

Formatted: Normal, Position: Horizontal: Left, Relative to: Column, Vertical: In line, Relative to:

S1 Table. Cross correlation of EPMA elemental maps of semi-thin section of the pereonite in Fig. 2. Top 10 values are shown in bold for visibility.

S2 Table. Cross correlation of EPMA elemental maps of semi-thin section of the gastric mill in Fig. 3. Top 10 values are shown in bold for visibility.

S3 Table. Elemental ratios of EDS point/area analyses in Fig. S4–13. GM, gastric mill; Mxp, maxillipet; Md, mandible; Mx2, maxilla 2; An, antenna; Pl, pleopod; Ur, uropod; Cx, coxal plate; Pe, pereonite. Values are reported by the atomic ratio ± errors in per cent. Note that the spectra were acquired under non-optimal condition, i.e. curved surface, and may contain large errors in the relative values. Area analysis was performed on Cx and Pe.

Formatted: Normal, Position: Horizontal: Left, Relative to: Column, Vertical: In line, Relative to:

The reviewer's comment is shown in red letters.

Reviewer #1: I read your paper with great interest. The authors used detailed image analysis by electron microscopy and X-ray measurements on amphipod samples collected from the difficult-to-reach ultra-deep sea, and found that amphipods do not have an "aluminum shield" as previously thought, but accumulate bromine and magnesium. We believe that these new findings will greatly contribute to the development of deep-sea biology. This paper is judged to be of sufficient value for publication in PlosOne.
Thank you very much for the positive comments.

Reviewer #2: See attached documents provided.
Review of "Unusual bromine enrichment in the gastric mill and setae of the hadal amphipod Hirondellea gigas" by Okada, et al.

Review of "Unusual bromine enrichment in the gastric mill and setae of the hadal amphipod *Hirondellea gigas*" by Okada, et al.
I have reviewed the ms by Okada, et al, regarding the absence of an aluminum gel shield in the deep-water amphipod, *H. gigas* and the presence of bromine, instead, demonstrating the importance of careful and proper SEM analyses of samples for 'elemental anatomy.' I found the manuscript (ms) of interest and of potential interest to the PLOS One audience of readers. However, although the work appears to be thorough and meticulously done, demonstrating careful analytical rigor, I have, nevertheless found the writing to be rather simplistic with faults in the logical flow of the paper. It appears more analogous to an undergraduate laboratory report, than a ms for publication, and for the most part, is poorly prepared.
For these and the following reasons, I do not recommend the publication of this work until it has been carefully recrafted and revised according to my comments below. I have provided a PDF of the text with my many small comments and edits, which I recommend the authors carefully consider and revise the ms accordingly. In addition, I am providing in this document, a list of both Minor and Major areas of revisions for consideration by the authors. I hope the comments provided facilitate the revising of the ms and are helpful to the authors. I believe that once revised, the paper would be of interest and use to other researchers with interests in the area of deep see arthropods and their anatomy and physiology.
Additionally, not having read the Instructions for Authors, I wonder why the ms does not have line numbers and why the many supplemental figures and tables were not provided for the purposes of review. Having line numbers facilitates a much easier review process for the reviewer and authors. I recommend that manuscripts NOT be sent to reviewers by the Editors

unless continuous line numbers have been included!!

Thank you for reading and evaluating our papers. The pdf you kindly attached was greatly helpful to revise our manuscript. I apologize for the absence of line numbers; which is my mistake. We have revised the manuscript following your comments and suggestions, and hope the revised manuscript is acceptable for publication.

Minor Revisions

There are a plethora of small grammatical errors in the text which I have endeavored to correct (as far as I could understand some of the sentences written). I recommend the authors have a native science writer review the text before resubmitting the revised ms to the journal (or any journal).

We have rewritten the manuscript and checked it with a native English speaker.

Abstract

L??? – the code EDS is used. However, EDS is never spelled out in the prior text. This needs to be spelled out in full the first time it is used, as here in this sentence.

We spelled out EDS accordingly. (L31)

Our results … signals in energy-dispersive X-ray spectroscopic analysis

Main Text

L??? – second para. The autors use the term "megafauna" for H. gigas. This is incorrect. H. gigas would be considered macrofauna, not megafauna. Please check the definitions of these.

We agree and changed to macrofauna. (L68)

L??? – the authors state, "The aluminum shield was expected to..." Who was it that was expecting this, and what is the citation/reference for this?

Those who found the 'aluminum shield' expected this. The reference 8 was added, and because this reference was cited continuously for three times, the next sentence was slightly modified.

However, their results were inconclusive… (L77)

1

L??? - ??? – "The aluminum shield...Challenger Deep." This sentence is long and convoluted. It makes little sense, and also needs a reference at the end to state who was expecting this gel shield.

We clarified the sentence and added a reference number. (L73)

Based on the combination of energy-dispersive X-ray analysis (EDS) under scanning electron microscopy (SEM) and aluminum extraction experiments from the sediment of Challenger Deep, Kobayashi et al. expected the aluminum shield to be in a form of gel.[8]

L??? - ??? – first part of first paragraph on pg. 3. "In order to confirm…artefacts." This sentence should be moved to the end of the Introduction section as a segway into the methods section.

We moved the sentence accordingly. (L74)

L??? - ??? – "There are two possibilities where aluminum…latter two being unused in animals." This section should be moved to the top of the first full paragraph on Pg. 3.

This sentence is now at the top of the first paragraph, after moving the sentence as suggested above. (L87)

L??? - ??? "Our detailed analysis revealed…harden the hard parts." This entire section belongs in the Results section, not in the introduction section. It is not part of the literature review or the justifications for the study.

We shortened the sentence to guide readers to our main goal of the bromine accumulation. (L94)

Here, we report our findings from detailed electron microscopy observations of *H. gigas*, revealing the absence of aluminum on the exoskeleton and instead the accumulation of bromine on hard tissues as the sclerotized spines of its gastric mill components, a rare example of preferentially secreted bromine in the hard parts of animals.

Methods

Amphipod Samples

L??? - ??? – it would be helpful to provide a map of the study location as Fig. 1.

We added the map, a photo of the bait trap, and the animal in Fig. S1. (L113)

L??? - ???. In this section, no mention is made of how many amphipod samples were actually collected in total, and neither is a total sample size mentioned in the Results.

Though over 200 amphipods were collected, only three were rapidly frozen using nitrogen slurry and used in the present study. Among the three frozen specimens, one was embedded in resin, and other two were dissected. This information is now added in Methods (L108)

Over 200 individuals (Fig. S1b) were collected, of which three individuals were used

for this study. The amphipods were immediately immersed …

Electron microscope instruments.

L??? – "...2k x 2K..." us the multiplication symbol (×), not the letter "x"

We revised the symbol accordingly. (L123, 163)

L??? – "The dehydrated amphipod was replaced with QY-1..." Is this really what the authors meant to say; that the actual animal was replaced with QY-1? I do not understand this. Should this mean something, such as "fluid from the amphipod was replaced by QY-1..."?

We simplified the resin embedding procedure as follows: (L131)

The dehydrated amphipod was immersed into QY-1…

L??? "...HighScore 3.0.5..." Is this hardware or software? It is not clear and not stated. Please clarify.

This is a software and we clarified accordingly (L171).

… performed on the software HighScore 3.0.5 (PANalytical).

L??? – "...maxilla (Fig. 5, Fig. S5)." Which part of Fig. 5? Nothing specific about Fig. 5 is mentioned or explained in the text. As well, why do you need a S fig for almost every fig in the text. This seems to me to be very redundant.

We added part IDs of the figures here and in the other parts. (L283) For each body part, we only included the images where the most clear bromine accumulation was found in the main text. The microstructures of the body parts as well as additional SEM/EDS are shown in the Supplementary. We consider the inclusion of these data to increase the transparency of our data.

Discussion

L??? - ??? – "A possibility [of what?] is the simple...this possibility." This sentence does not make sense. What are the authors trying to say?

We rephrased this section combining with previous paragraph to make it clear that we are trying to list the possible MECHANISMS of bromine accumulation, and to explain why we think active accumulation/secretion by the amphipod is the most likely. (L429)

The accumulation of bromine could be a result of 1) solidification and concentration from sea salt during sample preparation, 2) exogenous adhesion on the amphipods exoskeleton while the amphipod was still alive, or 3) active accumulation and secretion by the amphipod itself….

L??? - ??? – “In terms od small molecules...compound from marine bacteria.” This is a run-on sentence that makes little sense. Please rewrite and break into at least 2 sentences.

This sentence was intended to show three examples of animals possessing bromine-rich compounds, and the sentence was restructured for clarity. (L440)

> Accumulation of bromine has been also found on other marine organisms, and bromine-rich aromatic small molecules have been isolated from marine organisms. For examples, the marine snail *Bolinus bandaris* (a historically important source of Tyrian purple) secretes 6-bromo-3-indolinone derivatives,[36] the tripeptide barettin from the marine sponge *Geodia barretti* has dehydrogenated 6-bromotryptophan,[37] and marine bacteria such as *Pseudomonas bromoutilis* produces pentabrominated phenylpyrrole which is an antibiotic compound.[38,39]

Major Revisions

Main Text

2

L??? – end of first paragraph. There is need to provide a more thorough and exhaustive introduction to the possible impregnation of Crustacean exoskeletons with other materials. Here, several examples from the previous literature should be provided.

We separated the section describing Crustacean exoskeletons and provided an introductory paragraph as requested. (L49)

Methods

Precautions to avoid artificial aluminum signals

L??? - ??? – “...due to three benefits. Firstly, the spatial resolution...reflect the sample’s true elemental composition.” Since this is actually a discussion of the benefits of the process, these should NOT be presented here in the Methods section, but instead should be used to discuss the results and support them as benefits of the process undertaken by the authors. Having them in the Methods does not make sense – they are NOT methods, but explanations of the results.

We moved the sections to the top of “Elemental analysis using electron microscopy” section in Discussion. (L363)

Results

It is never mentioned in the Results how many specimens of H. gigas were actually collected from the expedition and used in the study. This needs to be provided.

This information was added in the Methods. (L108)

L??? – "…1.48 keV (Fig. 2)." Authors need to be specific about what part of Fig. 2 they are referring to; A, B, or C? Also, here in the results, they should refer to each part of Fig. 2, including A, B, and C.
In this same area of the text, the authors mention a plot of two peaks. However, in Fig. 2B, they have two separate plots of peaks. Which is which? Explain both and refer to both in the text. Need to be very specific here.
We specified the parts of Fig. 2 after this sentence and added the explanation of the signal assignments. (L217)

> The intensity around 1.48 keV can therefore be explained without aluminum being present, indicating that the aluminum-like signals we have observed could in fact be bromine (Fig. S4). The presence of bromine was further supported by another signal at 11.92 keV was also observed, corresponding to the K-line of bromine (Fig. 2b bottom).

L??? – "The elemental colocalization…in the cross-section tissues." All of this section should be removed and used in the Discussion section, NOT here in Results.
We removed the sentences concerned, because the equation in the Methods already clearly expresses the content. (L231)

L??? – "…(Fig. 8d…)." There is no "d" labelled in Fig. 8.
Thank you for noting the typo. We corrected it to Fig. 8c. (L337)

L??? – "…the coxal plates (Fig. 7, Fig. S12)…" I do not believe these should be referring back to Fig. 7, but instead to Fig. 9. At this point the ms has a total of 21 figures. This seems to me to be a huge number of figs. Are they all really necessary? I do not know about the supplemental figs because I did not see that they were included with the ms for review.
Thank you for noting the typo. We corrected it accordingly. (L349) The inclusion of these supplementary figures is to increase the transparency of our data, and for the interested readers. We only present the images with most clear bromine accumulation in the main text, but we think providing other images in the supplement helps support our interpretations.

L??? - ??? – "However, salt did not affect the amount of bromine…sea salt." This does not appear to be a valid explanation as to why sea salt would not affect bromine. Are there no bonding interactions, no crystal structure influences?? Explain your point here and provide

support from the literature for your stance.

We intended to state that the amount of bromine is not affected by the adsorption of sea salt in seawater upon sample preparation (drying, dehydration, etc.). This is because bromine anion behaves similarly to chlorine anion. If bromine accumulates on setae by electrostatic or some other noncovalent interactions, more abundant chlorine should also be observed in a significant amount, but the EDS spectra show the intensity of chlorine (Cl K at 2.62 keV) was far below that of bromine (Br L at 1.48 keV). We rewrote the sentences to express our intension more clearly. (L421)

> However, sea salt only appeared as granules, not as surface coatings, judging from the amount of Na and Cl being far lower than that of Br on fine setae (S4–S8 Figs). This indicates that salt did not affect the amount of bromine because the molar amount of bromine is less than 1% of that of sodium, the main content of the sea salt.

General

Figures have many parts and sections, yet authors do not refer to the parts and sections in reporting the results. This makes it VERY confusing as to which part of the figure is being described in the Results text.

We added figure sections in the main text, except for those stated in the first sentence of the paragraph because it was put to guide readers which figure to look at.

3

Again, I do not understand why there are so many supplemental figures – one for every figure presented in the text, and more! Are all of these truly necessary to comprehend the research done?

The inclusion of these supplementary figures is to increase the transparency of our data, and for the interested readers. We only present the images with most clear bromine accumulation in the main text, but we think providing other images in the supplement helps support our interpretations.

In the main text, the main focus was on the elemental composition, especially Br, C, O, Mg, P, and Ca, and cps map. The manuscript also discusses the microscopic histology of the amphipod, which was mostly shown in the Supplementary figures. Additionally, EDS point analysis, which is a semi-quantitative support for the amount of bromine, was shown in the Supplementary figures and tables. Therefore, all of these data are necessary to fully support the manuscript, except for the now-removed Figure S12 that mostly overlapped with Figure 9.

In general, there appears to be a lack of understanding of how to formulate an ms for publication. The authors need to carefully outline the ms so that there is logical flow from one section to the next, and ensure that within the major sections of the paper (i.e. Introduction, Methods, Results, Discussion), that they keep within those boundaries of the sections. Do not put discussion into the Methods, or Results in the Introduction, and so on.

Thank you for the comment and we really appreciated your detailed review and suggestions. We rewrote the manuscript to separate major sections of the paper as suggested, and hope the manuscript is acceptable in the current form.
