# Supplementary material for: Unusual bromine enrichment in the gastric mill and setae of the hadal amphipod Hirondellea gigas
Source: PLoS One. 2022 Aug 4;17(8):e0272032. doi: 10.1371/journal.pone.0272032 (PMC9352070; doi:10.1371/journal.pone.0272032)
Supplement: Attachment — Submitted filename: ReReview of Okada et al.pdf [file pone.0272032.s020.pdf]

Re-review of Unusual bromine enrichment in the gastric mill and setae of the hadal amphipod *Hirondellea gigas* by Okada2, et al.

I have again reviewed the ms by Okada et al, and found that the paper is *much* improved! The logical flow of the paper is now sound, and the methods, results and discussion are all distinct from each other and flow well from one to the next. It was a much more enjoyable paper to read this time around!! I noted the addition of a map with some details on the site where the amphipods were collected, as well as much clarification on the methods of the study. I congratulate the authors on taking the time an effort to carefully re-write the ms.

There are, however, still several small grammatical errors, which I have endeavored to point out and provide suggestions for correcting, in the attached PDF version of the ms with my comments and suggestions on how to correct these in balloons and direct edits to the text.

I believe that once these small errors have been corrected, the ms would be well suited for publication in PLOS. Congratulations again, on really improving the overall presentation of your interesting research.
