# Supplementary material for: Unusual bromine enrichment in the gastric mill and setae of the hadal amphipod Hirondellea gigas
Source: PLoS One. 2022 Aug 4;17(8):e0272032. doi: 10.1371/journal.pone.0272032 (PMC9352070; doi:10.1371/journal.pone.0272032)
Supplement: Attachment — Submitted filename: Response.docx [file pone.0272032.s018.docx]

The reviewer’s comment is shown in red letters.

Reviewer #1: I read your paper with great interest. The authors used detailed image analysis by electron microscopy and X-ray measurements on amphipod samples collected from the difficult-to-reach ultra-deep sea, and found that amphipods do not have an "aluminum shield" as previously thought, but accumulate bromine and magnesium. We believe that these new findings will greatly contribute to the development of deep-sea biology. This paper is judged to be of sufficient value for publication in PlosOne.

Thank you very much for the positive comments.

Reviewer #2: See attached documents provided.
Review of “Unusual bromine enrichment in the gastric mill and setae of the hadal amphipod Hirondellea gigas” by Okada, et al.

Review of “Unusual bromine enrichment in the gastric mill and setae of the hadal amphipod *Hirondellea gigas*” by Okada, et al.

I have reviewed the ms by Okada, et al, regarding the absence of an aluminum gel shield in the deep-water amphipod, *H. gigas* and the presence of bromine, instead, demonstrating the importance of careful and proper SEM analyses of samples for ‘elemental anatomy.’ I found the manuscript (ms) of interest and of potential interest to the PLOS One audience of readers. However, although the work appears to be thorough and meticulously done, demonstrating careful analytical rigor, I have, nevertheless found the writing to be rather simplistic with faults in the logical flow of the paper. It appears more analogous to an undergraduate laboratory report, than a ms for publication, and for the most part, is poorly prepared.

For these and the following reasons, I do not recommend the publication of this work until it has been carefully recrafted and revised according to my comments below. I have provided a PDF of the text with my many small comments and edits, which I recommend the authors carefully consider and revise the ms accordingly. In addition, I am providing in this document, a list of both Minor and Major areas of revisions for consideration by the authors. I hope the comments provided facilitate the revising of the ms and are helpful to the authors. I believe that once revised, the paper would be of interest and use to other researchers with interests in the area of deep see arthropods and their anatomy and physiology.

Additionally, not having read the Instructions for Authors, I wonder why the ms does not have line numbers and why the many supplemental figures and tables were not provided for the purposes of review. Having line numbers facilitates a much easier review process for the reviewer and authors. I recommend that manuscripts NOT be sent to reviewers by the Editors unless continuous line numbers have been included!!

Thank you for reading and evaluating our papers. The pdf you kindly attached was greatly helpful to revise our manuscript. I apologize for the absence of line numbers; which is my mistake. We have revised the manuscript following your comments and suggestions, and hope the revised manuscript is acceptable for publication.

Minor Revisions

There are a plethora of small grammatical errors in the text which I have endeavored to correct (as far as I could understand some of the sentences written). I recommend the authors have a native science writer review the text before resubmitting the revised ms to the journal (or any journal).

We have rewritten the manuscript and checked it with a native English speaker.

Abstract

L??? – the code EDS is used. However, EDS is never spelled out in the prior text. This needs to be spelled out in full the first time it is used, as here in this sentence.

We spelled out EDS accordingly. (L31)

Our results … signals in energy-dispersive X-ray spectroscopic analysis

Main Text

L??? – second para. The autors use the term “megafauna” for H. gigas. This is incorrect. H. gigas would be considered macrofauna, not megafauna. Please check the definitions of these.

We agree and changed to macrofauna. (L68)

L??? – the authors state, “The aluminum shield was expected to...” Who was it that was expecting this, and what is the citation/reference for this?

Those who found the ‘aluminum shield’ expected this. The reference 8 was added, and because this reference was cited continuously for three times, the next sentence was slightly modified.

However, their results were inconclusive… (L77)

1

L??? - ??? – “The aluminum shield...Challenger Deep.” This sentence is long and convoluted. It makes little sense, and also needs a reference at the end to state who was expecting this gel shield.

We clarified the sentence and added a reference number. (L73)

Based on the combination of energy-dispersive X-ray analysis (EDS) under scanning electron microscopy (SEM) and aluminum extraction experiments from the sediment of Challenger Deep, Kobayashi et al. expected the aluminum shield to be in a form of gel.^8^

L??? - ??? – first part of first paragraph on pg. 3. “In order to confirm...artefacts.” This sentence should be moved to the end of the Introduction section as a segway into the methods section.

We moved the sentence accordingly. (L74)

L??? - ??? – “There are two possibilities where aluminum...latter two being unused in animals.” This section should be moved to the top of the first full paragraph on Pg. 3.

This sentence is now at the top of the first paragraph, after moving the sentence as suggested above. (L87)

L??? - ??? “Our detailed analysis revealed...harden the hard parts.” This entire section belongs in the Results section, not in the introduction section. It is not part of the literature review or the justifications for the study.

We shortened the sentence to guide readers to our main goal of the bromine accumulation. (L94)

Here, we report our findings from detailed electron microscopy observations of *H. gigas*, revealing the absence of aluminum on the exoskeleton and instead the accumulation of bromine on hard tissues as the sclerotized spines of its gastric mill components, a rare example of preferentially secreted bromine in the hard parts of animals.

Methods

Amphipod Samples

L??? - ??? – it would be helpful to provide a map of the study location as Fig. 1.

We added the map, a photo of the bait trap, and the animal in Fig. S1. (L113)

L??? - ???. In this section, no mention is made of how many amphipod samples were actually collected in total, and neither is a total sample size mentioned in the Results.

Though over 200 amphipods were collected, only three were rapidly frozen using nitrogen slurry and used in the present study. Among the three frozen specimens, one was embedded in resin, and other two were dissected. This information is now added in Methods (L108)

Over 200 individuals (Fig. S1b) were collected, of which three individuals were used for this study. The amphipods were immediately immersed …

Electron microscope instruments.

L??? – “...2k x 2K...” us the multiplication symbol (×), not the letter “x”

We revised the symbol accordingly. (L123, 163)

L??? – “The dehydrated amphipod was replaced with QY-1...” Is this really what the authors meant to say; that the actual animal was replaced with QY-1? I do not understand this. Should this mean something, such as “fluid from the amphipod was replaced by QY-1...”?

We simplified the resin embedding procedure as follows: (L131)

The dehydrated amphipod was immersed into QY-1…

L??? “...HighScore 3.0.5...” Is this hardware or software? It is not clear and not stated. Please clarify.

This is a software and we clarified accordingly (L171).

… performed on the software HighScore 3.0.5 (PANalytical).

L??? – “...maxilla (Fig. 5, Fig. S5).” Which part of Fig. 5? Nothing specific about Fig. 5 is mentioned or explained in the text. As well, why do you need a S fig for almost every fig in the text. This seems to me to be very redundant.

We added part IDs of the figures here and in the other parts. (L283) For each body part, we only included the images where the most clear bromine accumulation was found in the main text. The microstructures of the body parts as well as additional SEM/EDS are shown in the Supplementary. We consider the inclusion of these data to increase the transparency of our data.

Discussion

L??? - ??? – “A possibility [of what?] is the simple...this possibility.” This sentence does not make sense. What are the authors trying to say?

We rephrased this section combining with previous paragraph to make it clear that we are trying to list the possible MECHANISMS of bromine accumulation, and to explain why we think active accumulation/secretion by the amphipod is the most likely. (L429)

The accumulation of bromine could be a result of 1) solidification and concentration from sea salt during sample preparation, 2) exogenous adhesion on the amphipods exoskeleton while the amphipod was still alive, or 3) active accumulation and secretion by the amphipod itself….

L??? - ??? – “In terms od small molecules...compound from marine bacteria.” This is a run-on sentence that makes little sense. Please rewrite and break into at least 2 sentences.

This sentence was intended to show three examples of animals possessing bromine-rich compounds, and the sentence was restructured for clarity. (L440)

Accumulation of bromine has been also found on other marine organisms, and bromine-rich aromatic small molecules have been isolated from marine organisms. For examples, the marine snail *Bolinus bandaris* (a historically important source of Tyrian purple) secretes 6-bromo-3-indolinone derivatives,[36] the tripeptide barettin from the marine sponge *Geodia barretti* has dehydrogenated 6-bromotryptophan,[37] and marine bacteria such as *Pseudomonas bromoutilis* produces pentabrominated phenylpyrrole which is an antibiotic compound.[38,39]

Major Revisions

Main Text

2

L??? – end of first paragraph. There is need to provide a more thorough and exhaustive introduction to the possible impregnation of Crustacean exoskeletons with other materials. Here, several examples from the previous literature should be provided.

We separated the section describing Crustacean exoskeletons and provided an introductory paragraph as requested. (L49)

Methods

Precautions to avoid artificial aluminum signals

L??? - ??? – “...due to three benefits. Firstly, the spatial resolution...reflect the sample’s true elemental composition.” Since this is actually a discussion of the benefits of the process, these should NOT be presented here in the Methods section, but instead should be used to discuss the results and support them as benefits of the process undertaken by the authors. Having them in the Methods does not make sense – they are NOT methods, but explanations of the results.

We moved the sections to the top of “Elemental analysis using electron microscopy” section in Discussion. (L363)

Results

It is never mentioned in the Results how many specimens of H. gigas were actually collected from the expedition and used in the study. This needs to be provided.

This information was added in the Methods. (L108)

L??? – “...1.48 keV (Fig. 2).” Authors need to be specific about what part of Fig. 2 they are referring to; A, B, or C? Also, here in the results, they should refer to each part of Fig. 2, including A, B, and C.

In this same area of the text, the authors mention a plot of two peaks. However, in Fig. 2B, they have two separate plots of peaks. Which is which? Explain both and refer to both in the text. Need to be very specific here.

We specified the parts of Fig. 2 after this sentence and added the explanation of the signal assignments. (L217)

The intensity around 1.48 keV can therefore be explained without aluminum being present, indicating that the aluminum-like signals we have observed could in fact be bromine (Fig. S4). The presence of bromine was further supported by another signal at 11.92 keV was also observed, corresponding to the K-line of bromine (Fig. 2b bottom).

L??? – “The elemental colocalization...in the cross-section tissues.” All of this section should be removed and used in the Discussion section, NOT here in Results.

We removed the sentences concerned, because the equation in the Methods already clearly expresses the content. (L231)

L??? – “...(Fig. 8d...).” There is no “d” labelled in Fig. 8.

Thank you for noting the typo. We corrected it to Fig. 8c. (L337)

L??? – “...the coxal plates (Fig. 7, Fig. S12)...” I do not believe these should be referring back to Fig. 7, but instead to Fig. 9. At this point the ms has a total of 21 figures. This seems to me to be a huge number of figs. Are they all really necessary? I do not know about the supplemental figs because I did not see that they were included with the ms for review.

Thank you for noting the typo. We corrected it accordingly. (L349) The inclusion of these supplementary figures is to increase the transparency of our data, and for the interested readers. We only present the images with most clear bromine accumulation in the main text, but we think providing other images in the supplement helps support our interpretations.

L??? - ??? – “However, salt did not affect the amount of bromine...sea salt.” This does not appear to be a valid explanation as to why sea salt would not affect bromine. Are there no bonding interactions, no crystal structure influences?? Explain your point here and provide support from the literature for your stance.

We intended to state that the amount of bromine is not affected by the adsorption of sea salt in seawater upon sample preparation (drying, dehydration, etc.). This is because bromine anion behaves similarly to chlorine anion. If bromine accumulates on setae by electrostatic or some other noncovalent interactions, more abundant chlorine should also be observed in a significant amount, but the EDS spectra show the intensity of chlorine (Cl K at 2.62 keV) was far below that of bromine (Br L at 1.48 keV). We rewrote the sentences to express our intension more clearly. (L421)

However, sea salt only appeared as granules, not as surface coatings, judging from the amount of Na and Cl being far lower than that of Br on fine setae (S4–S8 Figs). This indicates that salt did not affect the amount of bromine because the molar amount of bromine is less than 1% of that of sodium, the main content of the sea salt.

General

Figures have many parts and sections, yet authors do not refer to the parts and sections in reporting the results. This makes it VERY confusing as to which part of the figure is being described in the Results text.

We added figure sections in the main text, except for those stated in the first sentence of the paragraph because it was put to guide readers which figure to look at.

3

Again, I do not understand why there are so many supplemental figures – one for every figure presented in the text, and more! Are all of these truly necessary to comprehend the research done?

The inclusion of these supplementary figures is to increase the transparency of our data, and for the interested readers. We only present the images with most clear bromine accumulation in the main text, but we think providing other images in the supplement helps support our interpretations.

In the main text, the main focus was on the elemental composition, especially Br, C, O, Mg, P, and Ca, and cps map. The manuscript also discusses the microscopic histology of the amphipod, which was mostly shown in the Supplementary figures. Additionally, EDS point analysis, which is a semi-quantitative support for the amount of bromine, was shown in the Supplementary figures and tables. Therefore, all of these data are necessary to fully support the manuscript, except for the now-removed Figure S12 that mostly overlapped with Figure 9.

In general, there appears to be a lack of understanding of how to formulate an ms for publication. The authors need to carefully outline the ms so that there is logical flow from one section to the next, and ensure that within the major sections of the paper (i.e. Introduction, Methods, Results, Discussion), that they keep within those boundaries of the sections. Do not put discussion into the Methods, or Results in the Introduction, and so on.

Thank you for the comment and we really appreciated your detailed review and suggestions. We rewrote the manuscript to separate major sections of the paper as suggested, and hope the manuscript is acceptable in the current form.
