# Supplementary material for: Unusual bromine enrichment in the gastric mill and setae of the hadal amphipod Hirondellea gigas
Source: PLoS One. 2022 Aug 4;17(8):e0272032. doi: 10.1371/journal.pone.0272032 (PMC9352070; doi:10.1371/journal.pone.0272032)
Supplement: Attachment — Submitted filename: Response.docx [file pone.0272032.s021.docx]

Journal Requirements:

Please review your reference list to ensure that it is complete and correct. If you have cited papers that have been retracted, please include the rationale for doing so in the manuscript text, or remove these references and replace them with relevant current references. Any changes to the reference list should be mentioned in the rebuttal letter that accompanies your revised manuscript. If you need to cite a retracted article, indicate the article’s retracted status in the References list and also include a citation and full reference for the retraction notice.

We have re-checked the reference list and modified refs 7 and 23. Ref 7 does not have a volume number as this journal does not use volume number anymore, and we removed the incorrect “0” that was in the previous version. Unnecessary DOI information was removed from Ref 23. Other references were kept unchanged.

Reviewer #2: The ms is VERY MUCH improved in readability and logical flow. I congratulate the authors on really paying attention to the review comments I provided in the original review. The ms needs now only minor grammatical corrections, for which I have included suggestions and edits in the PDF of the revised text of ms attached.

Thank you very much for reading our paper again and all of your efforts for minor grammatical corrections! We have gone through the manuscript and made all corrections you suggested in the attached PDF file.

Reviewer #3: The authors carefully examined the minor element composition of the hadal amphipod and found that the amphipod showed bromine enrichment in the gastric mill and setae, but no evidence for Al enrichment. This is my first evaluation of this MS, but I took over the review comments provided by earlier reviewers. The authors properly answered the reviewer's comments. I also felt that the manuscript is properly written and the scientific quality of the manuscript meets the standard of Plos One publication.
We appreciated the Reviewer’s efforts in going through our manuscript and for the suggestions provided.

However, there are still some mistakes in the manuscript that should be corrected before publication, even though the reviewer pointed out the English editing. I only listed the mistakes I could find, but I strongly recommend that all of the authors carefully multiple-check the manuscript, given the Plos One policy is that "PLOS ONE does not copyedit accepted manuscripts, so the language in submitted articles must be clear, correct, and unambiguous. Any typographical or grammatical errors should be corrected at revision". English editing is not the job of a reviewer. The authors should English editing and proofreading with their own responsibility.
Thanks to the reviewer 2 who kindly edited much of our English, the language has been improved in this revised version. We then also went through the manuscript again and removed errors where we found them.

L20, should be corrected properly.

We removed the multiple “including”.

L80, should be corrected properly.
We corrected to:
Furthermore, samples were not covered with conductive coatings for imaging and EDS analysss to prevent the sample from charging-up, …

L131, Please explain what is QY-1.
This is a common agent for resin embedding procedure, and we have clarified the content as 1-*n*-butoxy-2,3-epoxypropane.

L132, laser should be razor?

Thank you for noting the typo. We corrected accordingly.
